# Reversible and Irreversible Regioselective Alkyne Insertion into a Silyl‐Substituted Stannylene

**DOI:** 10.1002/chem.202502103

**Published:** 2025-07-28

**Authors:** Aidan J. Murray, Lewis L. Wales, Agamemnon E. Crumpton, Maximilian Dietz, Mathias A. Ellwanger, Andreas Heilmann, Job J. C. Struijs, Simon Aldridge

**Affiliations:** ^1^ Inorganic Chemistry Laboratory Department of Chemistry University of Oxford South Parks Road Oxford OX1 3QR UK

**Keywords:** alkyne, insertion, reversibility, stannylene, tin

## Abstract

A range of aryl‐ and alkyl‐ substituted alkynes has been shown to insert regio‐selectively into the Sn─Si bond of the electron‐rich aryl(silyl)stannylene, Ar^Mes^SnSi(SiMe_3_)_3_ (Ar^Mes^ = 2,6‐Mes_2_C_6_H_3_, Mes = 2,4,6‐Me_3_C_6_H_2_) to generate a series of vinyl‐stannylene products. In all cases, the product features a *syn* arrangement of Sn and Si‐containing groups about the resulting carbon–carbon double bond; in the case of unsymmetrical alkynes, the more sterically bulky group is exclusively incorporated in the 1‐position (i.e., proximal to Sn). Remarkably, insertion is shown to occur reversibly in the cases of 3‐hexyne and trimethylsilylacetylene. The thermodynamic parameters associated with these processes have been determined by variable temperature NMR spectroscopy, and the activation barriers associated with the key mechanistic steps elucidated by quantum mechanical methods.

## Graphical abstract

Alkyne insertion into the Sn─Si bond of a silyl‐substituted stannylene occurs regio‐selectively and ‐ in the cases of more sterically encumbered substrates ‐ reversibly

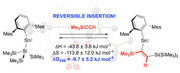

## 1 Introduction

Stannylenes are the tin analogues of carbenes; they have the generic formula X_2_Sn, and adopt monomeric structures when X is a sterically demanding substituent.^[^ ^1^, ^2^, ^3^, ^4^ ^]^ These species have seen considerable research attention and evidence differing levels of reactivity that can be related (at least in part) to the magnitude of the energy separation between the primarily 5*s*‐based HOMO and the 5*p*‐based LUMO.^[^ ^5^, ^6^ ^]^ Stannylenes featuring a relatively narrow HOMO‐LUMO gap can be engineered through choice of X substituents, and have been shown to activate a range of small molecules featuring E─H bonds (E = H, ArCH_2_, N, B, O, P, Si), as well as ethene, CO_2_, and 1,3‐unsaturated systems.^[^ ^7^, ^8^, ^9^, ^10^, ^11^, ^12^, ^13^, ^14^, ^15^, ^16^, ^17^, ^18^, ^19^ ^]^

**Figure 1:**
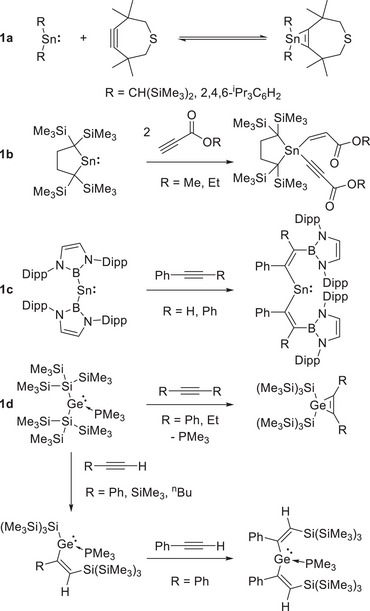
Selected examples of previously reported reactions of alkynes with stannylenes and germylenes: cycloaddition (**a**,**d**), oxidative addition (**b**), insertion (**c**,**d**). (Dipp = 2,6‐^i^Pr_2_C_6_H_3_).

Despite the abundant and varied reactivity displayed by stannylenes, their reactivity toward alkynes remains comparatively underexplored. Sita et al. reported the first such reactivity studies, uncovering [2 + 1] cycloaddition processes that reversibly generate stanna‐cyclopropenes (Figure 1a).^[^ ^20^, ^21^ ^]^ More recently, it was reported that a cyclic dialkylstannylene reacts with two equivalents of propynoate esters to give Sn(IV) products via C─H activation followed by hydrostannylation (Figure 1b); no analogous reaction was observed with common terminal or internal alkynes such as phenylacetylene and diphenylacetylene.^[^ ^22^ ^]^ The most common reaction mode, however, appears to be insertion into the Sn─X bond(s). Jones and Power have both reported that (dimeric) tin(II) hydrides will hydrometallate alkynes, forming aryl(vinyl)stannylene products.^[^ ^23^, ^24^ ^]^ Similar reactivity has also been reported for an aryl(germyl)stannylene, while double insertion of both phenylacetylene and diphenylacetylene was shown to occur into the Sn─B bonds of a bis(boryl) stannylene, creating a bis(vinyl) stannylene (Figure 1c).^[^ ^25^, ^26^ ^]^ Diaryl‐stannylenes have also been shown to insert a range of alkynes under thermal conditions via a radical mechanism.^[^ ^27^ ^]^

The tris(trimethyl)silyl, or “hypersilyl” ligand (─Si(SiMe_3_)_3_) is a sterically bulky, electron‐releasing substituent which has been used extensively in low‐valent group 14 chemistry.^[^ ^28^ ^]^ Thus, for example, the phosphine‐stabilized bis(hypersilyl)germylene ((Me_3_Si)_3_Si)_2_Ge·PMe_3_, reported by Marchner et al., has been shown to undergo [2 + 1] cycloaddition with the internal alkynes diphenylacetylene and 3‐hexyne, losing PMe_3_ and forming germacyclopropene species (Figure 1d).^[^ ^29^, ^30^ ^]^ Terminal alkynes, by contrast, undergo insertion (of one equivalent) into a Ge─Si bond to form a series of phosphine‐stabilized (vinyl)germylenes.

In 2022, the novel electron‐rich aryl(silyl)stannylene Ar^Mes^SnSi(SiMe_3_)_3_ (**1**; Ar^Mes^ = 2,6‐Mes_2_C_6_H_3_, Mes = 2,4,6‐Me_3_C_6_H_2_) was reported.^[^ ^31^ ^]^ With kinetic stability toward dimerization provided by the bulky hypersilyl and terphenyl ligands, this species was found to be highly reactive toward organic azides, leading to the formation of stanna‐imines featuring Sn═N double bonds.^[^ ^31a^ ^]^ As an extension of this work we wanted to explore the reactivity of **1** toward other unsaturated substrates; its reactivity toward a range of alkynes is reported herein.

## 2 Results and Discussion

### 2.1 Irreversible Alkyne Insertion

**Scheme 1:**
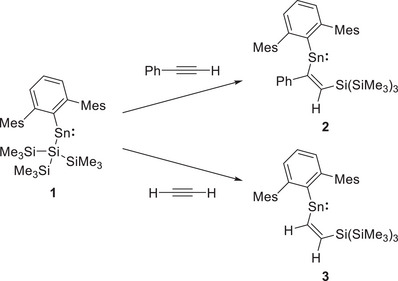
Reaction of aryl(silyl)stannylene **1** with phenylacetylene and acetylene to form insertion products **2** and **3**, respectively.

Addition of excess phenylacetylene to a solution of **1** in toluene at room temperature results in an immediate color change from green to purple, with NMR studies implying insertion of a single equivalent of phenylacetylene into the Sn─Si bond to form aryl(vinyl)stannylene **2** (Scheme 1). Analysis of the reaction mixture by ^1^H NMR spectroscopy shows a new set of signals, the most notable of which is a single proton at *δ*_H_ = 7.49 ppm corresponding to an alkenic CH environment. This proton displays single‐ and multiple‐bond correlations with ^13^C NMR resonances at *δ*_C_ = 179.7 and 218.3 ppm, respectively, representing the two new alkenic carbon environments. The ^29^Si NMR resonances at *δ*_Si_ = −12.9 and −87.2 ppm are also similar to those belonging to the hypersilyl‐appended vinyl(germylene)s reported by Marchner et al., while the ^119^Sn NMR shift (*δ*_Sn_ = 1690 ppm) is within the range of previously reported aryl(vinyl)stannylenes (*δ*_Sn_ = 1349–1877 ppm).^[^ ^24^, ^25^, ^27^, ^29^, ^30^ ^]^

**Figure 2:**
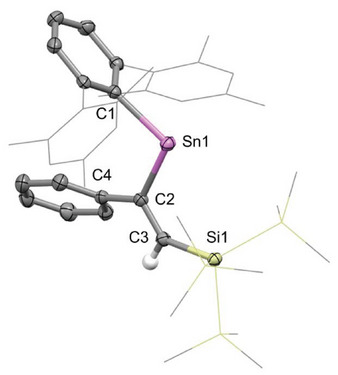
Molecular structure of **2** as determined by X‐ray crystallography. Thermal ellipsoids are set at the 50% probability level. All hydrogen atoms except for the alkenic H are omitted for clarity. The Mes and SiMe_3_ groups are shown in wireframe for clarity. Key bond lengths (Å), angles (°), and torsions (°): Sn1–C1 2.219(2), Sn1─C2 2.209(2), C2─C3: 1.348(2), C2─C4 1.484(3), C3─Si1 1.898(2), C1─Sn1─C2 103.79(8), Sn1─C2─C3─Si1 0.1(3).

Characterization of **2** in the solid state by single crystal X‐ray diffraction (Figure 2) confirms the expected connectivity, featuring a *syn* arrangement of Sn‐ and Si‐ groups about the double bond, with the more sterically bulky phenyl group adjacent to the tin atom. To our knowledge, this represents the first known example of alkyne insertion into a silyl‐substituted stannylene. The regio‐ and stereochemistry is consistent with previously reported alkyne insertions into stannylenes and germylenes.^[^ ^23^, ^25^, ^26^, ^29^, ^30^ ^]^ Structurally, the C1─Sn1─C2 bond angle (103.79(8)°) is narrower than that of starting material **1** (109.75(6)°), presumably a consequence of relief of steric strain around the tin center. The C1─Sn1 and C2─Sn1 bond distances (2.219(2) and 2.209(2) Å, respectively) lie within the ranges previously reported for aryl(vinyl)stannylenes (2.198(3) to 2.243(3) Å).^[^ ^24^, ^25^, ^27^ ^]^ The C2─C3 distance (1.348(2) Å) and the coplanarity of Sn and Si groups (as defined by a Sn1─C2─C3─Si1 torsion angle of 0.1(3)°) are fully consistent with the presence of a double bond between C2 and C3.

Similar reactivity is seen between **1** and acetylene: exposure of a C_6_D_6_ solution to ca. 1 atm. of HCCH leads to an immediate color change from green to red. The ^1^H NMR spectrum shows “roofed” doublet signals corresponding to two protons (at *δ*_H_ = 9.50 and 8.06 ppm), which show single bond correlations with resonances in the ^13^C NMR spectrum at *δ*_C_ = 202.0 and 177.5 ppm, respectively. These signals correspond to the alkenic environments of the insertion product **3** (Scheme 1). The ^3^ *J*_HH_ coupling constant of 16.7 Hz is more typical of a *trans*‐alkene, for which couplings normally lie in the range 14–20 Hz, while those of *cis*‐alkenes typically range 6 to 14 Hz.^[^ ^32^ ^]^ However, Mizuhata et al. have reported an alkenic ^3^ *J*_HH_ coupling constant of 18.9 Hz for a *cis*‐(Sn─C(H)═C(H)─Si) configuration, while Seyferth et al. reported that *trans*‐1‐trimethylsilyl‐2‐trimethylstannylethene (*trans*‐Me_3_SnC(H)═C(H)SiMe_3_) possesses a ^3^ *J*_HH_ coupling constant of 22.0 Hz. On this basis, and on the back of structural and computational data disclosed here which reveal exclusively *syn* selectivity for a range of alkyne insertions, it is thus proposed that the reaction between **1** and acetylene most likely also affords the *syn*‐alkene product **3** as shown in Scheme 1. Confirmation by X‐ray crystallography could not be obtained due to the very high solubility of **3** in hydrocarbon solvents and our consequent failure to obtain suitable single crystals.

**Scheme 2:**
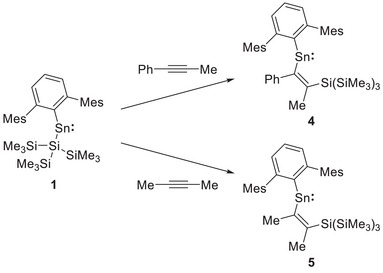
Reaction of **1** with 1‐phenyl‐1‐propyne and 2‐butyne to form insertion products **4** and **5**, respectively.

Internal alkynes were also investigated. No reaction was seen on addition of diphenylacetylene or bis(trimethylsilyl)‐ acetylene to a solution of **1** in C_6_D_6_, even after heating at 80 °C for 5 days. Slow conversion was, however, observed with 1‐phenyl‐1‐propyne (PhCCMe) and 2‐butyne (MeCCMe) (Scheme 2), with a color change from green to purple observed for each over the course of 16 hours. In the former case, the ^1^H NMR spectrum shows the appearance of a new 3H singlet at *δ*_H_ = 1.72 ppm, correlated through multiple bonds to ^13^C signals at *δ*_C_ = 180.3 and 216.2 ppm, belonging to the MeC═C moiety of PhCCMe insertion product **4** (Scheme 2). In the case of MeCCMe two 3H singlets at *δ*_H_ = 2.53 and 1.87 ppm show couplings to the ^13^C signals at *δ*_C_ = 210.2 and 182.0 ppm, belonging to the MeC═CMe moiety in product **5** (Scheme 2).

**Figure 3:**
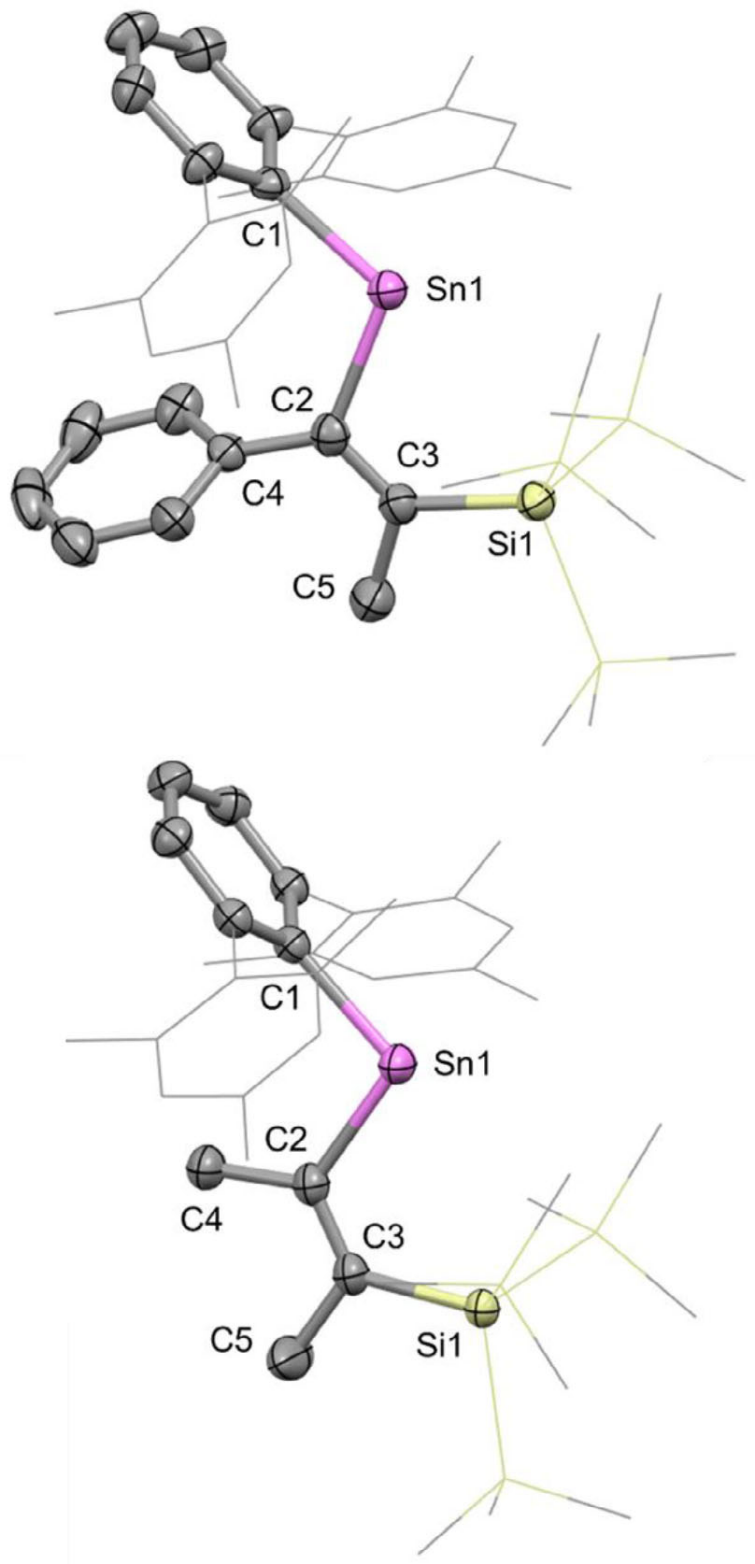
Molecular structures of **4** (upper) and **5** (lower) as determined by X‐ray crystallography. Thermal ellipsoids are set at the 30% and 50% probability level, respectively. Hydrogen atoms are omitted for clarity. The Mes and SiMe_3_ groups are depicted in wireframe for clarity. Key bond lengths (Å), angles (°), and torsions (°): (for **4**) Sn1─C1 2.214(3), Sn1─C2 2.197(4), C2─C3 1.355(5), C2─C4 1.48(4), C3─C5 1.53(4), C3─Si1 1.929(4), C1─Sn1─C2: 105.6(1), Sn1─C2─C3─Si1 11.7(5). (for **5**) Sn1─C1 2.200(3), Sn1─C2 2.163(2), C2─C3 1.347(2), C2─C4 1.526(3), C3─C5 1.513(4), C3─Si1 1.934(2), C1─Sn1─C2: 100.33(2), Sn1─C2─C3─Si1 0.000(3).

Both **4** and **5** proved amenable to study by X‐ray crystallography, using single crystals obtained from pentane and toluene solution, respectively. In the case of **4** (Figure 3), there are four molecules in the asymmetric unit; the mean C1─Sn1─C2 bond angle across the four is 105.6(1)°. The corresponding angle measured for **5** is slightly narrower (100.33(8)°), presumably due to the smaller steric profile of the methyl substituent (compared to phenyl) adjacent to the tin center. The C1─Sn1 bond distances of 2.214(3) (mean) and 2.200(3) Å (for **4** and **5**, respectively) and the corresponding Sn1─C2 distances of 2.197(4) (mean) and 2.163(2) Å are similar to those of **2** (2.219(2) and 2.209(2) Å, respectively). Consistently, the C2─C3 distances (1.355(5) and 1.347(4) Å, respectively), and Sn1─C2─C3─Si1 torsion angles of 0.000(3)° and 11.7(5)°, respectively, are also as expected for the alkenic *syn*‐Sn(R)C═C(Me) unit.

### 2.2 Reversible Alkyne Insertion

Interestingly, analysis of (isolated) samples of **4** on prolonged storage (>48 hours) reveal partial reversion to stannylene **1** and free PhCCMe. The slow kinetics associated with this potential equilibration process (and the decomposition of **4** on warming to temperatures above ca. 50 °C) prevented us from probing the associated thermodynamic parameters by van't Hoff analysis. However, we were prompted on this basis to search for other examples of reversible alkyne uptake.

**Scheme 3:**
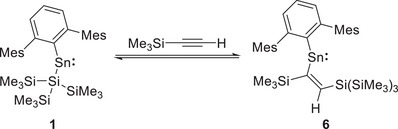
Reversible reaction of **1** with trimethylsilylacetylene to form **6**.

**Figure 4:**
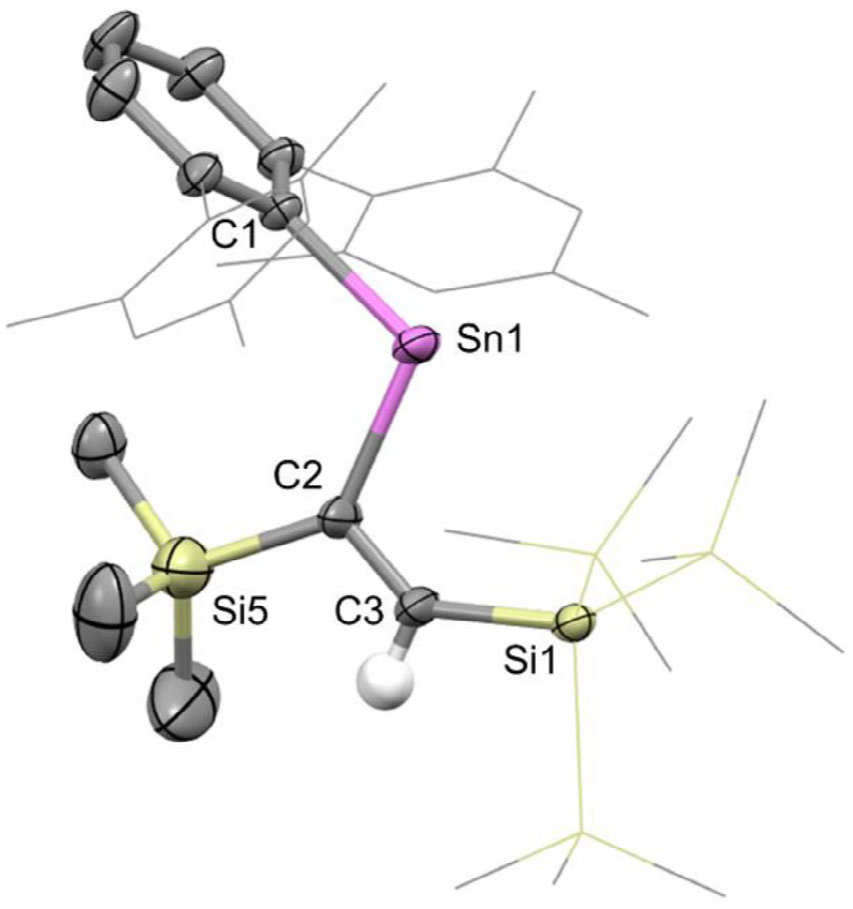
Molecular structure of **6** as determined by X‐ray crystallography. Thermal ellipsoids are set at the 50% probability level. All hydrogen atoms except for the alkenic proton are omitted for clarity. The Mes and SiMe_3_ groups are in wireframe for clarity. Key bond lengths (Å), angles (°), and torsions (°): Sn1─C1 2.210(6), Sn1─C2 2.190(4), C2─C3: 1.343(9), C2─Si5 1.898(5), C1─Sn1─C2 105.3(2), Sn1─C2─C3─Si1 2.1(8).

The reaction of **1** with trimethylsilylacetylene (Me_3_SiCCH) is reversible, and provides a suitable platform for VT‐NMR studies. Addition of excess alkyne to a solution of **1** in C_6_D_6_ results in a color change to deep blue over the course of 2 hours. The ^1^H NMR spectrum of the reaction mixture shows a new set of signals in addition to those of **1**, the most informative of which is a single alkenic CH resonance at *δ*_H_ = 9.16 ppm, correlated to ^13^C NMR signals at *δ*_C_ = 223.1 and 182.0 ppm, belonging to product **6** (Scheme 3). Even in the presence of a large excess of trimethylsilylacetylene, the reaction does not go to completion. The relative amounts of **1**, **6,** and trimethylsilylacetylene remain unchanged over a period of several days. Chemical evidence of reversibility was provided by the addition of phenylacetylene to the reaction mixture, which resulted in an immediate color change to purple, the disappearance of the ^1^H NMR signals belonging to **1** and **6** and the appearance of resonances due to PhCCH‐insertion product **2**. In order to obtain isolable (crystalline) samples of **6**, stannylene **1** was dissolved in neat trimethylsilylacetylene. Slow evaporation from this solution at room temperature yielded blue crystals of the product **6**, which confirmed its connectivity through diffraction studies (Figure 4).

As with the cases detailed above, a *syn*‐alkene is shown to be formed, with the more sterically demanding SiMe_3_ group (cf. H) being incorporated at the 1‐position, that is, proximal to Sn. The C1─Sn─C2 bond angle of 105.3(2)° and C1─Sn1 and Sn─C2 distances of 2.210(6) and 2.190(4) Å, respectively, are similar to those measured for **2**, **4,** and **5**. This is also true for the C2─C3 distance (1.343(9) Å) and the Sn1─C2─C3─Sn1 torsion angle (2.1(8)°), reflecting the structural uniformity in this class of compound.

**Figure 5:**
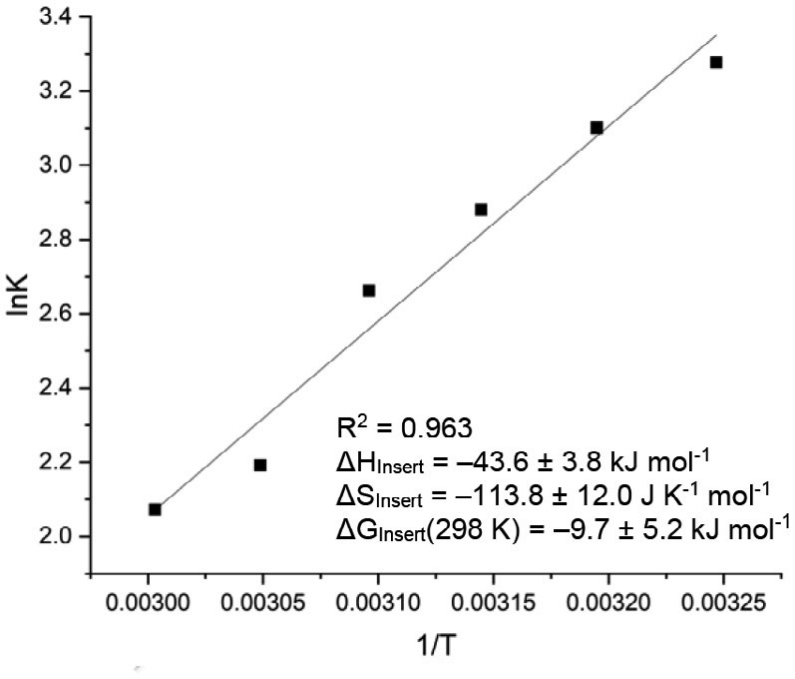
Van't Hoff plot with calculated thermodynamic parameters for the reaction between **1** and trimethylsilylacetylene to produce **6**.

Quantification of the reversibility of the formation of **6** was targeted through variable temperature (VT) ^1^H NMR experiments. Using 3.3 equivalents of alkyne in C_6_D_6_ solution, the proportion of **6** in solution was observed to decrease from 76% to 50% as the temperature of the reaction mixture was increased from 35 to 60 °C, confirming not only the reversibility of the reaction but also its exothermic nature. A van't Hoff analysis was carried out using the integrated intensities from ^1^H NMR experiments in this temperature range (Figure 5). From these data, the following thermodynamic parameters were obtained for alkyne insertion: Δ*H* = −43.6 ± 3.8 kJ mol^−1^ and Δ*S* = −113.8 ± 12.0 J K^−1^ mol^−1^.

**Scheme 4:**
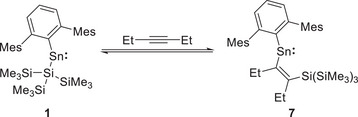
Reversible reaction of **1** with 3‐hexyne to form product **7**.

This behavior is not restricted to trimethylsilylacetylene. In contrast to the irreversible uptake of 2‐butyne (Scheme 2), the reaction of **1** with 3‐hexyne, EtCCEt, results in a reversible reaction similar to that observed with Me_3_SiCCH. Addition of excess 3‐hexyne to a solution of **1** in C_6_D_6_ results in a color change to purple over the course of 16 hours. Beyond the residual peaks of **1**, the ^1^H NMR spectrum of the reaction mixture shows two new triplets at *δ*_H_ = 0.85 and 0.81 ppm and two quartets at *δ*_H_ = 2.31 and 2.15 ppm that belong to the ethyl environments of insertion product **7** (Scheme 4). The ^119^Sn NMR shift of *δ*_Sn_ = 1694 ppm (measured in neat 3‐hexyne) is also consistent with the resonances for aryl(vinyl)stannylenes **2**, **4**, and **5**. We hypothesize that **7** is also likely to be the *syn*‐alkene isomer, but evidence could not be obtained from crystallography to confirm its geometry due to the high solubility of **7** in neat 3‐hexyne and our inability to obtain single crystals. From a van't Hoff analysis based on ^1^H VT NMR experiments (Figure S2), the following values were obtained for the insertion of 3‐hexyne: Δ*H* = −38.7 ± 6.1 kJ mol^−1^ and Δ*S* = −116.4 ± 18.3 J K^−1^ mol^−1^.

The data obtained for alkyne uptake by **1** to give **6** and **7** imply free energy changes at 298 K which are negative, but close to zero (Δ*G*_298_ = −9.7 ± 5.2 and −4.0 ± 8.2 kJ mol^−1^, respectively), consistent with the experimentally observed reversibility in each case. The associative nature of the reaction is borne out in the similar (negative) entropies of reaction (Δ*S* = −113.8 ± 12.0 and −116.4 ± 18.3 J K^−1^ mol^−1^), while the marginally greater exothermicity for the formation of **6** compared to **7** (Δ*H* = −43.6 ± 3.8 vs. −38.7 ± 6.1 kJ mol^−1^) is the origin of its greater favorability, and is presumably due to the reduced steric bulk geminal to the hypersilyl substituent (H vs. Et).

### 2.3 Quantum Chemical Calculations

**Scheme 5:**
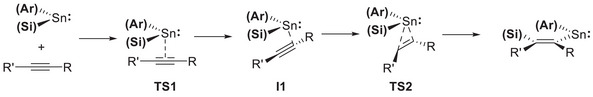
Generic mechanism for alkyne insertion into the Sn─Si bond of **1**. **(Ar)** = Ar^Mes^; **(Si)** = Si(SiMe_3_)_3_.

**Figure 6:**
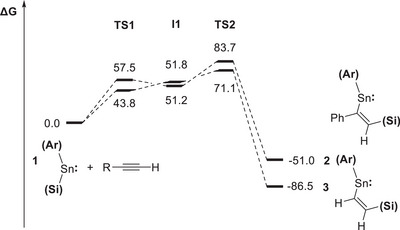
Free energy profile (in kJ mol^−1^) for the reactions of **1** with phenylacetylene and acetylene to produce **2** and **3**, respectively.

**Figure 7:**
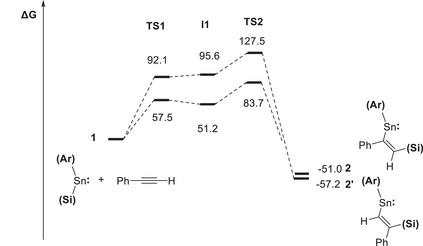
Free energy profile (in kJ mol^−1^) for the insertion of phenylacetylene to give either **2** or its regioisomer **2′**.

**Figure 8:**
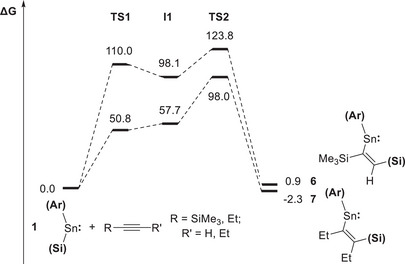
Free energy profile (in kJ mol^−1^) for the reactions of **1** with trimethylsilylacetylene and 3‐hexyne to produce **6** and **7**, respectively.

Given previous reports that metalla‐cyclopropene species can be formed by [2 + 1] cycloaddition reactions between metallylenes and alkynes (e.g., Scheme 1a), we hypothesized that the insertion mechanism might involve initial formation of a metalla‐cyclopropene, followed by migration of the silyl ligand to the least sterically hindered carbon atom in a ring‐opening step.^[^ ^20^, ^26^, ^29^, ^30^ ^]^ Quantum chemical calculations (at the Def2‐TZVPPm level) were carried out to probe this and other mechanistic possibilities; no simplifications were made to the structure of **1**—to ensure and that steric (as well as electronic) influences could be fully accounted for. Despite repeated attempts, however, the geometries of the intermediates and transition states implied by a metalla‐cyclopropene mechanism could not be optimized. Alternative mechanistic possibilities were therefore considered via initial coordination of the alkyne to the formally vacant 5*p_z_* orbital of the stannylene (Scheme 5 and Figures 6, 7, 8). From an initial transition state, **TS1**, corresponding to approach of the alkyne in this fashion, the alkyne component pivots, turning in the direction of the silyl ligand to generate intermediate **I1**. The alkyne then moves further to lie parallel to the Sn─Si bond, which allows concerted addition to occur via **TS2** to yield the respective insertion products. From this generic mechanism, the stereo‐ and regioselectivity associated with specific alkynes can then be understood. *Syn*‐addition is implicit in all cases since the activation of the alkyne across the Sn─Si bond occurs in a concerted fashion. In the case of unsymmetrical alkynes, the lowest energy pathway involves alignment of the least sterically encumbered group (R') directed along the ligand framework, which then points toward the tin‐bound silyl group in **TS2**. The result is that the hypersilyl substituent ends up being attached to the least hindered carbon atom.

The specific free energy profiles for the reactions of **1** with phenylacetylene and acetylene to form **2** and **3**, respectively, are shown in Figure 6. These show that the formation of both **2** and **3** are strongly exergonic (Δ*G*° = −51.0 and −86.5 kJ mol^−1^, respectively), which is consistent with the observed irreversibility of these insertion processes. The formation of **2** and **3** is each associated with relatively low overall activation barriers (Δ*G* ^‡^ = +83.7 and +71.1 kJ mol^−1^, respectively) due to the ease with which the sterically minimal H substituent can line up against the ligand framework. For comparison, the overall activation barrier for phenylacetylene to insert in the opposite sense to form product **2′** (Figure 7) was found to be significantly higher (Δ*G* ^‡^ = +127.5 kJ mol^−1^), accounting for the selectivity observed for the room temperature reaction. Calculations of single‐point energies show that **2′** is more thermodynamically stable than **2** (ΔΔ*G*° = −6.2 kJ mol^−1^). With this in mind, a solution of **2** in toluene‐d_8_ was heated to 105 °C for 3 days, but this showed no evidence for the formation of any alternative insertion products, and instead resulted in slow decomposition of **2** with the formation of metallic tin.

The free energy profiles for the reversible reactions of **1** with trimethylsilylacetylene and 3‐hexyne were also calculated (Figure 8). For the formation of both **6** and **7**, the net free energy change accompanying the conversion of the reactants to the products is close to zero (Δ*G*° = −3.0 and +4.4 kJ mol^−1^, respectively), aligning with the experimentally determined values (Δ*G*° = −9.7 ± 5.2 and −4.0 ± 8.2 kJ mol^−1^, respectively). The slower kinetics for the formation of **7** can be accounted for by the significantly higher overall activation energy (Δ*G* ^‡^ = +123.8 kJ mol^−1^) than for **6** (Δ*G* ^‡^ = +98.0 kJ mol^−1^) due to the alignment of an ethyl group against the ligand framework compared to a proton. Insertion of trimethylsilylacetylene in the opposite sense was also shown to be implausible due to prohibitive steric crowding.

## 3 Conclusions

In conclusion, the alkynes phenylacetylene, acetylene, and 2‐butyne have been shown to insert irreversibly and selectively into the Sn─Si bond of aryl(silyl)stannylene **1** to produce products **2**, **3**, and **5**, respectively. Each of these (vinyl)stannylenes features a *syn* arrangement of the Sn and Si‐based substituents around the carbon–carbon double bond. In the products arising from unsymmetrical alkynes, the sterically more bulky group is bound to the same carbon atom as tin. Remarkably, alkyne insertion is shown to occur reversibly in the cases of 1‐phenyl‐1‐propyne, trimethylsilylacetylene, and 3‐hexyne yielding the corresponding products **4**, **6**, and **7**, respectively. The thermodynamic parameters for the formation of **6** and **7** have been determined by VT NMR spectroscopy, while the general mechanism for insertion and associated reaction profiles have been elucidated computationally. Regioselectivity in this chemistry is driven (kinetically) by the *differential* steric profiles of the alkyne substituents, with the very large steric profile of the hypersilyl group favoring its transfer to the least hindered alkyne carbon. Thermodynamically, the issue of reversibility appears to be related to the *combined* steric profiles of the two alkyne substituents such that strongly exergonic reactions are found for H/H, Ph/H, and Me/Me combinations, but close‐to‐thermoneutral insertion is seen for Me_3_Si/H and Et/Et substituents.

## 4 Experimental Section

Included here are synthetic and characterizing data for compounds **2** and **5**. Complete data for all novel compounds, representative spectra, and details of crystallographic and quantum chemical studies are included in the Supporting Information.^[^ ^33^ ^]^

**2**: Excess phenylacetylene (0.10 mL, 0.91 mmol) was added to a solution of **1** (100 mg, 0.147 mmol) in toluene (5 mL), resulting in an immediate color change from green to purple. The product was isolated as a purple powder after removal of volatiles in vacuo. Extraction into pentane (ca. 3 mL), concentration of the solution to incipient crystallization and storage at −30 °C yielded the product **2** as purple crystals suitable for X‐ray crystallography. Yield: 30 mg, 26%. Calc. for C_41_H_58_Si_4_Sn: C 62.98%, H 7.48%. Measured: C 63.57%, H 7.45%. ^1^H NMR (600 MHz, toluene‐d_8_, 298 K): *δ*_H_ 7.49 (s, 1H, C═C(H)Si), 7.26 (t, ^3^ *J*_HH_ = 7.5 Hz, 1H, *p*‐CH Ar^Mes^), 7.04 (t, ^3^ *J*_HH_ = 7.5 Hz, 2H, *m*‐CH Ph), 7.01 (d, ^3^ *J*_HH_ = 7.5 Hz, 2H, *o*‐CH Ph), 6.91 (d, ^3^ *J*_HH_ = 7.5 Hz, 2H, *m*‐CH Ar^Mes^), 6.88 (t, ^3^ *J*_HH_ = 7.5 Hz, 1H, *p*‐CH Ph), 6.76 (s, 4H, *p*‐CH Mes), 2.46 (s, 6H, CH_3_ Mes), 2.17 (s, 6H, Me Mes), 1.79 (s, 6H, Me Mes), 0.17 (s, 27H, Si(Si(CH_3_)_3_)_3_). ^13^C{^1^H} NMR (151 MHz, toluene‐d_8_, 298 K): *δ*_C_ 218.3, 179.7, 153.1, 149.2, 146.1, 137.2, 136.8, 130.0, 129.9, 129.5, 127.9, 127.7, 126.9, 126.0, 21.5, 21.2, 20.9, 1.4. ^29^Si{^1^H} NMR (119 MHz, toluene‐d_8_, 298 K): *δ*_Si_ − 12.9, −86.9 (assigned by ^29^Si/^1^H HMBC). ^119^Sn{^1^H} NMR (187 MHz, toluene‐d_8_, 298 K): *δ*_Sn_ 1690.

**5**: Excess 2‐butyne (0.20 mL, 2.6 mmol) was added to a solution of **1** (100 mg, 0.147 mmol) in toluene (5 mL), and the reaction mixture stirred at room temperature for 16 hours, during which time the color of the solution changed to purple. Volatiles were removed in vacuo yielding the crude product **5** as a purple powder. Storage of a concentrated toluene solution of this product at − 30 °C for an extended period yielded a small number of crystals suitable for X‐ray crystallography. Yield: 23 mg, 21%. Calc. for C_37_H_58_Si_4_Sn: C 60.55%, H 7.97%, Si 15.31%, Sn 16.17%. Measured: C 61.17%, H 8.12%. ^1^H NMR (400 MHz, C_6_D_6_, 298 K): *δ*_H_ 7.34 (t, ^3^ *J*_HH_ = 7.5 Hz, 1H, *p*‐CH Ar^Mes^), 7.11 (d, ^3^ *J*_HH_ = 7.5 Hz, 2H, *m*‐CH Ar^Mes^), 6.77 (s, 4H, *m*‐CH Mes), 2.53 (q, ^3^ *J*_HH_ = 1.0 Hz, 3H, SnC(CH_3_) = C), 2.43 − 2.23 (br. s, 12H, *o*‐CH_3_ Mes), 2.14 (s, 6H, *p*‐CH_3_ Mes), 1.87 (q, ^3^ *J*_HH_ = 1.0 Hz, 3H, C═C(CH_3_)Si), 0.17 (s, 27H, Si(Si(CH_3_)_3_)_3_). ^13^C{^1^H} NMR (101 MHz, C_6_D_6_, 298 K): *δ*_C_ 210.2, 182.0, 153.5, 146.1, 137.2, 137.2, 129.8, 129.5, 128.2, 127.9, 27.6, 26.7, 21.2, 2.8. ^29^Si{^1^H} NMR (80 MHz, C_6_D_6_, 298 K): *δ*_Si_ − 14.2, −72.9 (assigned by ^29^Si/^1^H HMBC). ^119^Sn{^1^H} NMR (150 MHz, C_6_D_6_, 298 K): *δ*_Sn_ 1754.

## Supporting Information

The authors have cited additional references within the Supporting Information.^[^ ^34^, ^35^, ^36^, ^37^, ^38^, ^39^, ^40^, ^41^, ^42^, ^43^, ^44^, ^45^, ^46^, ^47^, ^48^, ^49^, ^50^, ^51^, ^52^, ^53^, ^54^, ^55^ ^]^

## Conflict of Interest

The authors declare no conflict of interest.

## Supporting information



Supporting Information

Supporting Information

Supporting Information

Supporting Information

Supporting Information

## Data Availability

The data that support the findings of this study are available in the supplementary material of this article.
