# Supplementary material for: Reversible and Irreversible Regioselective Alkyne Insertion into a Silyl‐Substituted Stannylene
Source: Chemistry. 2025 Jul 28;31(45):e02103. doi: 10.1002/chem.202502103 (PMC12351429; doi:10.1002/chem.202502103)
Supplement: Supplementary file 1 — Supporting Information [file CHEM-31-e02103-s004.pdf]

Chemistry - A European Journal

Supporting Information

Reversible and Irreversible Regioselective Insertion of Alkynes into a Silyl-substituted Stannylene

Aidan J. Murray, Lewis L. Wales, Agamemnon E. Crumpton, Maximilian Dietz, Mathias A. Ellwanger, Andreas Heilmann, Job J. C. Struijs and Simon Aldridge*

Supporting Information:

1. General Considerations S2
2. Preparation of Known Compounds/Precursors S3
3. Syntheses of Novel Compounds S4
4. X-ray Crystallographic Details S7
5. Variable Temperature Experiments for Alkyne Uptake S9
6. NMR Spectra of Novel Compounds S11
7. Computational Details S17
8. References S29

1. General Considerations

All manipulations were performed using standard Schlenk line or dry-box techniques under an argon or nitrogen atmosphere. Solvents were degassed by sparging with argon and dried by passing through a column of the appropriate drying agent, then stored over a potassium mirror in Teflon valve ampoules. NMR spectra were measured in benzene-d_6 or toluene-d_8 solvent, which was pre-dried over CaH_2, with the solvent then being distilled under reduced pressure and stored under argon in Teflon valve ampoules. NMR samples were prepared under argon in 5 mm Wilmad 507-PP tubes fitted with J. Young Telfon valves. NMR spectra were measured on a Bruker Avance III HD nanobay 400 MHz NMR spectrometer, a Bruker Avance III 500 MHz NMR spectrometer, or a Bruker NEO 600 MHz NMR spectrometer equipped with a broadband helium cryoprobe. All ^{13}C NMR measurements were performed with proton decoupling. ^{1}H and ^{13}C NMR spectra were referenced internally to residual protio-solvent (^{1}H) or solvent (^{13}C) resonances and are reported relative to tetramethylsilane (δ = 0 ppm). $^{29}Si\{^{1}H\}$ NMR spectra are referenced externally with respect to tetramethylsilane. $^{119}Sn\{^{1}H\}$ NMR spectra were referenced externally with respect to $SnMe_4$ in C_6D_6. Chemical shifts are quoted in δ (ppm) and coupling constants in Hz. 2D NMR techniques (COSY, HSQC, and HMBC) were used to aid in assignment of spectra. Elemental analyses were carried out by London Metropolitan University. Commercially available samples of phenylacetylene, 1-phenylpropyne, 2-butyne, trimethylsilyl-acetylene and 3-hexyne were purified by vacuum transfer and stored over freshly activated 3 Å molecular sieves. Acetylene was dried over P_2O_5. All other reagents were commercially sourced and used as received.

2. Preparation of Known Compounds/Precursors

$Si(SiMe_3)_4$,[S1] $Sn[N(SiMe_3)_2]_2$,[S2] $Ar^{Mes}SnN(SiMe_3)_2$,[S2] and $Ar^{Mes}SnSi(SiMe_3)_3$[S3] (**1**) were prepared according to existing procedures.

Ar^{Mes}**I**: (Adapted from the procedure by Du et al.)[S4] To a suspension of magnesium shavings (6.40 g, 263 mmol) in THF (60 mL) in a three-necked round-bottom flask fitted with a reflux condenser was added dropwise a solution of mesitylbromide (34.0 mL, 226 mmol) in THF (100 mL). After the addition was finished, the reaction was heated at reflux for 4 h before being cooled to room temperature. A solution of dichlorobenzene (10.0 mL, 89 mmol) in THF (100 mL) was cooled to −78 °C and *n*-butyllithium (58 mL of a 1.6 M solution, 93 mmol) added slowly with rapid stirring. After the addition was complete, the reaction mixture was stirred at −78 °C for 2 h before dropwise addition of the filtered Grignard solution at −78 °C. The solution was then allowed to warm to ambient temperature and stirred for 12 h. The reaction mixture was then heated to reflux for 2 h before being cooled to 0 °C. Once at 0 °C, a solution of iodine (33.4 g, 131 mmol) in THF (150 mL) was added slowly to the reaction mixture. After warming to room temperature and stirring overnight, any excess iodine was quenched with aqueous sodium sulphite solution. With the organic and aqueous layers separated, the aqueous layer was extracted with diethyl ether (3 x 20 mL). The combined organic fractions were then dried over magnesium sulphate, and volatiles removed *in vacuo* to yield the crude product. The spectroscopically pure product was isolated by washing the crude product with hot ethanol to yield Ar^{Mes}**I** as a fluffy white solid. Yield 19.19 g, 47%. NMR spectra matched those reported by Du *et al*.[S4]

Ar^{Mes}Li: To a suspension of Ar^{Mes}**I** (7.00 g, 16 mmol) in hexane (100 mL) at −78 °C was added dropwise *n*-butyllithium (11 mL of 1.6 M solution, 18 mmol) with rapid stirring. The reaction mixture was allowed to warm to room temperature and stirred for 12 h. After filtration, the residue was washed with pentane at room temperature (2 x 15 mL), to yield the spectroscopically pure product Ar^{Mes}Li as a white solid. An additional crop of product could be obtained by removing the volatiles from the filtrate *in vacuo* and washing the residue with pentane (3 x 20 mL) at room temperature. Overall yield: 4.20 g, 82 %. NMR spectra matched those reported by Schulz *et al*.[S5]

$(THF)_2KSi(SiMe_3)_3$: (Adapted from a procedure by Marchner *et al.*)[S6] Tetrakis(trimethylsilyl)silane (3.55 g, 11.1 mmol) and potassium *tert*-butoxide (1.31 g, 11.7 mmol) were combined as solids and dissolved in THF (40 mL) at room temperature. After stirring overnight, the solvent was removed *in vacuo* and the resulting solid was extracted into pentane (50 mL) and filtered. Concentration of the solution to around two thirds of its original volume and storage at −30 °C afforded a crop of crystals of the product. Yield 3.25 g, 68%. NMR spectra matched those reported by Marschner *et al*.[S6]

3. Syntheses of Novel Compounds

2: Excess phenylacetylene (0.10 mL, 0.91 mmol) was added to a solution of **1** (100 mg, 0.147 mmol) in toluene (5 mL), resulting in an immediate colour change from green to purple. The product was isolated as a purple powder after removal of volatiles *in vacuo*. Extraction into pentane, concentration of the solution to incipient crystallisation and storage at −30 °C yielded the product **2** as purple crystals suitable for X-ray crystallography. Yield: 30 mg, 26 %.

Calc. for $C_{41}H_{58}Si_4Sn$: C 62.98%, H 7.48%. Measured: C 63.57%, H 7.45%.

^{1}H NMR (600 MHz, toluene-d$_8$, 298 K): δ_H 7.49 (s, 1H, C=C(H)Si), 7.26 (t, $^{3}J_{HH}$ = 7.5 Hz, 1H, *p*-CH Ar^{Mes}), 7.04 (t, $^{3}J_{HH}$ = 7.5 Hz, 2H, *m*-CH Ph), 7.01 (d, $^{3}J_{HH}$ = 7.5 Hz, 2H, *o*-CH Ph), 6.91 (d, $^{3}J_{HH}$ = 7.5 Hz, 2H, *m*-CH Ar^{Mes}), 6.88 (t, $^{3}J_{HH}$ = 7.5 Hz, 1H, *p*-CH Ph), 6.76 (s, 4H, *p*-CH Mes), 2.46 (s, 6H, CH_3 Mes), 2.17 (s, 6H, Me Mes), 1.79 (s, 6H, Me Mes), 0.17 (s, 27H, $Si(Si(CH_3)_3)_3$).

^{13}C{^{1}H} NMR (151 MHz, toluene-d$_8$, 298 K): δ_C 218.3, 179.7, 153.1, 149.2, 146.1, 137.2, 136.8, 130.0, 129.9, 129.5, 127.9, 127.7, 126.9, 126.0, 21.5, 21.2, 20.9, 1.4.

^{29}Si{^{1}H} NMR (119 MHz, toluene-d$_8$, 298 K): δ_{Si} −12.9, −87.2 (assigned by ^{29}Si/^{1}H HMBC).

^{119}Sn{^{1}H} NMR (187 MHz, toluene-d$_8$, 298 K): δ_{Sn} 1690.

3: A solution of **1** (20 mg, 0.0287 mmol) in C_6D_6 (0.40 mL) was degassed *via* three freeze-pump-thaw cycles before the atmosphere was charged with acetylene (ca. 1 bar). The colour of the reaction mixture turned from green to red upon vigorous shaking, from which compound **3** could be spectroscopically characterised *in situ*. Despite multiple, repeated attempts, the high solubility of **3** in hydrocarbon solvents meant that single crystals could not be obtained.

^{1}H NMR (600 MHz, C_6D_6, 298 K): δ_H 9.49 (d, $^{3}J_{HH}$ = 16.7 Hz, 1H, Sn–C(H)=C), 8.06 (d, $^{3}J_{HH}$ = 16.7 Hz, 1H, C=C(H)Si), 7.33 (t, $^{3}J_{HH}$ = 7.5 Hz, 1H, *p*-CH Ar^{Mes}), 7.08 (d, $^{3}J_{HH}$ = 7.5 Hz, 2H, *m*-CH Ar^{Mes}), 6.77 (s, 4H, *m*-CH Mes), 2.27 (s, 12H, *o*-Me Mes), 2.16 (s, 6H, *p*-Me Mes), 0.15 (s, $Si(Si(CH_3)_3)_3$, 27H).

^{13}C{^{1}H} NMR (151 MHz, C_6D_6, 298 K): δ_C 202.0, 177.5, 146.7, 146.2, 137.4, 136.2, 135.8, 129.4, 128.5, 128.4, 21.3, 21.2, 1.2.

^{29}Si{^{1}H} NMR (119 MHz, C_6D_6, 298 K): δ_{Si} −14.1, −85.9 (assigned by ^{29}Si/^{1}H HMBC).

^{119}Sn{^{1}H} NMR (187 MHz, C_6D_6, 298 K): not found.

4: Excess 1-phenylprop-1-yne (0.20 mL, 1.60 mmol) was added to a solution of **1** (150 mg, 0.221 mmol) in toluene (5 mL). The reaction mixture was stirred at room temperature for 16 h, during which time the colour of the solution changed to purple. Volatiles were removed *in vacuo*, yielding the crude product as a purple powder. Subsequent extraction into pentane, concentration to incipient crystallisation, and

storage at −30 °C yielded purple crystals of the product **4**. Crystals suitable for X-ray crystallography were obtained from slow evaporation of a pentane solution at room temperature. Yield: 47 mg, 27 %.

^{1}H NMR (400 MHz, C_6D_6, 298 K): δ_H 7.22 (t, $^{3}J_{HH}$ = 7.5 Hz, 1H, p-CH Ar^{Mes}), 7.15 (t, $^{3}J_{HH}$ = 7.6 Hz, 2H, *m*-CH Ph), 6.95 (d, $^{3}J_{HH}$ = 7.5 Hz, 2H, *m*-CH Ar^{Mes}), 6.87 (t, $^{3}J_{HH}$ = 7.6 Hz, 1H, *p*-CH Ph), 6.85 (s, 2H, *m*-CH Mes), 6.76 (s, 2H, *m*-CH Mes), 6.60 (d, $^{3}J_{HH}$ = 7.5 Hz, 2H, *m*-CH Mes), 2.45 (s, 6H, CH_3 Mes), 2.20 (s, 6H, CH_3 Mes), 1.84 (s, 6H, CH_3 Mes), 1.72 (s, 3H, C=C(CH_3)Si), 0.21 (s, 27H, Si(Si(CH_3)$_3$)$_3$).

^{13}C{^{1}H} NMR (101 MHz, C_6D_6, 298 K): δ_C 216.2, 180.3, 156.2, 150.7, 146.3, 137.9, 137.8, 137.5, 137.3, 134.5, 131.9, 130.1, 129.7, 129.6, 127.5, 124.7, 30.3, 21.8, 21.7, 21.3, 2.3.

^{29}Si{^{1}H} NMR (80 MHz, C_6D_6, 298 K): δ_{Si} −13.7, −73.7 (assigned by ^{29}Si/^{1}H HMBC).

^{119}Sn{^{1}H} NMR (150 MHz, C_6D_6, 298 K): δ_{Sn} 1722.

5: Excess 2-butyne (0.20 mL, 2.6 mmol) was added to a solution of **1** (100 mg, 0.147 mmol) in toluene (5 mL), and the reaction mixture stirred at room temperature for 16 h, during which time the colour of the solution changed to purple. Volatiles were removed *in vacuo* yielding the crude product **5** as a purple powder. Storage of a concentrated toluene solution of this product at −30 °C for an extended period yielded a small number of crystals suitable for X-ray crystallography. Yield: 23 mg, 21 %.

Calc. for $C_{37}H_{58}Si_4Sn$: C 60.55%, H 7.97%, Si 15.31%, Sn 16.17%. Measured: C 61.17%, H 8.12%.

^{1}H NMR (400 MHz, C_6D_6, 298 K): δ_H 7.34 (t, $^{3}J_{HH}$ = 7.5 Hz, 1H, *p*-CH Ar^{Mes}), 7.11 (d, $^{3}J_{HH}$ = 7.5 Hz, 2H, *m*-CH Ar^{Mes}), 6.77 (s, 4H, *m*-CH Mes), 2.53 (q, $^{3}J_{HH}$ = 1.0 Hz, 3H, SnC(CH_3)=C), 2.43 − 2.23 (br. s, 12H, *o*-CH_3 Mes), 2.14 (s, 6H, *p*-CH_3 Mes), 1.87 (q, $^{3}J_{HH}$ = 1.0 Hz, 3H, C=C(CH_3)Si), 0.17 (s, 27H, Si(Si(CH_3)$_3$)$_3$).

^{13}C{^{1}H} NMR (101 MHz, C_6D_6, 298 K): δ_C 210.2, 182.0, 153.5, 146.1, 137.2, 137.2, 129.8, 129.5, 128.2, 127.9, 27.6, 26.7, 21.2, 2.8.

^{29}Si{^{1}H} NMR (80 MHz, C_6D_6, 298 K): δ_{Si} −14.2, −72.9 (assigned by ^{29}Si/^{1}H HMBC).

^{119}Sn{^{1}H} NMR (150 MHz, C_6D_6, 298 K): δ_{Sn} 1754.

6: To a Schlenk flask containing **1** (150 mg, 0.22 mmol) was added excess neat trimethylsilylacetylene (10 mL, 72 mmol), with immediate formation of a blue solution. The reaction mixture was stirred at room temperature for 4 h before concentration until incipient crystallisation. Removal of the volatiles *in vacuo* yielded the product **6**. Dissolution of this product in C_6D_6 immediately leads to the formation of equilibrium mixture containing the products **6**, together with **1** and trimethylsilylacetylene, from which compound **6** was spectroscopically characterised *in situ*. Crystals suitable for X-ray crystallography were obtained from slow evaporation of a solution of **1** in trimethylsilylacetylene. Yield: 102 mg, 59 %.

^{1}H NMR (400 MHz, C_6D_6, 298 K): δ_H 9.16 (s, 1H, C=C(H)Si), 7.31 (t, $^{3}J_{HH}$ = 7.5 Hz, 1H, *p*-CH Ar^{Mes}), 7.10 (d, $^{3}J_{HH}$ = 7.5 Hz, 2H, *m*-CH Ar^{Mes}), 6.79 (s, 2H, *m*-CH Mes), 6.76 (s, 2H, *m*-CH Mes), 2.44 (s, 6H, CH_3 Mes), 2.32 (s, 6H, CH_3 Mes), 2.12 (s, 6H, CH_3 Mes), 0.20 (s, 9H, $CSi(CH_3)_3$), 0.11 (s, 27H, $Si(Si(CH_3)_3)_3$.

$^{13}C\{^{1}H\}$ NMR (101 MHz, C_6D_6, 298 K): δ_C 223.1, 182.0, 165.3, 146.0, 137.3, 136.9, 136.6, 135.6, 130.1, 129.9, 129.8, 128.5, 22.0, 21.1, 2.1, 1.5.

$^{29}Si\{^{1}H\}$ NMR (80 MHz, C_6D_6, 298 K): δ_{Si} 0.0, −12.9, −85.0 (assigned by $^{29}Si/^{1}H$ HMBC).

$^{119}Sn\{^{1}H\}$ NMR (150 MHz, C_6D_6, 298 K): δ_{Sn} 1780.

7: To an NMR tube containing **1** (30 mg, 0.044 mmol) was added excess neat 3-hexyne (1 mL, 8.8 mmol). This mixture was inverted several times and allowed to equilibrate at room temperature for 16 h, over which time a colour change from green to purple was observed. The 3-hexyne was removed *in vacuo* to yield the crude product **7**, whereupon dissolution into C_6D_6 allowed characterisation of product **7** by *in situ* ^{1}H NMR spectroscopy before subsequent decomposition into **1** and 3-hexyne.

^{1}H NMR (400 MHz, C_6D_6, 298 K): δ_H 7.33 (t, $^{3}J_{HH}$ = 7.5 Hz, 1H, *p*-CH Ar^{Mes}), 7.09 (d, $^{3}J_{HH}$ = 7.5 Hz, 2H, *m*-CH Ar^{Mes}), 6.78 (s, 4H, *m*-CH Mes), 3.23 (q, $^{3}J_{HH}$ = 7.4 Hz, 2H, CCH_2CH_3), 2.16-2.46 (br. s, 12H, *o*-CH_3 Mes), 2.31 (q, $^{3}J_{HH}$ = 7.4 Hz, 2H, CCH_2CH_3), 2.15 (s, 6H, *p*-CH_3 Mes), 0.86 (t, $^{3}J_{HH}$ = 7.4 Hz, 3H, CCH_2CH_3), 0.82 (t, $^{3}J_{HH}$ = 7.4 Hz, 3H, CCH_2CH_3), 0.20 (s, 27H, $Si(Si(CH_3)_3)_3$).

$^{119}Sn\{^{1}H\}$ NMR (150 MHz, 3-hexyne, 298 K): δ_H 1694.

4. X-ray Crystallographic Details

Single-crystal X-ray diffraction data were collected on an Oxford Diffraction/Agilent SuperNova diffractometer equipped with a 135 mm Atlas CCD area detector. Crystals were picked under Pantone-N oil and mounted on MiTeGen Micromount loops whilst being quench cooled using an Oxford Cryosystems open flow N_2 cooling device.[S7] All data were collected at 150 K using mirror monochromated Cu Kα radiation (λ = 1.54184 Å). Once collected, the data were processed using the CrysAlisPro package. Structures were solved *ab initio* from the integrated intensities using SHELXT and refined on F^2 using SHELXL with the graphical interface OLEX2. [S8–S10]

Table S1. X-ray crystallographic details for compounds **2** - **6**.

	**2**	**4**	**5**	**6**
**Formula**	$C_{41}H_{58}Si_4Sn$	$C_{42}H_{60}Si_4Sn$	$C_{37}H_{58}Si_4Sn$	$C_{39}H_{64}Si_5Sn$
**Fw (g mol^{-1})**	781.97	795.99	733.92	778.01
**Crystal system**	Triclinic	Monoclinic	Monoclinic	Triclinic
**Space group**	P -1	C 2/c	P 1 21/m 1	P -1
**a (Å)**	12.7291(5)	27.8616(3)	10.2105(1)	11.1688(7)
**b (Å)**	12.9103(4)	27.8832(3)	16.3926(1)	12.8011(8)
**c (Å)**	13.8581(3)	44.8177(7)	12.2821(1)	16.1331(7)
**α (°)**	86.054(2)	90	90	89.721(4)
**ß (°)**	89.117(2)	99.3440(10)	104.219(1)	87.559(4)
**γ (°)**	67.984(3)	90	90	71.004(5)
**V (Å^{-3})**	2106.19(12)	34355.6(8)	1992.76(3)	2178.9(2)
**Z**	2	32	2	2
**ρ_{calc} (g cm^{-3})**	1.233	1.231	1.223	1.186
**Radiation, λ (Å)**	1.54184	1.54184	1.54184	1.54184
**Absorption**	Gaussian	Gaussian	Gaussian	Gaussian
**μ (mm^{-1})**	6.097	5.989	6.408	6.141
**Reflections collected**	28157	132993	37303	15964
**Independent reflections**	8711	31387	4288	15964
**$R_{(int)}$**	0.0378	0.0907	0.0452	n/a
**Parameters**	430	2151	215	450
**R1 (I>2σ(I))**	0.0280	0.0732	0.0270	0.0510
**wR2 (all data)**	0.0694	0.1920	0.0751	0.1319
**GooF**	1.040	1.042	1.081	0.923
**T (K)**	149.98(10)	100(2)	150.01(15)	150.01(10)
**CCDC ref**	2465672	2465673	2465671	2465670

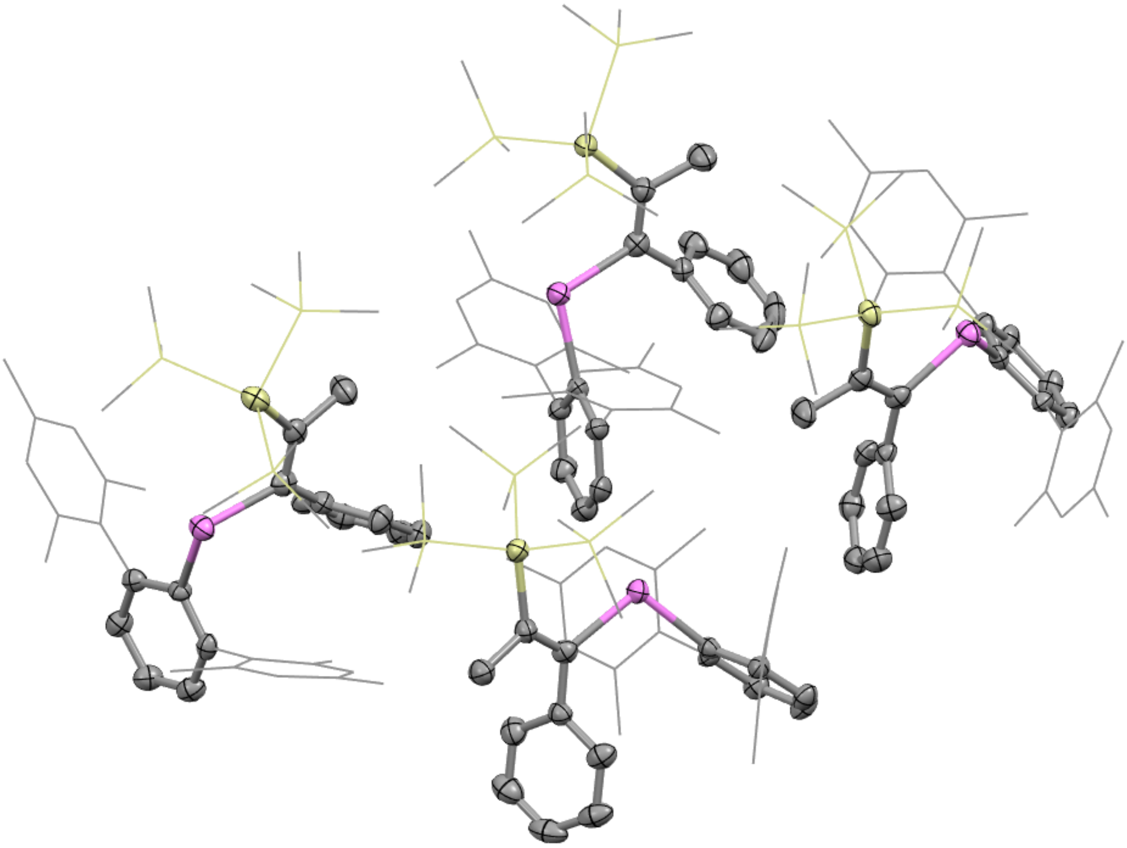

Figure S1. The asymmetric unit of the crystal structure of product **4** as determined by X-ray crystallography. Thermal ellipsoids are set at the 30% probability level. Hydrogen atoms are omitted for clarity. The Mes and $SiMe_3$ groups are depicted in wireframe for clarity. Key bond lengths (Å), angles (°) and torsions (°) are summarised in Table S2.

Table S2. Key bond lengths (Å), angles (°) and torsions (°) for the four molecules in the asymmetric unit of **4**.

	**4a**	**4b**	**4c**	**4d**	mean
C1−Sn1 (Å)	2.204(6)	2.214(7)	2.223(7)	2.215(7)	2.214(3)
Sn1−C2 (Å)	2.188(7)	2.202(8)	2.204(8)	2.193(7)	2.197(4)
C2−C3 (Å)	1.36(1)	1.35(1)	1.35(1)	1.36(1)	1.355(5)
C2−C4 (Å)	1.49(1)	1.48(1)	1.46(1)	1.48(1)	1.48(4)
C3−C5 (Å)	1.53(1)	1.54(1)	1.53(1)	1.52(1)	1.53(4)
C3−Si1 (Å)	1.933(8)	1.924(8)	1.934(7)	1.924(8)	1.929(4)
C1−Sn1−C2 (°)	105.5(2)	105.8(3)	105.7(3)	105.4(2)	105.6(1)
Sn1−C2−C3−Si1 (°)	13.0(9)	9(1)	13(1)	11.6(9)	11.7(5)

5. Variable Temperature Experiments for Alkyne Uptake

Reaction mixtures were constituted in NMR tubes fitted with J-Young valves by adding trimethylsilylacetylene (10 µL, 0.0722 mmol) to a solution of **1** (15 mg, 0.0221 mmol) in C_6D_6 (0.5 mL) or 3-hexyne (16 µL, 0.141 mmol) to a solution of **1** (24 mL, 0.0353 mmol) in C_6D_6 (1 mL). The reaction mixture in each case was then allowed to equilibrate by leaving for 16 h at ambient temperature. The samples were transferred to the NMR spectrometer and monitored by *in situ* ^{1}H NMR spectroscopy at each temperature until no change was seen in the ratio of intensities between the product and reactant resonances. The ^{1}H NMR signals corresponding to the *meta*-protons of the central aryl ring of the terphenyl group belonging to stannylene **1** (δ_H = 7.05) and the products (**6**: δ_H = 7.10 ppm; **7**: δ_H = 7.09 ppm) were integrated to determine the mole fraction of product at each temperature, from which the equilibrium constant could be calculated according to Equation S1 (Tables S3 and S5).

$$Equation\ S1: K_{eq} = \frac{[product]}{[\mathbf{1}][alkyne]}$$

With the values of K_{eq} obtained at six different temperatures the Van't Hoff equation (Equation S2) was used to graphically determine the thermodynamic parameters of the reaction (Figures 5 and S2).

$$Equation\ S2: lnK_{eq} = -\frac{\Delta H}{RT} + \frac{\Delta S}{R}$$

Graphical data is detailed below:

Table S3. Raw data for **1** + trimethylsilylacetylene

T (K)	1/T	mol fraction	lnK
308	0.00325	0.759	3.27793
313	0.00319	0.727	3.1009
318	0.00314	0.684	2.88051
323	0.00310	0.638	2.663
328	0.00305	0.531	2.19154
333	0.00300	0.503	2.07205

Table S4. Graphical fit relating to the van't Hoff plot for **1** + trimethylsilylacetylene (Figure 5).

y = (-13.6944) + (5249.93)x	
Adj. R-Square	0.96267
Intercept	-13.6944 ± 1.4385
Slope	5249.92581 ± 460.548

Table S5. Raw data for **1** + 3-hexyne:

T (K)	1/T	mol fraction	lnK
318	0.00314	0.215	0.72225
323	0.00310	0.150	0.2609
328	0.00305	0.140	0.17609
333	0.00300	0.142	0.19452
338	0.00296	0.097	-0.24447
343	0.00292	0.079	-0.47443

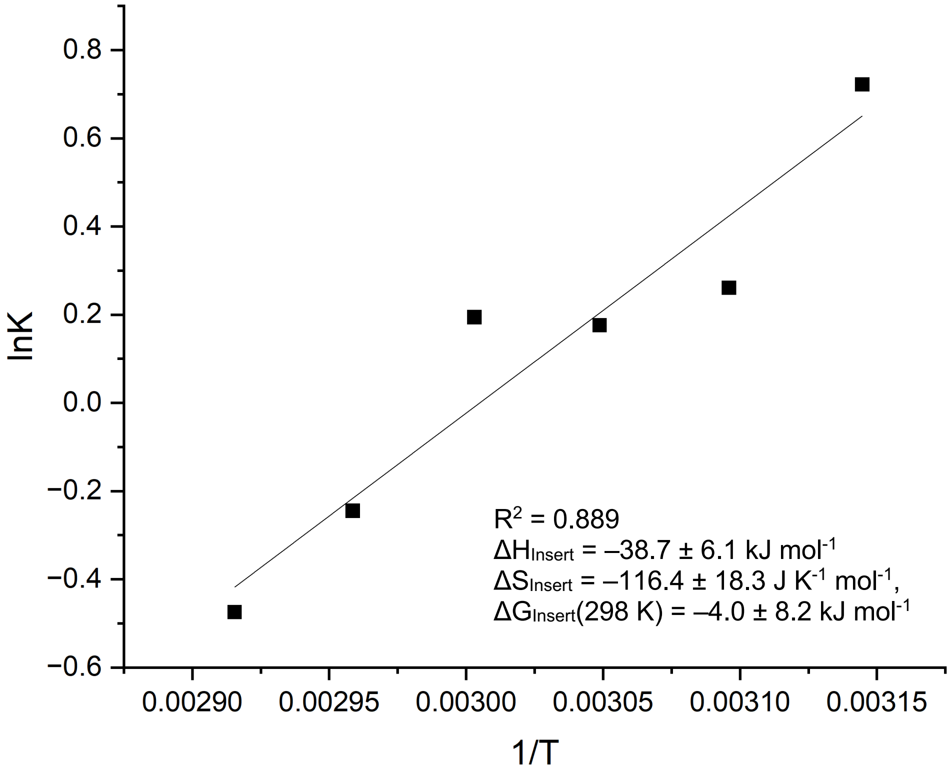

Figure S2. Van't Hoff plot and calculated thermodynamic parameters for the reaction between **1** and 3-hexyne.

Table S6. Graphical fit relating to the van't Hoff plot for **1** + 3-hexyne (Figure S2).

y = (-14.0004) + (4658.98)x	
Adj. R-Square	0.88863
Intercept	-14.00037 ± 2.2066
Slope	4658.97785 ± 728.5512

6. NMR Spectra of Novel Compounds

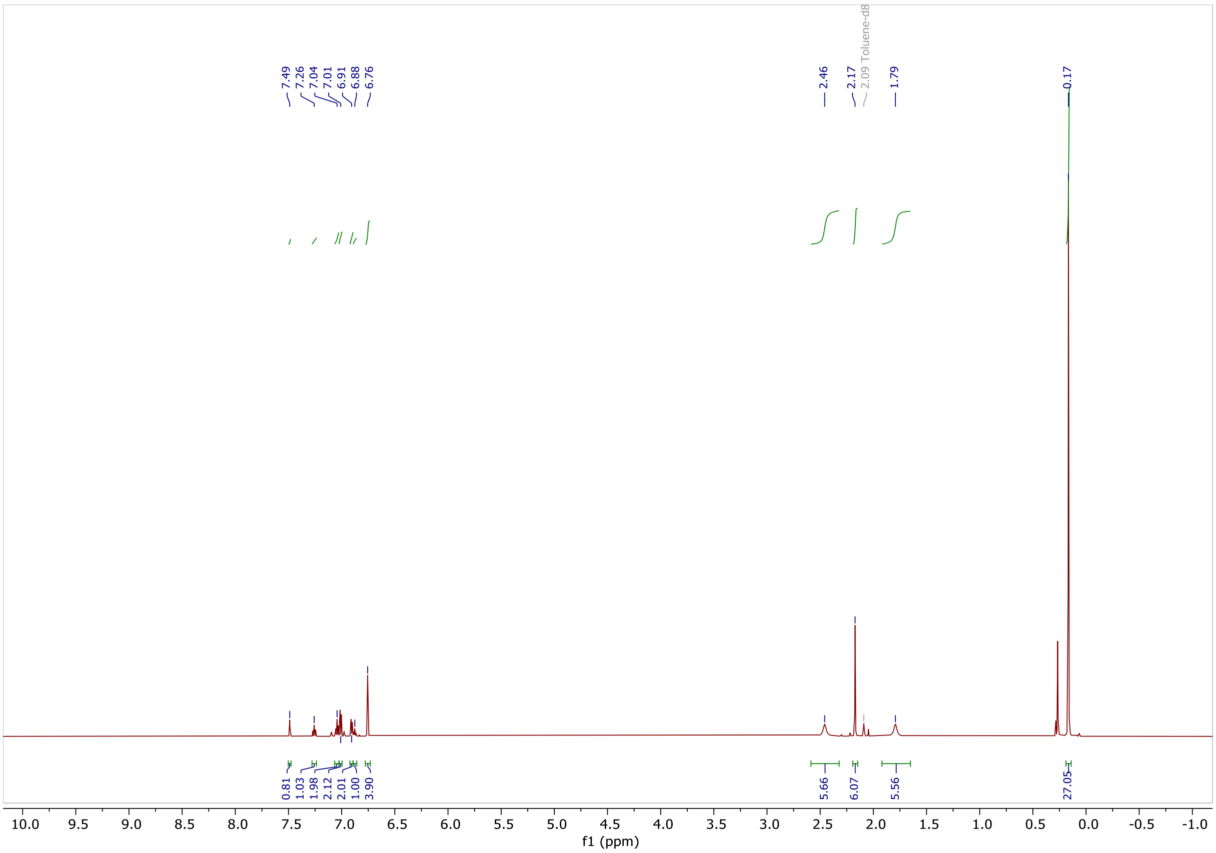

Figure S3. ^{1}H NMR spectrum of **2** in toluene-d_8 at 298 K.

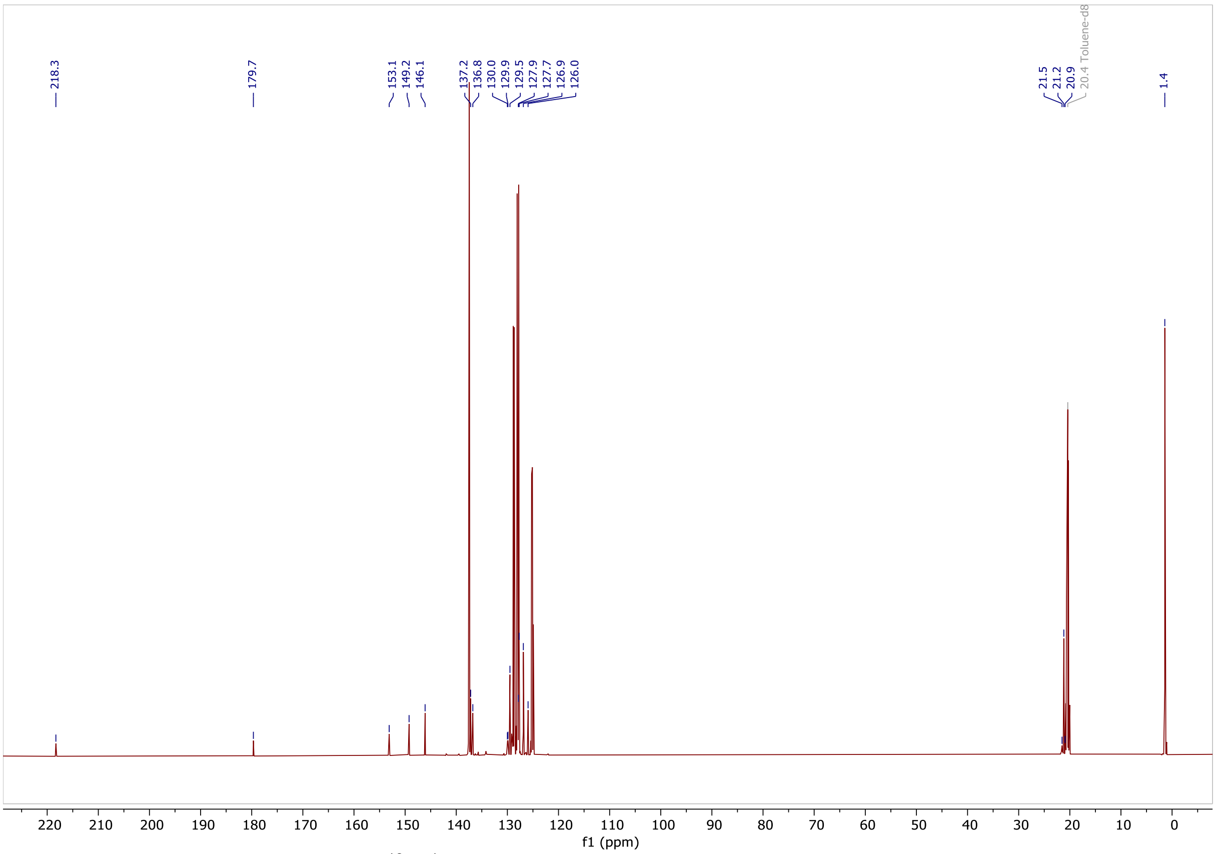

Figure S4. $^{13}C\{^{1}H\}$ NMR spectrum of **2** in in toluene-d_8 at 298 K.

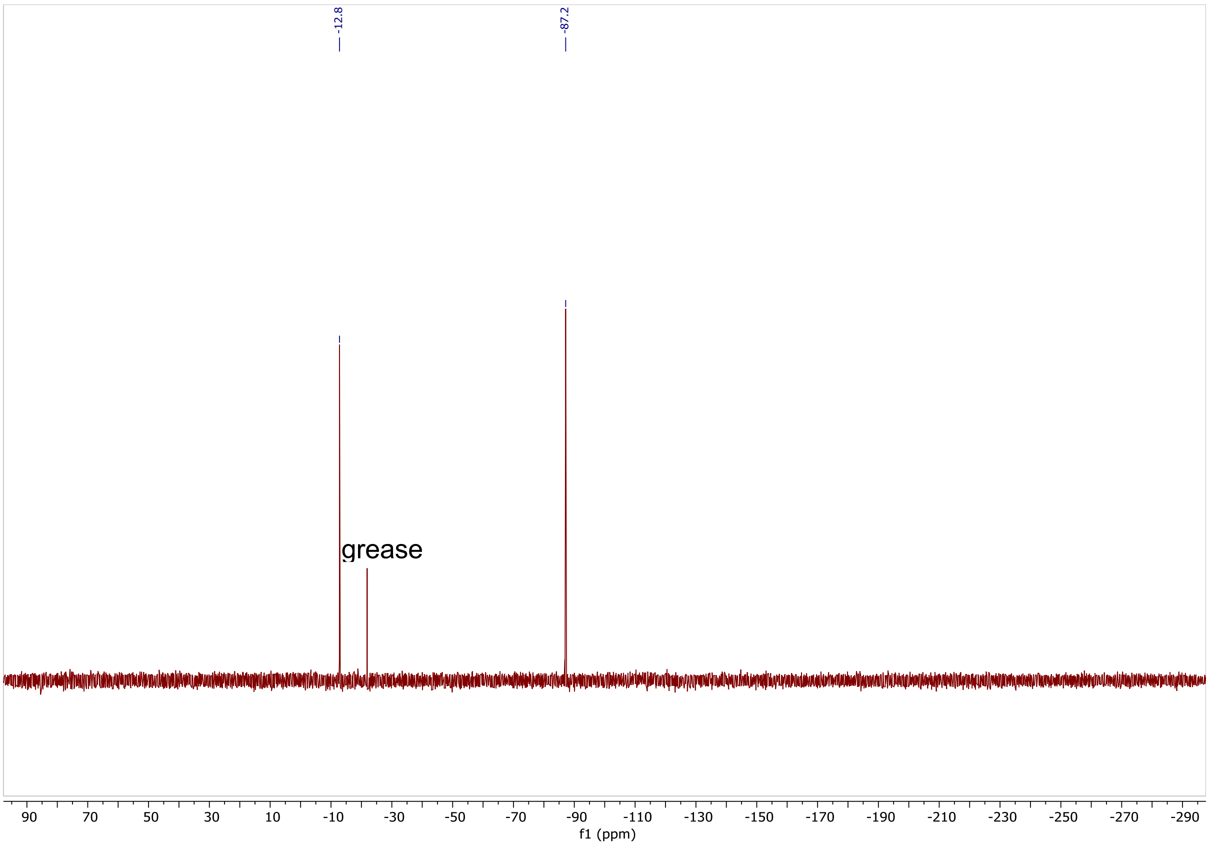

Figure S5. ^{29}Si NMR spectrum of **2** in toluene-d_8 at 298 K.

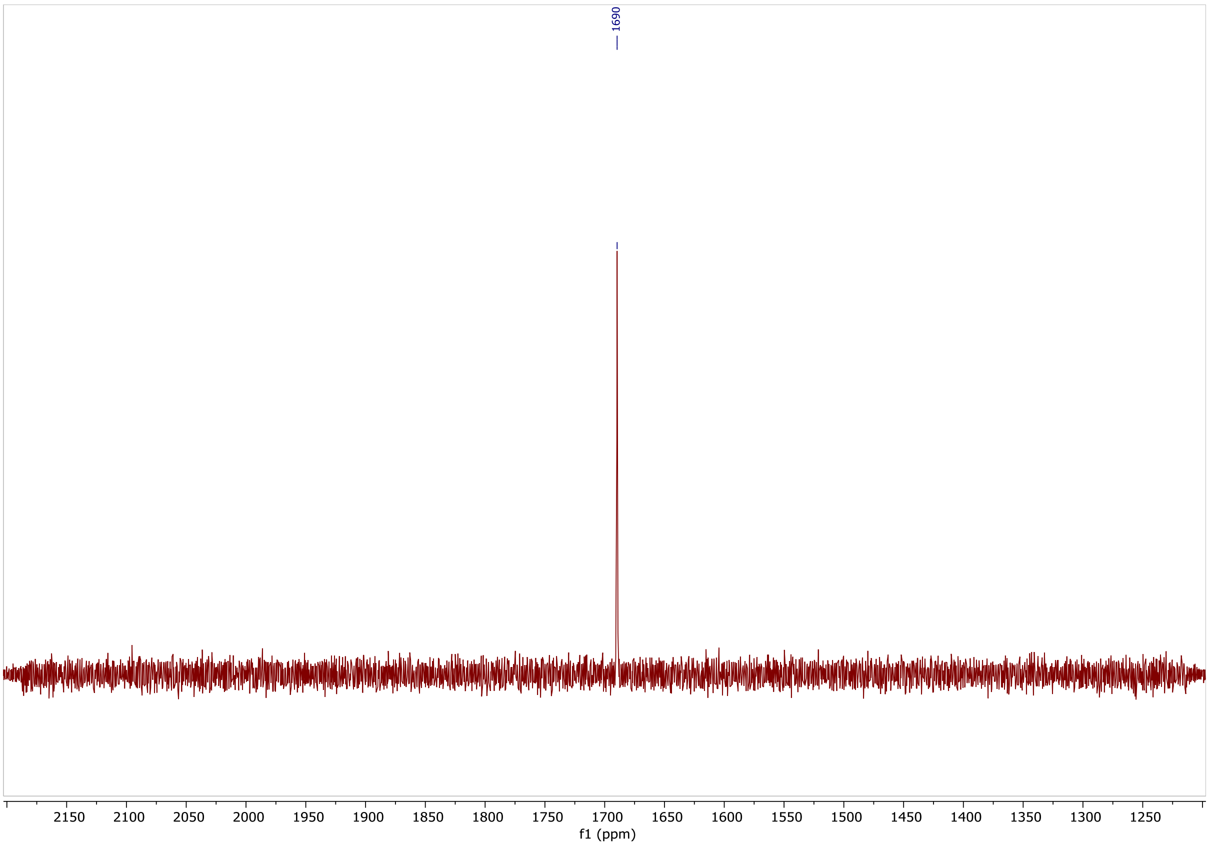

Figure S6. ^{119}Sn NMR spectrum of **2** in toluene-d_8 at 298 K.

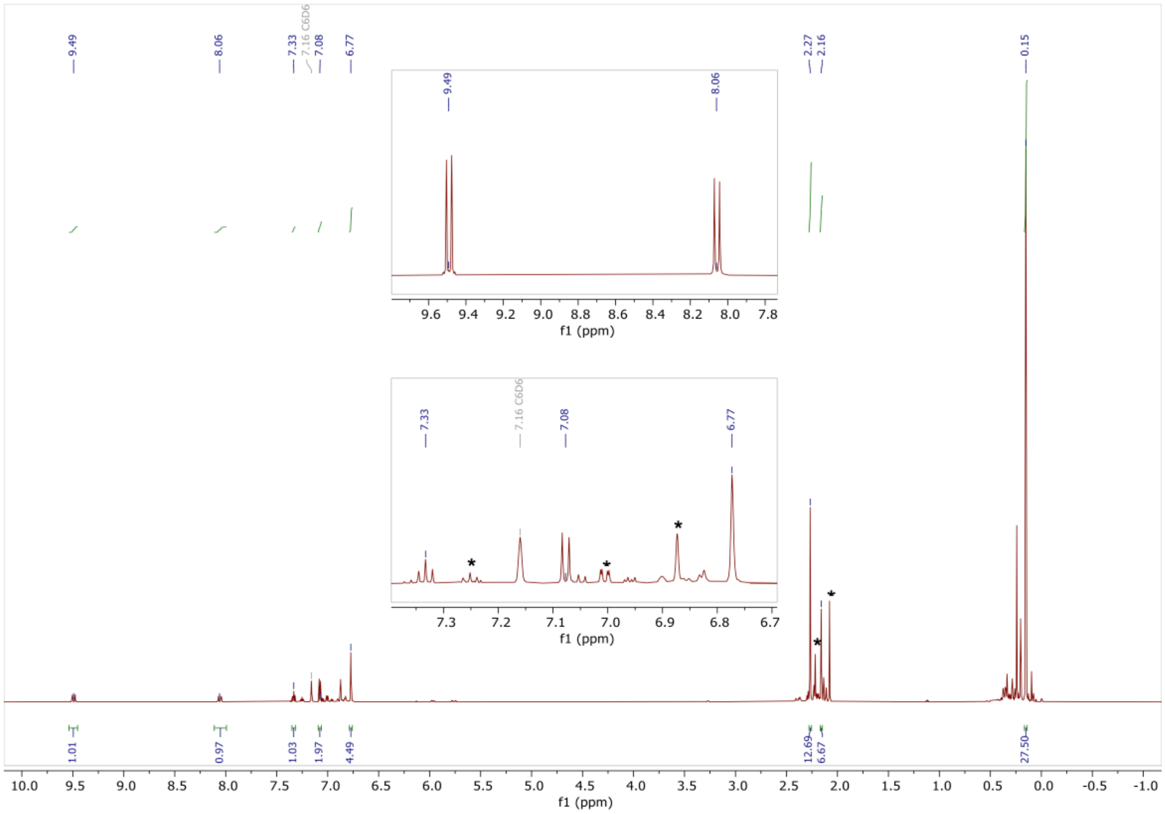

Figure S7. ^{1}H NMR spectrum of **3** in C_6D_6 at 298 K. Insets show the roofed doublets and aromatic region. Peaks belonging to the main impurity $Ar^{Mes}H$ are marked *.

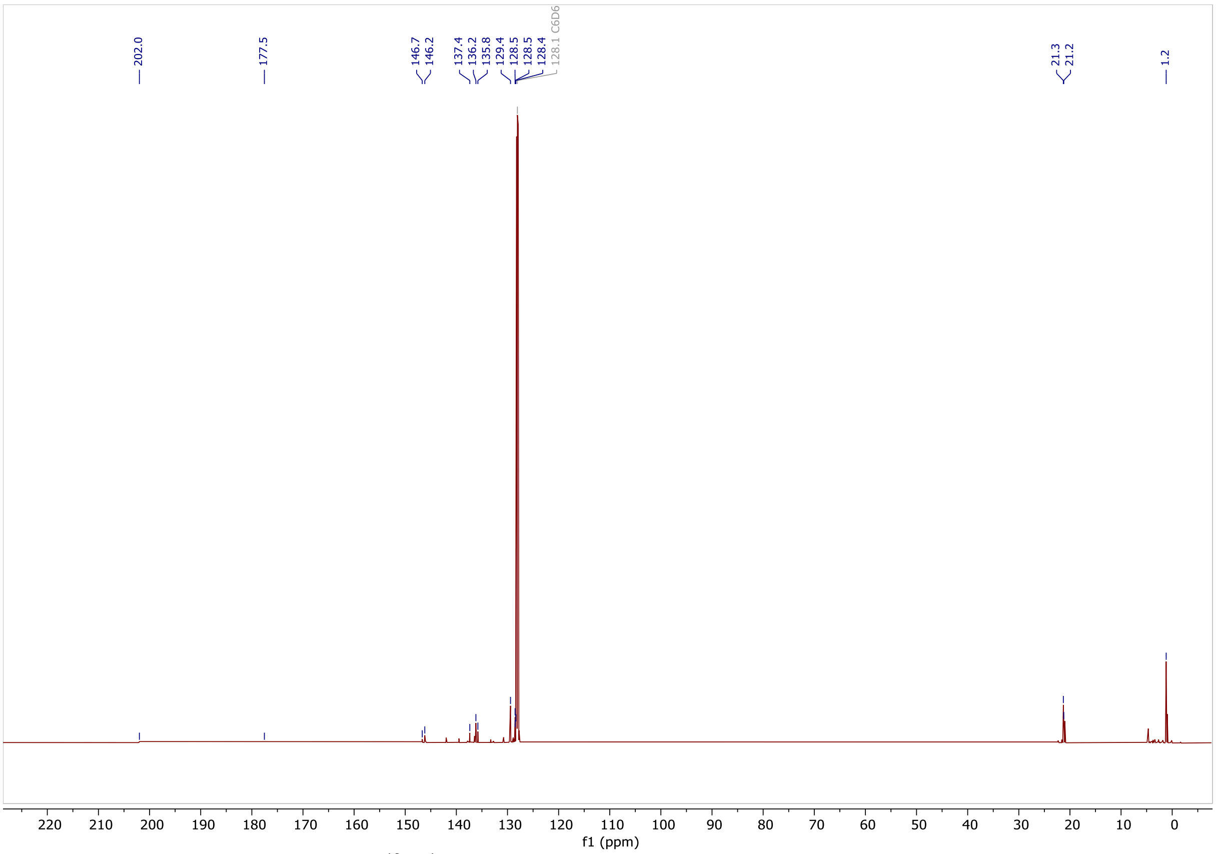

Figure S8. $^{13}C\{^{1}H\}$ NMR spectrum of product **3** in C_6D_6 at 298 K.

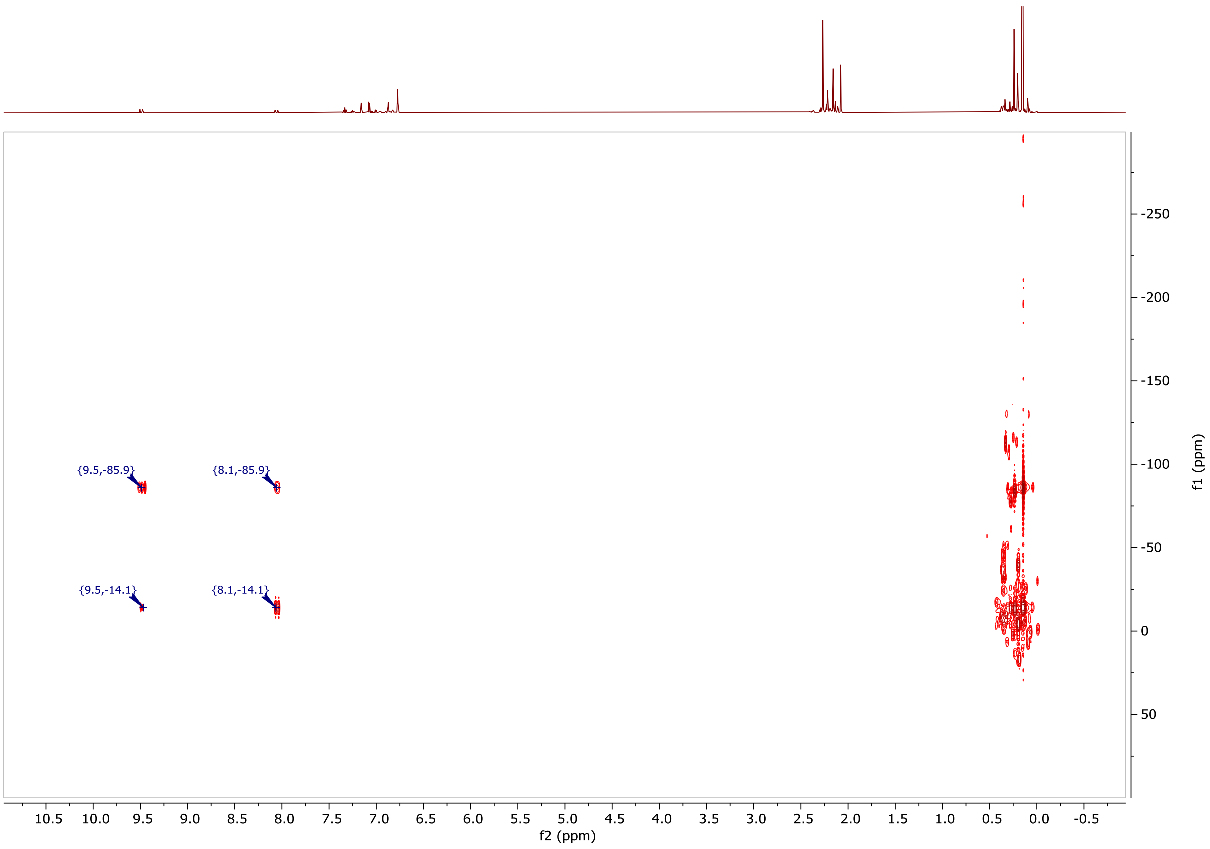

Figure S9. 2D $^{1}H/^{29}Si$ HMBC NMR spectrum of **3** in C_6D_6 at 298 K.

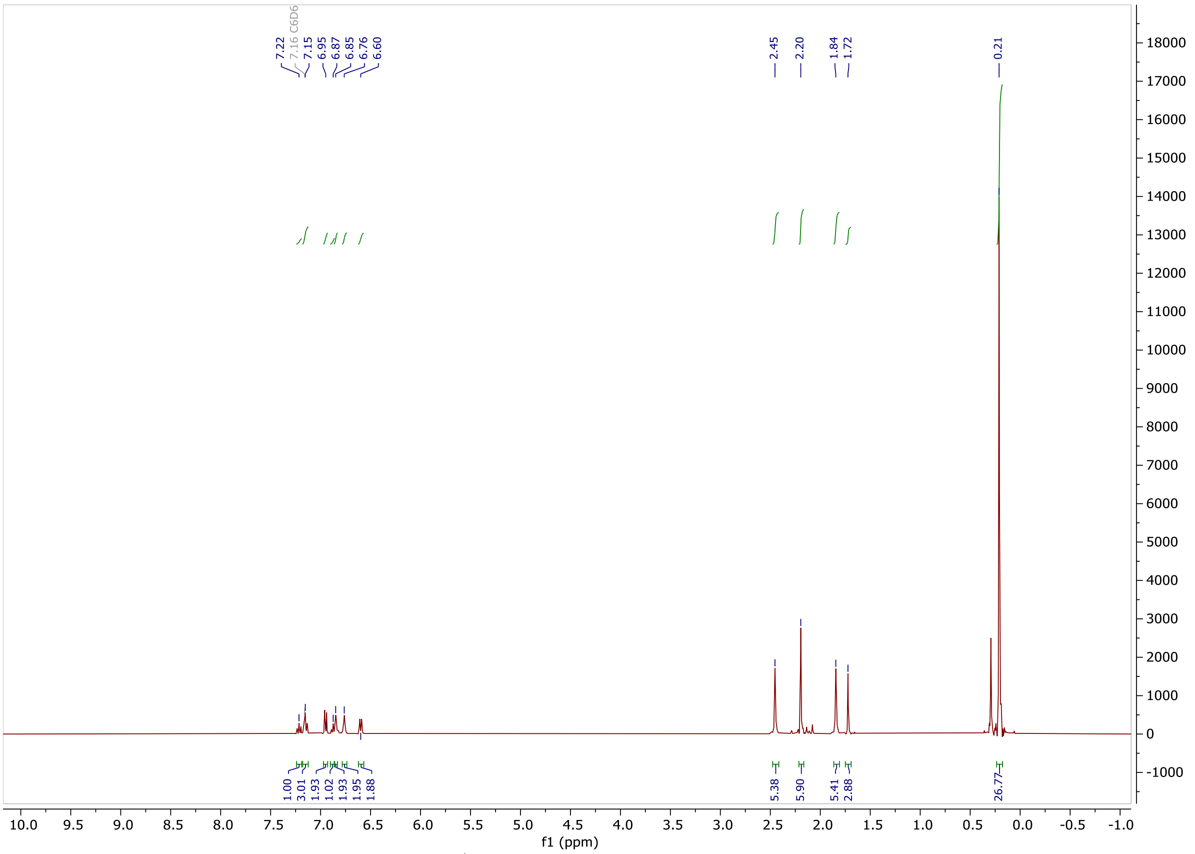

Figure S10. ^{1}H NMR spectrum of **4** in C_6D_6 at 298 K.

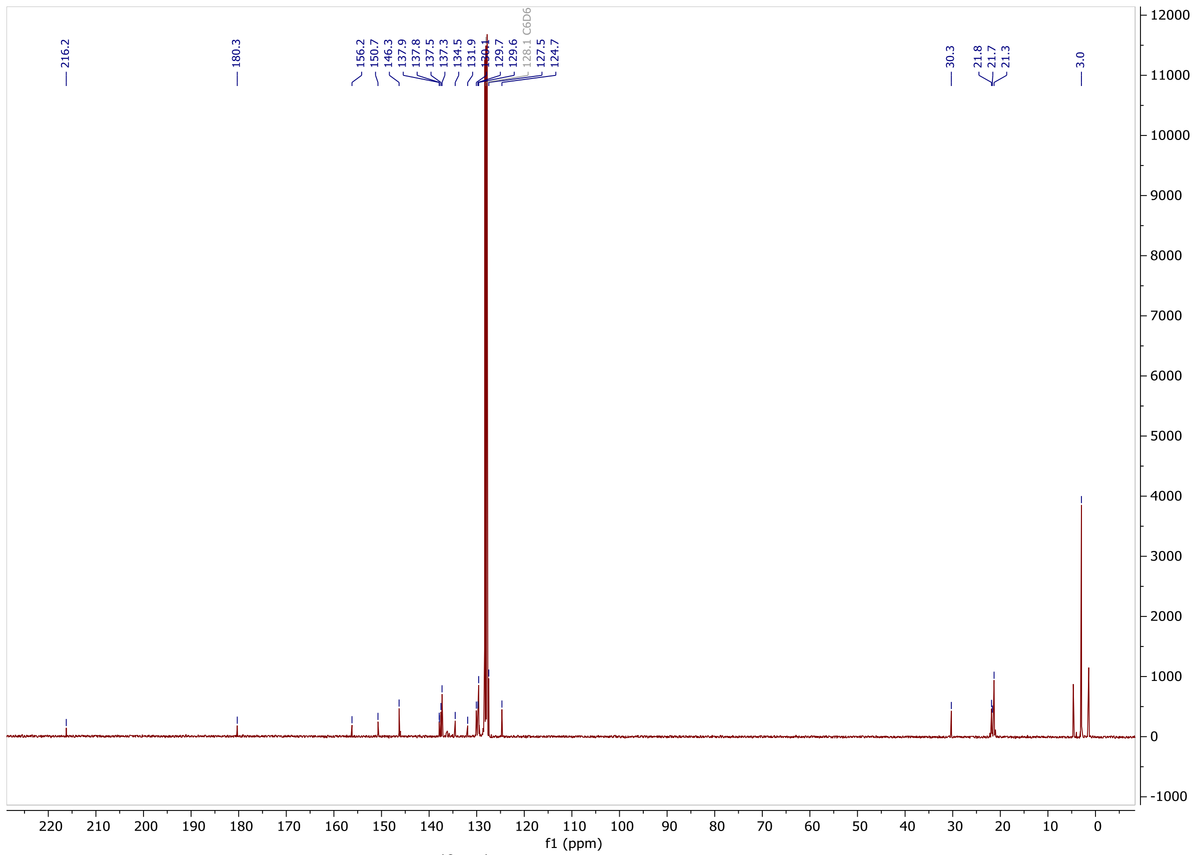

Figure S11. $^{13}C\{^{1}H\}$ NMR spectrum of **4** in C_6D_6 at 298 K.

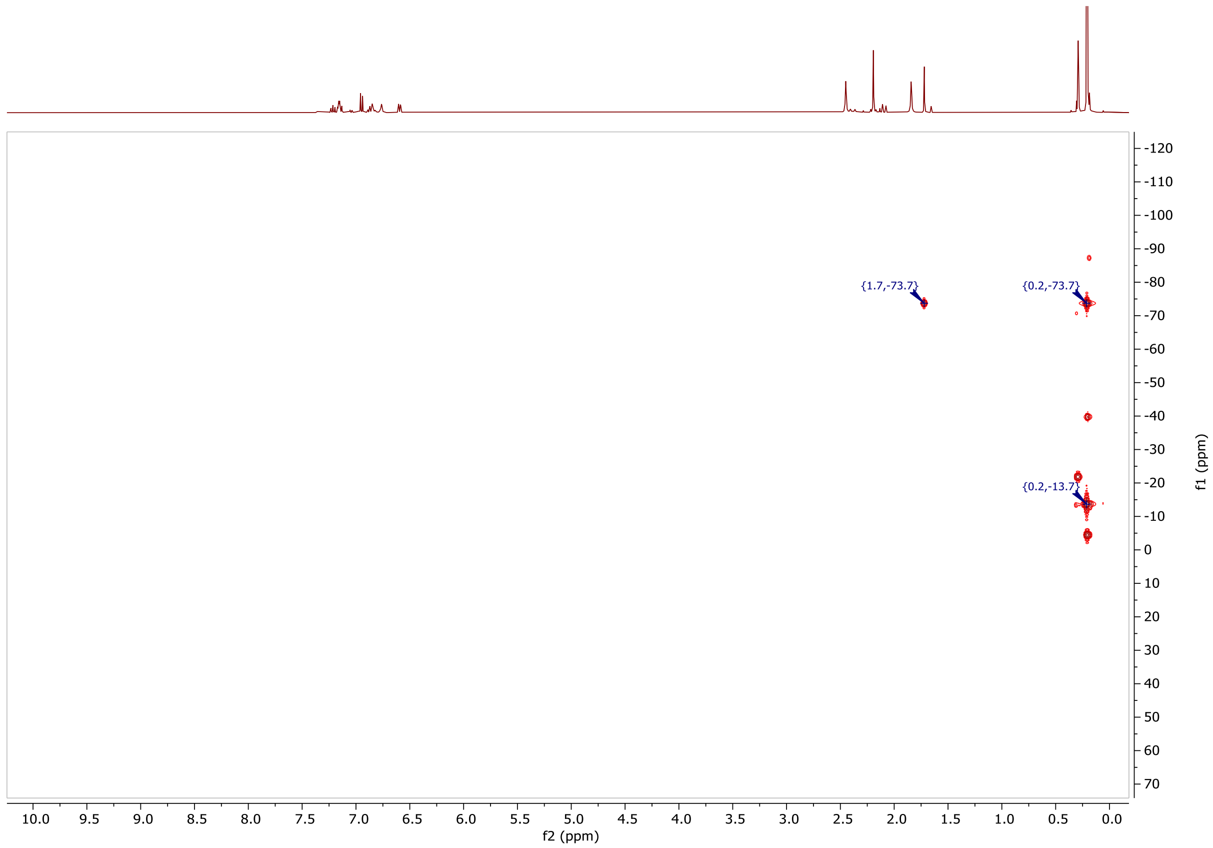

Figure S12. 2D $^{1}H/^{29}Si$ HMBC NMR spectrum of **4** in C_6D_6 at 298 K.

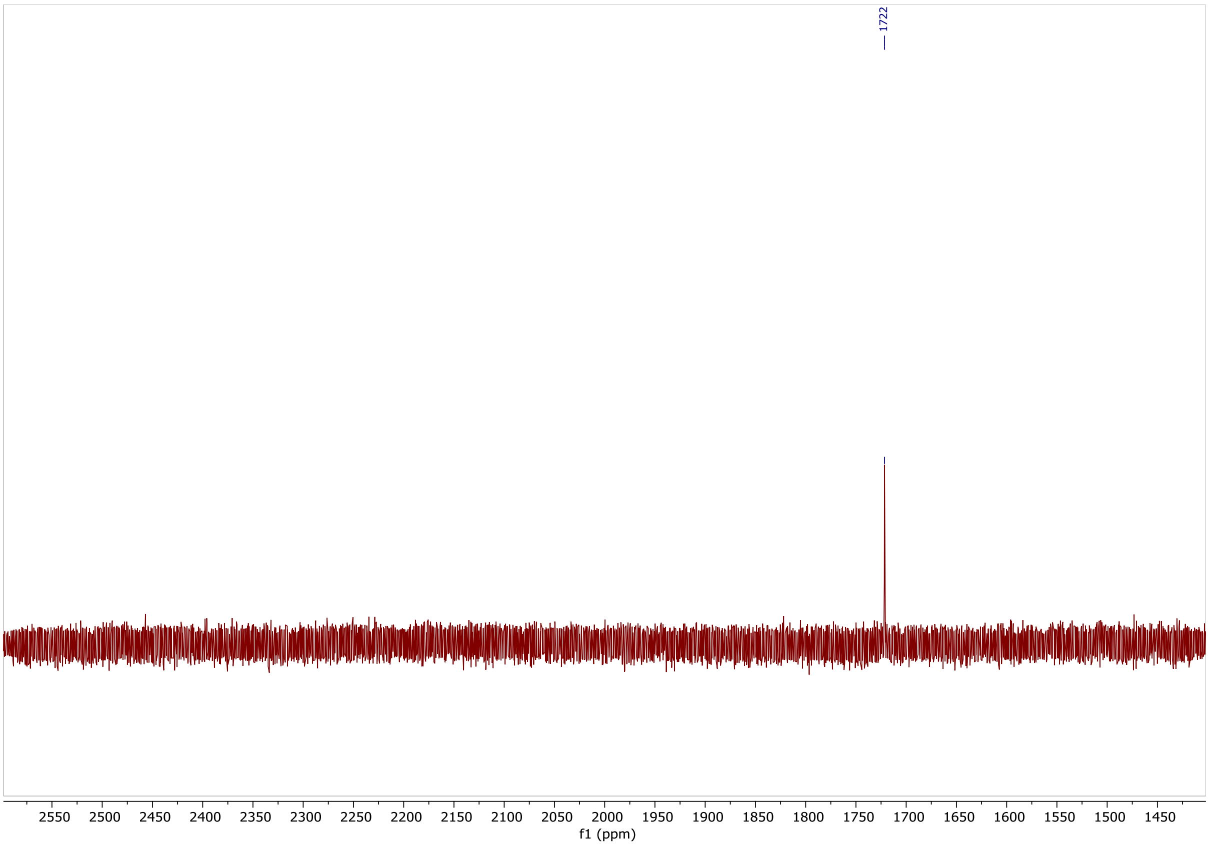

Figure S13. ^{119}Sn NMR spectrum of **4** in toluene-d_8 at 298 K.

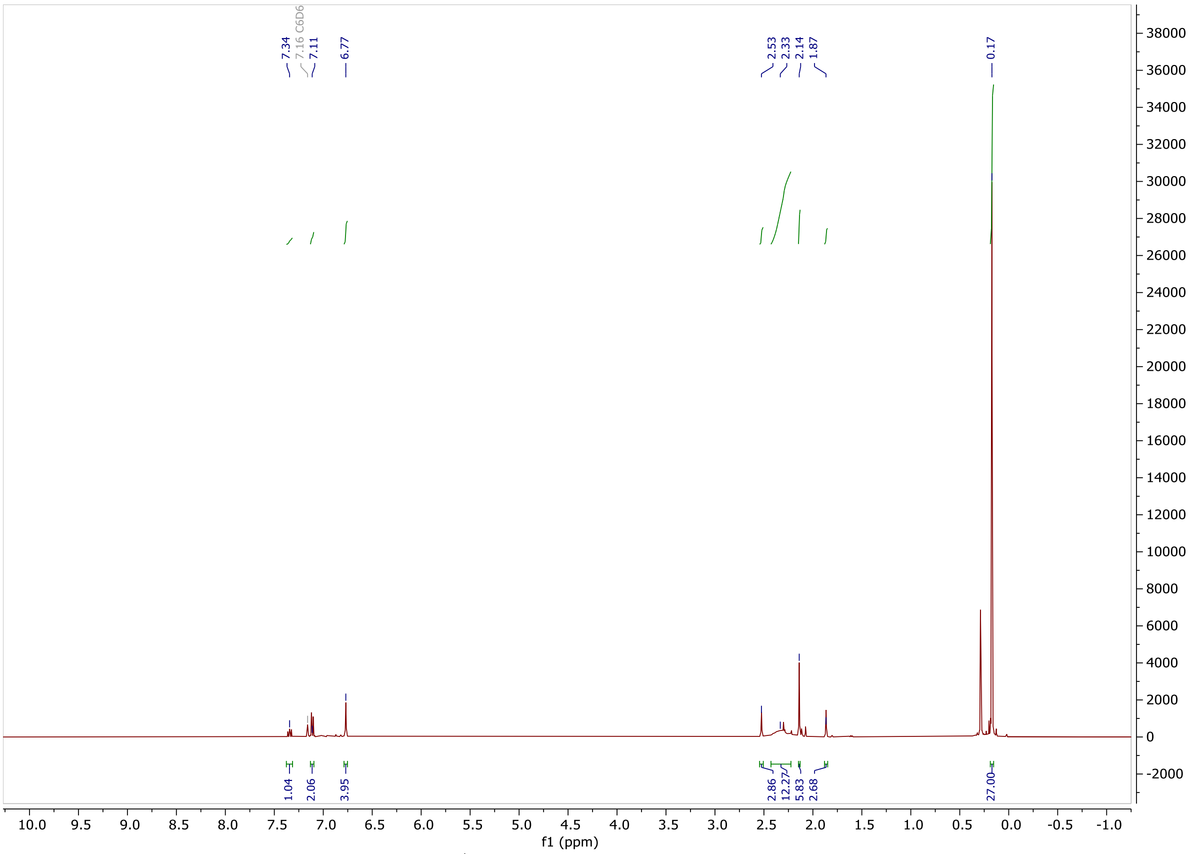

Figure S14. ^{1}H NMR spectrum of **5** in C_6D_6 at 298 K.

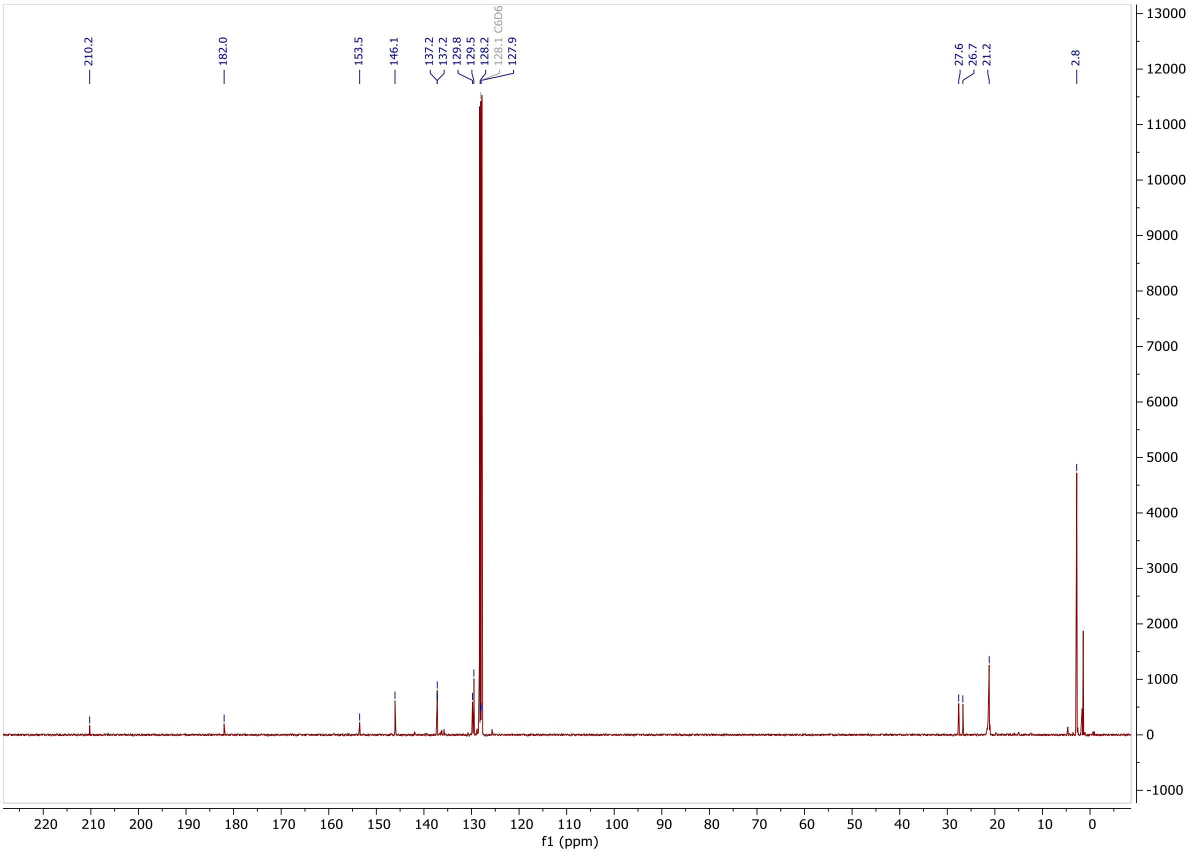

Figure S15. $^{13}C\{^{1}H\}$ NMR spectrum of **5** in C_6D_6 at 298 K.

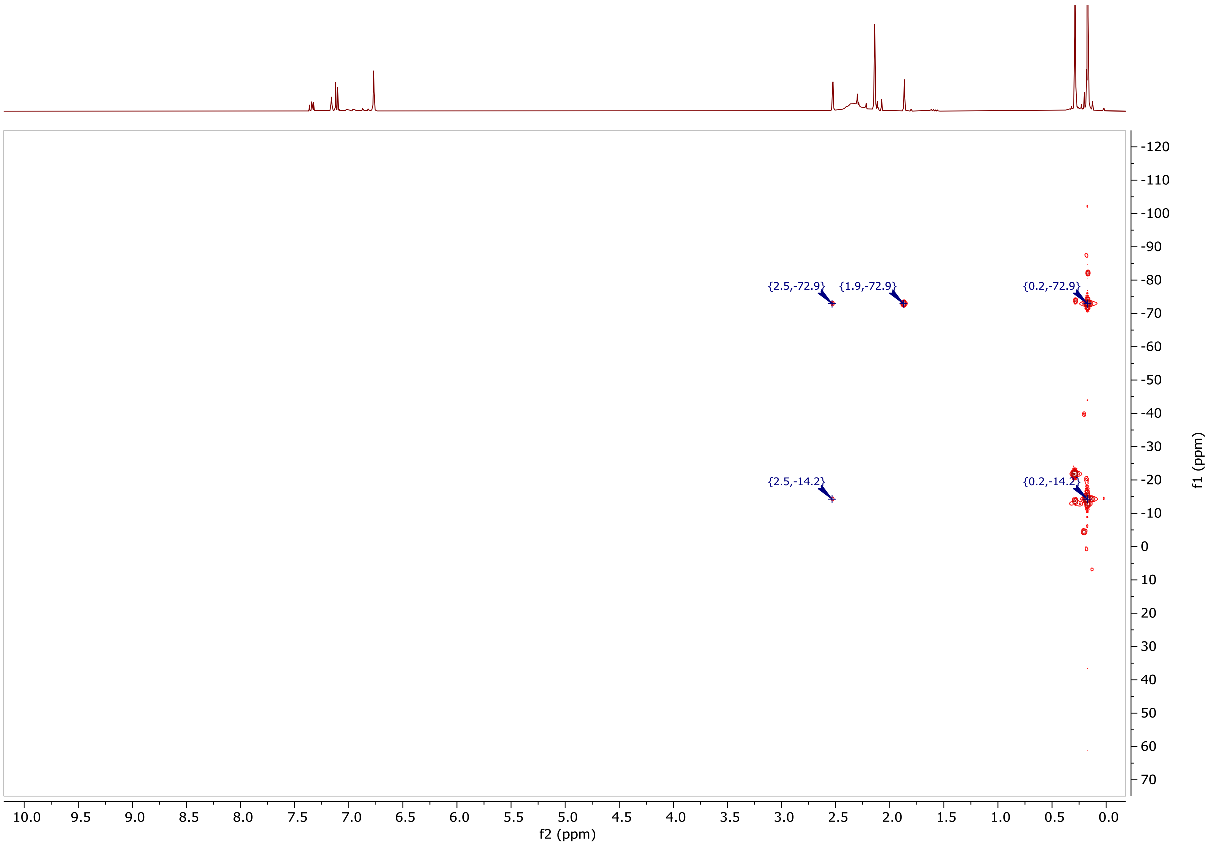

Figure S16. 2D $^{1}H/^{29}Si$ HMBC NMR spectrum of **5** in C_6D_6 at 298 K.

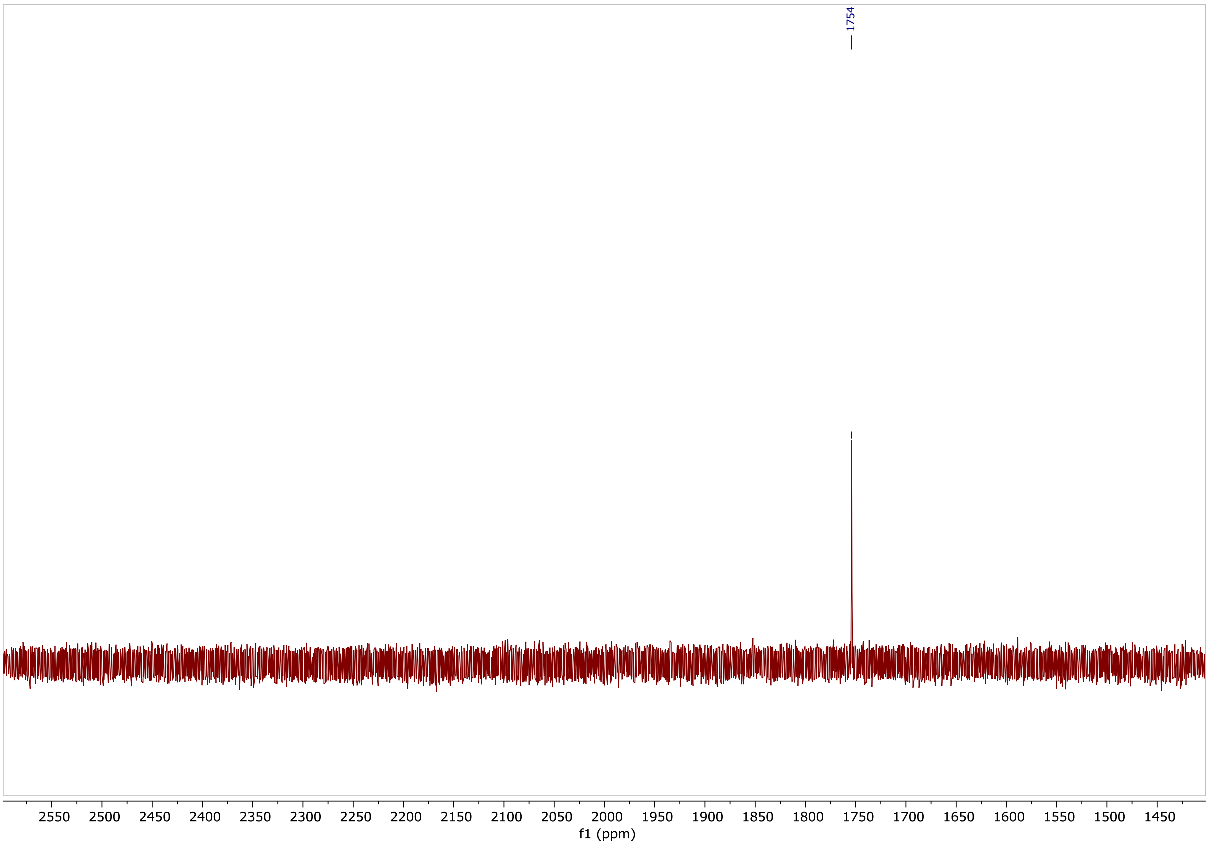

Figure S17. ^{119}Sn NMR spectrum of **5** in C_6D_6 at 298 K.

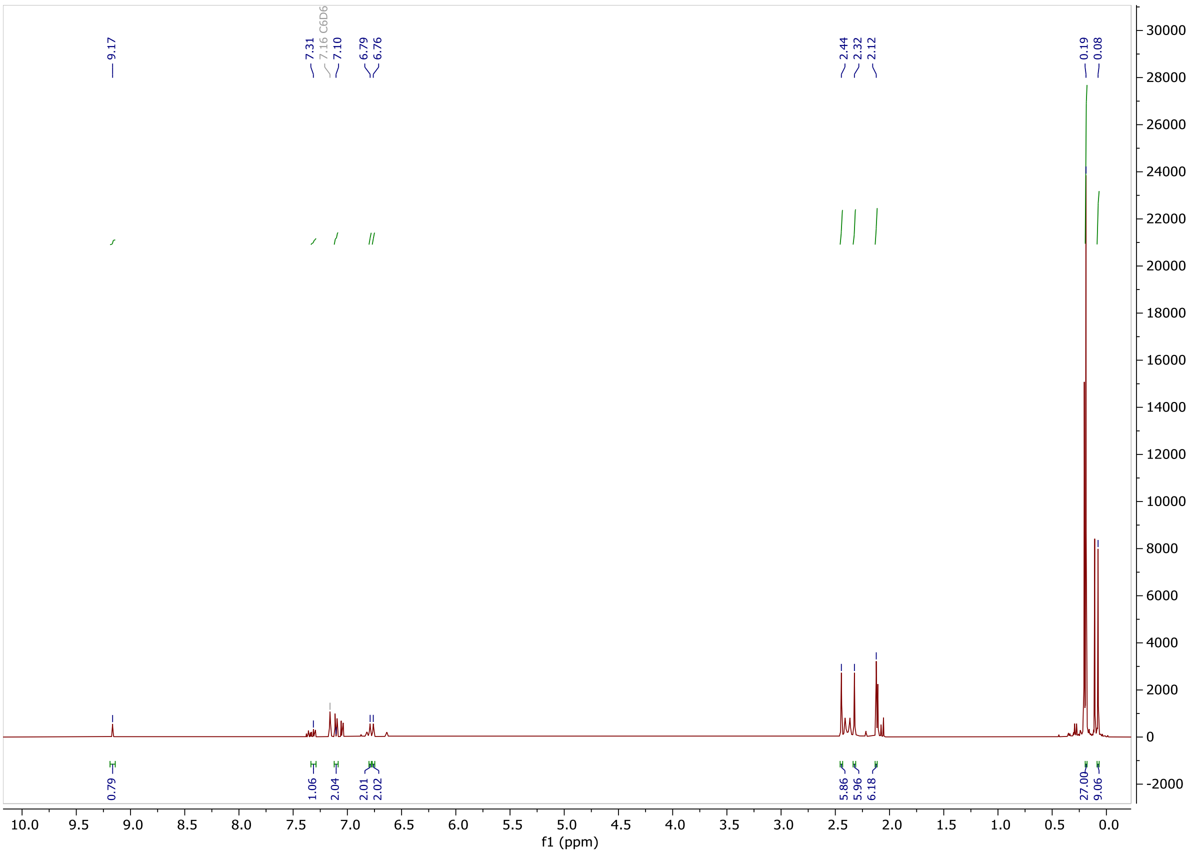

Figure S18. *In situ* ^{1}H NMR spectrum of **6** in C_6D_6 at 298 K.

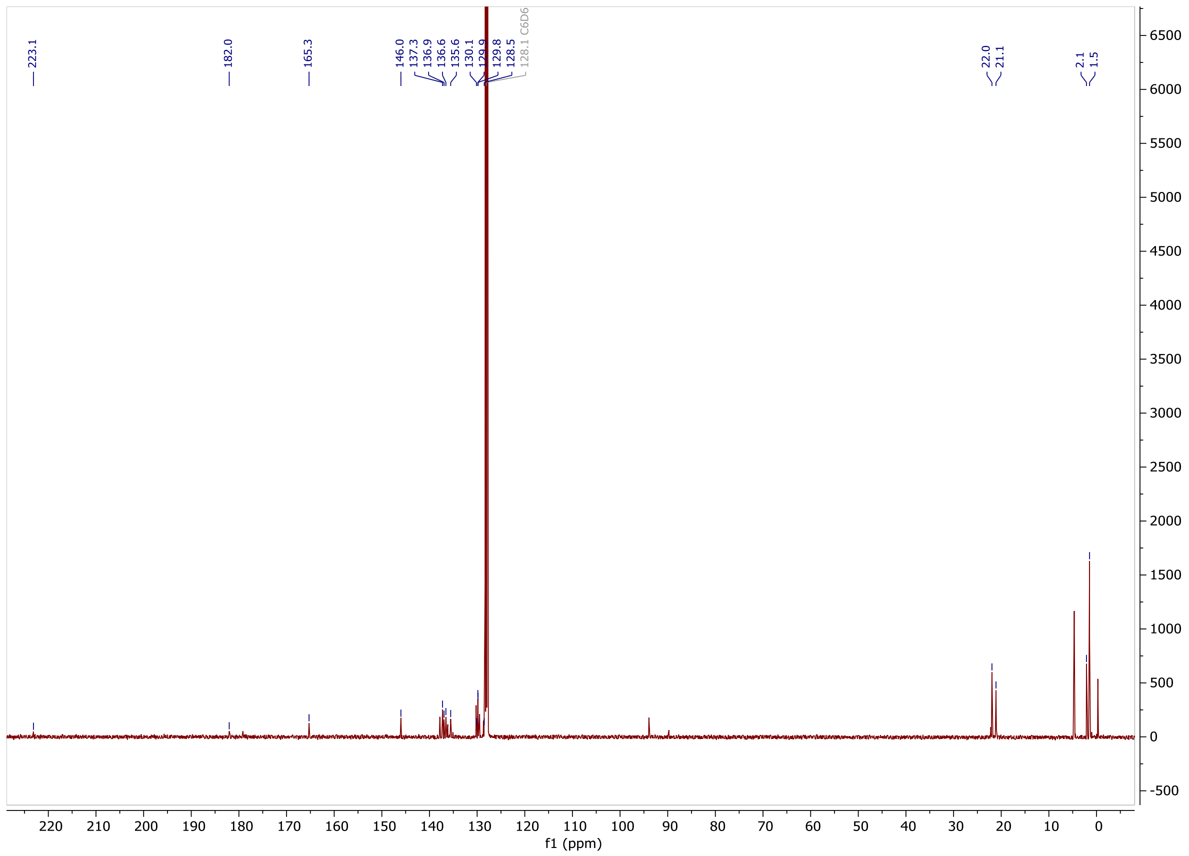

Figure S19. *In situ* $^{13}C\{^{1}H\}$ NMR spectrum of **6** in C_6D_6 at 298 K.

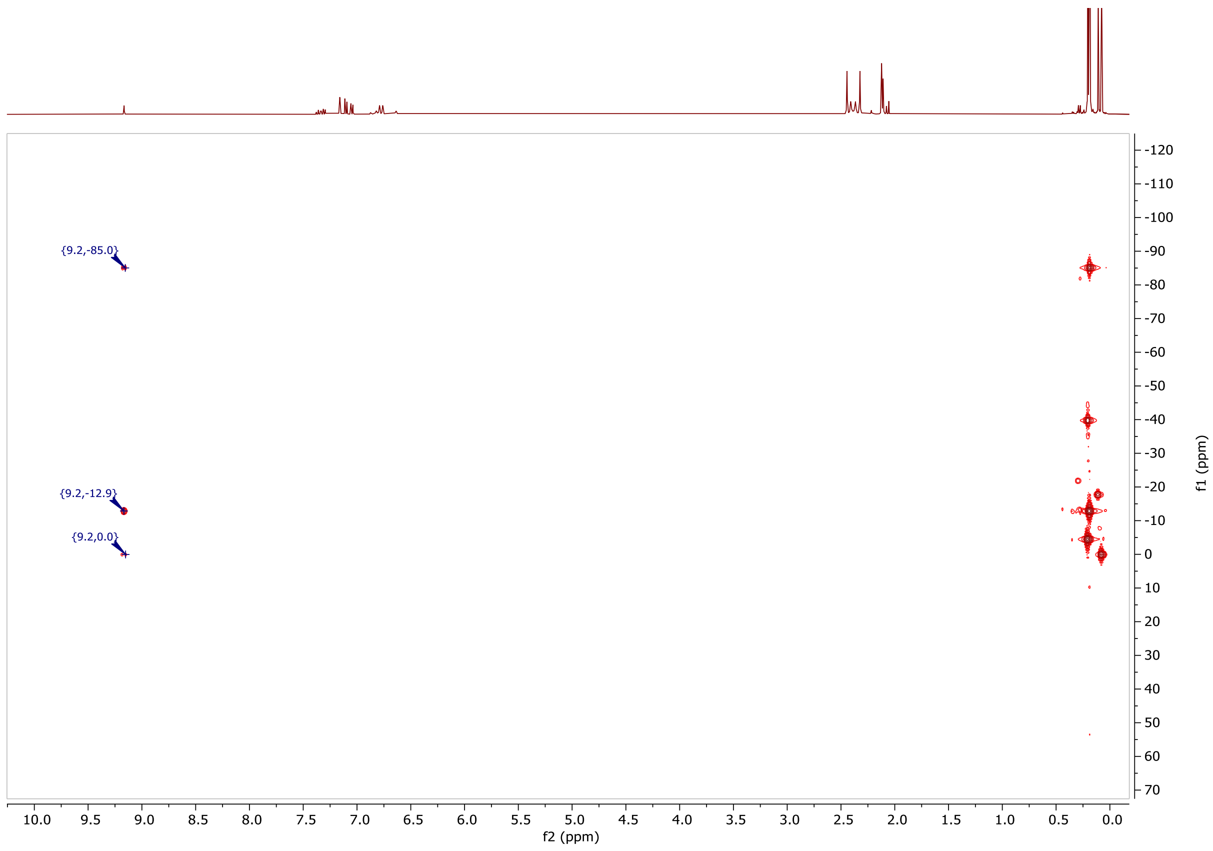

Figure S20. *In situ* 2D $^{1}H/^{29}Si$ HMBC NMR spectrum of **6** in C_6D_6 at 298 K.

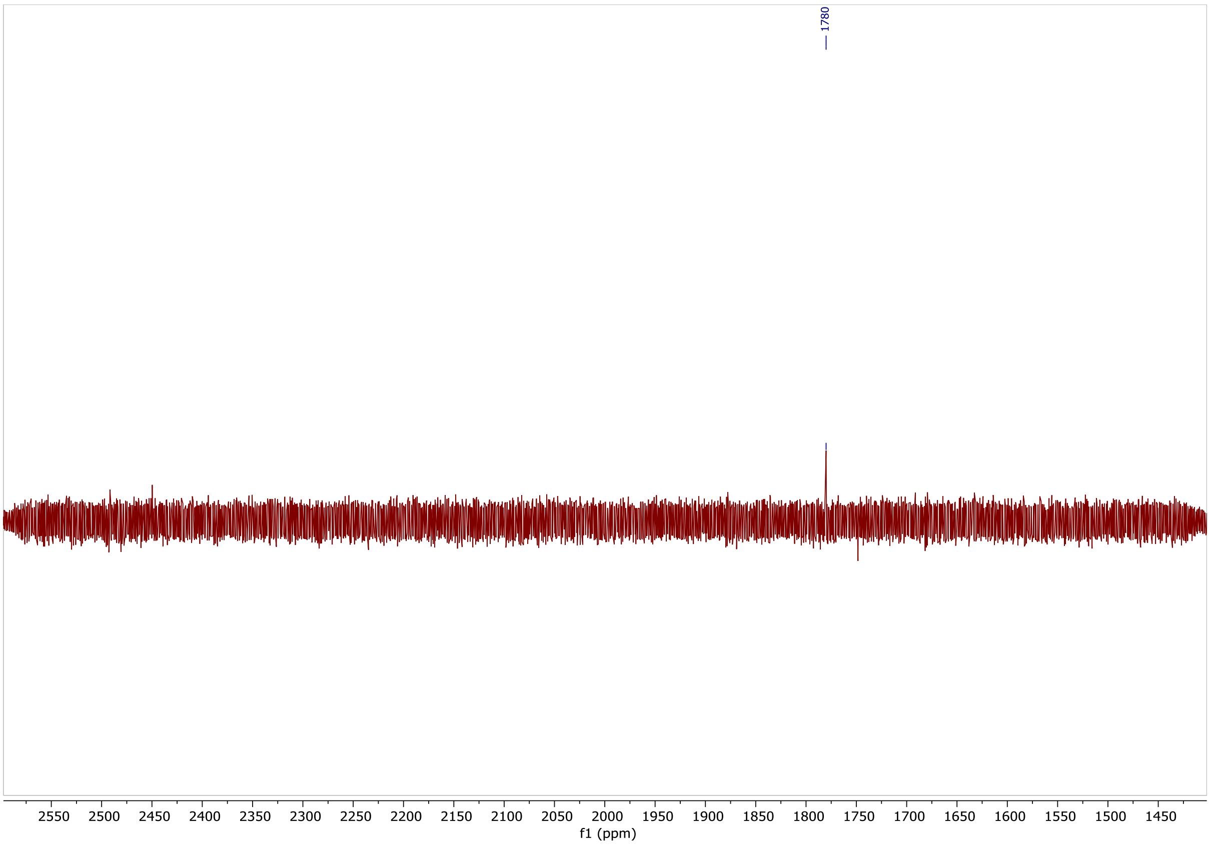

Figure S21. *In situ* ^{119}Sn NMR spectrum of **6** in C_6D_6 at 298 K.

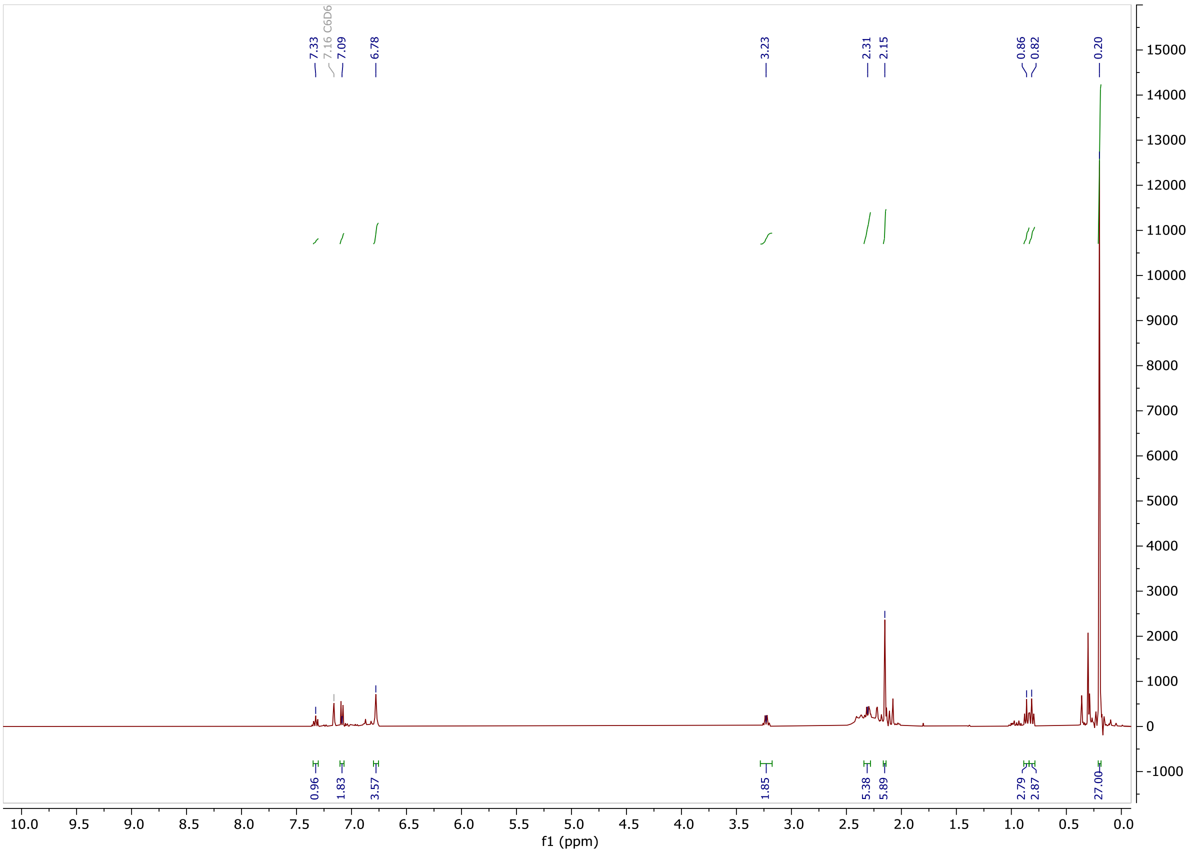

Figure S22. ^{1}H NMR spectrum of **7** in C_6D_6 at 298 K.

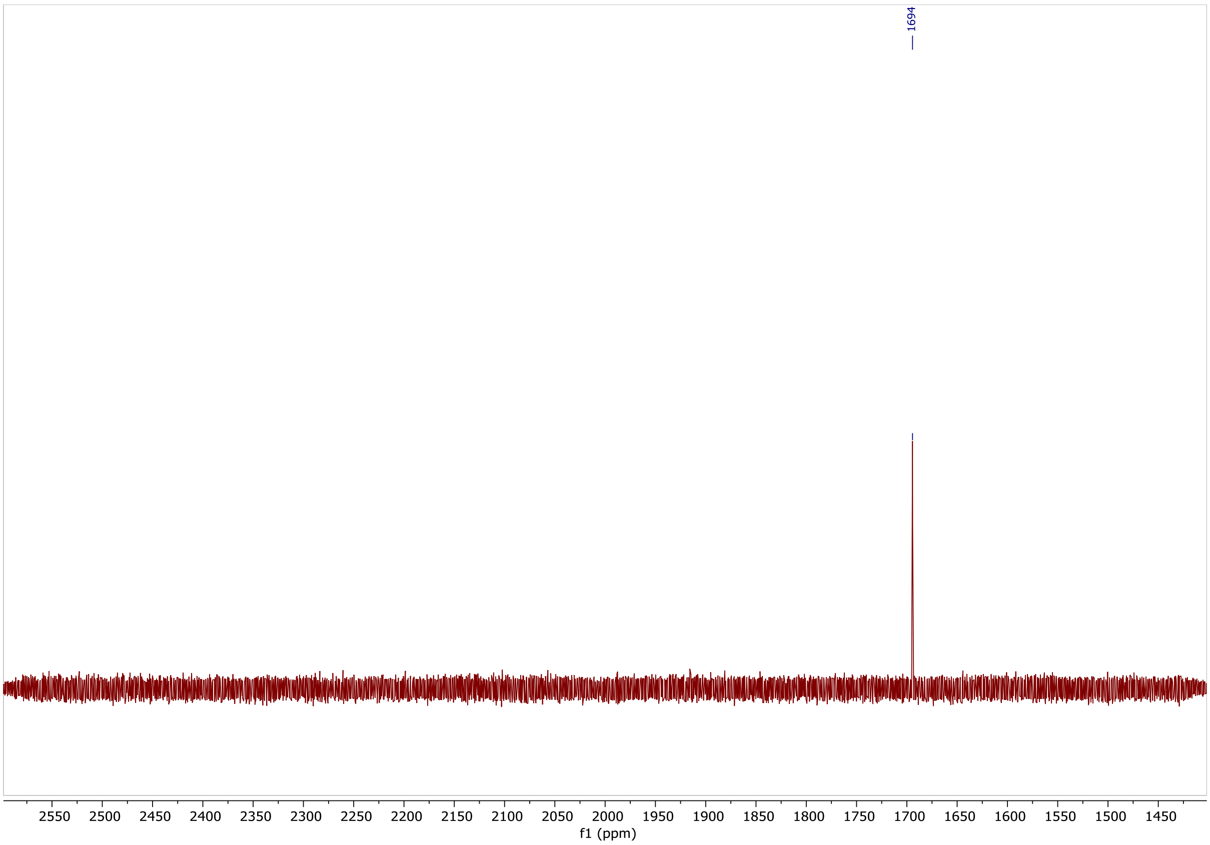

Figure S23. ^{119}Sn NMR spectrum of **7** in 3-hexyne at 298 K.

7. Quantum Chemical Calculations:

All quantum-chemical calculations were performed with ORCA 6.0.1.[S11–13] Geometries were optimised using the composite r^{2}SCAN-3c protocol:[S14] meta-GGA r^{2}SCAN,[S15] with the def2-TZVPPm basis,[S14] D4 dispersion correction,[S16] and the gCP geometrical counter-poise correction.[S17] Solvation was included throughout via the conductor-like polarizable continuum model CPCM (benzene, $\varepsilon \approx 2.27$).[S18,19] Tight geometry criteria (*TightOpt*) and an enlarged numerical grid (*DEFGRID2*) were applied. Harmonic frequency analyses confirmed every stationary point: minima have zero imaginary frequencies, whereas transition states exhibit one imaginary frequency along the reaction coordinate.

Single-point energies were refined - with the same CPCM (benzene) model - using the range-separated hybrid meta-GGA ωB97M functional[S20] augmented by the (re-)parametrised D4 dispersion correction[S21] and the def2-QZVPP basis set.[S22] Coulomb and exact-exchange integrals employed the RI and RIJCOSX approximations together def2/J auxiliary basis sets.[S23] Very tight SCF convergence thresholds and an ultrafine numerical grid (DEFGRID3) were used throughout.

All energies are reported as 298.15 K enthalpies in kJ mol^{-1}. Zero-point and thermal corrections obtained at the r2SCAN-3c level were added to the ωB97M-D4 electronic energies to yield the final values.

Xyz coordinates of optimised structures:

1:

```
    90
 Generated by Multiwfn
Sn      5.35618900      8.13570700      5.98806800
Si      3.04267700      7.81952800      7.25501700
Si      1.60646300      7.13742400      5.49738400
Si      2.43102600      6.65379000      9.21143200
Si      2.54899700     10.10946600      7.58807800
C       6.22784200      5.81786000      3.28302600
C       6.93827000      5.39127200      5.65536100
C       7.69966600      6.74565700      7.50681800
C       6.76733000      6.50459900      6.48794800
C       7.54485500      7.98437300      8.32588900
C       6.00483900      5.16747300      4.51205800
C       7.99738900      4.50623200      5.87855600
H       8.11706500      3.64100800      5.22938100
C       4.09480800      4.03864500      3.55479600
H       3.26957800      3.33662900      3.65965100
C       8.75829200      5.86136300      7.72154100
H       9.47556300      6.06117800      8.51508200
C       4.28051400      4.69713400      2.33908900
C       5.35439900      5.57757500      2.21995200
H       5.52601000      6.08432600      1.27209800
C       7.96301700      9.22958700      7.81543900
C       6.99919400      7.90671000      9.62441600
C       2.36995900      7.54390200      3.81671100
H       2.60598900      8.60975400      3.72394600
H       1.67445500      7.27874100      3.00965900
H       3.29431300      6.97403600      3.65652100
C       4.93735600      4.26102600      4.64321100
C       7.77790400     10.37917000      8.59287700
H       8.10104700     11.33933000      8.19435700
C       6.84588200      9.07180600     10.36974000
H       6.42626900      9.00739200     11.37241600
C       7.21985100     10.32078200      9.86504800
C       8.90131500      4.73719200      6.91188600
H       9.72712300      4.05020200      7.07586800
C       7.42380400      6.71174900      3.08631600
H       7.42217100      7.55224700      3.78975500
H       7.44200500      7.11736200      2.07168000
H       8.35488000      6.15923100      3.25767800
C       6.62143300      6.57217200     10.20551000
H       5.95968400      6.02459300      9.52667400
H       6.12091600      6.68853900     11.16975300
H       7.50735300      5.94436200     10.35262700
C      -0.09150300      7.96469100      5.59843100
H      -0.57555800      7.76347700      6.56035100
H      -0.73803200      7.56819200      4.80463700
H      -0.02977700      9.05029000      5.47185200
C       3.08581000      7.57898400     10.72506000
H       4.14518100      7.82993000     10.61756800
H       2.96286300      6.97468200     11.63262600
H       2.53564300      8.51622100     10.86640100
C       4.72396400      3.52555200      5.93783100
H       5.57053800      2.86663100      6.16212100
H       3.81719100      2.91593100      5.90041600
H       4.64154800      4.22562700      6.77651800
C       0.89238300     10.37869200      8.46139600
H       0.06036900      9.93020000      7.90920000
H       0.69681200     11.45437500      8.55818400
H       0.90180100      9.94777800      9.46838800
C       8.72564200      9.33911600      6.51939300
H       9.80255600      9.26749500      6.72063100
H       8.54194100     10.30256200      6.03550100
H       8.47658500      8.53908600      5.81926700
C       3.04269200      4.86671500      9.22703000
H       2.60592700      4.30374600      8.39460100
H       2.73944400      4.37765400     10.16153800
H       4.13121100      4.79425000      9.14433600
C       0.54497600      6.58415200      9.36599500
H       0.09940000      7.58399900      9.35036900
H       0.27169100      6.10636400     10.31600500
H       0.09421000      6.00109700      8.55610100
C       2.51499200     11.04142600      5.94166100
H       3.46519200     10.93230900      5.40666800
H       2.35024900     12.11112100      6.12371700
H       1.71670400     10.68540200      5.28205500
C       3.89136600     10.89980900      8.65946000
H       3.99317200     10.38138700      9.61917800
H       3.65383800     11.95239400      8.86061100
H       4.86961500     10.86144500      8.16466700
C       1.32091100      5.27160900      5.58114600
H       0.77617300      4.93414600      4.68979900
H       0.73350400      4.99461400      6.46297400
C       3.32168500      4.48801400      1.19962800
H       2.93197100      3.46563500      1.18952200
H       3.79655000      4.69062700      0.23534400
H       2.46328700      5.16533700      1.29374800
C       7.00141000     11.56860900     10.67434800
H       7.64849700     12.38202500     10.33438500
H       7.19231600     11.39126600     11.73735400
H       5.96229100     11.90819500     10.57987100
H       2.27070800      4.73215000      5.62039300
```

Reaction of **1** with phenylacetylene to form **2**:

T1:

```
    104
 Generated by Multiwfn
Sn      5.32701500      7.91689300      5.63165600
Si      3.65424400      7.92424600      7.80391000
Si      1.54214500      6.82995500      7.73900100
Si      4.34604900      7.76714400     10.06738500
Si      3.11988900     10.21949400      7.52142000
C       5.43144000      3.85237400      3.80910800
```

```
C       6.95125900       4.97588200       5.45389500
C       8.18266900       6.97280400       6.00474000
C       6.94070700       6.34475300       5.78087800
C       8.22638500       8.42944600       6.34226800
C       5.71438300       4.20389400       5.14104200
C       8.16225200       4.27370000       5.39333000
H       8.14167700       3.21308500       5.15067100
C       3.74153600       3.00649300       5.85695800
H       3.09415300       2.65971300       6.66041300
C       9.38344000       6.26478500       5.93525000
H      10.32181800       6.78402900       6.12067000
C       3.43921600       2.65603200       4.54029600
C       4.30212300       3.08135000       3.53052000
H       4.08043400       2.82378100       2.49638800
C       8.30684900       9.40044300       5.32258200
C       8.27642300       8.82482200       7.69297600
C       0.79086000       6.53816200       6.02471800
H       1.18060400       5.63096800       5.55385400
H       0.96480000       7.37316500       5.33913500
H      -0.29292900       6.41166100       6.14314700
C       4.87199600       3.76434500       6.17543400
C       8.35847100      10.74920800       5.67398100
H       8.40322600      11.49805100       4.88540600
C       8.34290500      10.18307900       8.00312300
H       8.38524000      10.48653200       9.04766900
C       8.36497200      11.15904200       7.00698100
C       9.37454000       4.90773800       5.63476000
H      10.30503500       4.34875400       5.58427200
C       6.31019900       4.32624500       2.68303300
H       6.55463200       5.38813700       2.79869900
H       5.81570100       4.18286700       1.71850800
H       7.26350500       3.78550100       2.66204000
C       8.31964000       7.78578100       8.77910900
H       7.47928200       7.08930200       8.68965100
H       8.29317900       8.24450200       9.76996500
H       9.23223200       7.18370600       8.70201500
C       0.23706000       7.85178700       8.65858800
H       0.53737700       8.12668800       9.67283300
H      -0.68322900       7.25626900       8.72524700
H      -0.00099900       8.77038000       8.11123900
C       5.51064800       9.20416500      10.47301000
H       6.25793500       9.36536300       9.69218900
H       6.03284700       9.02239700      11.42095600
H       4.93689200      10.13196800      10.57801800
C       5.18732400       4.09083300       7.60730900
H       6.23721100       3.88195300       7.83874800
H       4.55592600       3.51780300       8.29128100
H       5.02766900       5.15816300       7.80635600
C       2.17377000      11.01294100       8.95831300
H       1.24305000      10.48888100       9.19301200
H       1.92390900      12.04912400       8.69536400
H       2.78441600      11.03987900       9.86723700
C       8.38594700       8.99157400       3.87745400
H       9.35107100       8.51547400       3.66433900
H       8.27610000       9.85835800       3.22045500
H       7.60887800       8.26199300       3.62242800
C       5.17382000       6.13096800      10.53552600
H       4.48922600       5.28906100      10.38393900
H       5.44266500       6.15880100      11.59948100
H       6.08281000       5.92635600       9.96286000
C       2.88230000       7.94731200      11.25873200
H       2.33653000       8.88150500      11.09251500
H       3.26873400       7.96121600      12.28669100
H       2.17231400       7.11759600      11.18055100
C       2.08021400      10.41171400       5.94919000
H       2.61303100      10.00106200       5.08268400
H       1.87560500      11.47103800       5.74824400
H       1.12073200       9.88877500       6.03243900
C       4.68491400      11.26386300       7.29631800
H       5.34215400      11.19868000       8.17045200
H       4.40807500      12.31719900       7.15705400
H       5.26924100      10.95278400       6.42207000
C       1.64802100       5.14405300       8.59062400
H       2.33488600       4.48220200       8.05429300
H       0.65868500       4.66882900       8.60408300
C       2.19823800       1.86974800       4.21554100
H       1.89966200       1.23363000       5.05392000
H       2.34808200       1.23851700       3.33468000
H       1.35882100       2.54214200       3.99740300
C       8.36825700      12.61951700       7.36165400
H       8.90093800      13.21191000       6.61182900
H       8.83009600      12.79222400       8.33812200
H       7.33945100      12.99872400       7.41148900
C       3.50747500       7.08588900       3.27658600
C       3.38106000       6.14871900       4.03672300
H       1.99899200       5.22679200       9.62468700
H       3.26322600       5.30509300       4.67949800
C       3.61832000       8.16894600       2.36422800
C       2.54255800       9.05288500       2.18552200
C       4.80639000       8.36806000       1.64239900
C       2.65923700      10.11628400       1.30007900
H       1.62676500       8.89716400       2.74720500
C       4.91172200       9.43399500       0.76065200
H       5.63665500       7.68423200       1.78922800
C       3.84100300      10.31028600       0.58795400
H       1.82453000      10.79816400       1.16718300
H       5.83342000       9.58404300       0.20618900
H       3.92826000      11.14474800      -0.10167600
```

I1:

```
 104
 Generated by Multiwfn
Sn      5.34215500       7.94106700       5.63297300
Si      3.63163600       7.91271300       7.78730800
Si      1.53021200       6.79929500       7.73660300
Si      4.33608400       7.76497800      10.04741900
Si      3.08968300      10.20843700       7.51824700
C       5.39954900       3.91740000       3.80262300
C       6.95947600       4.98782300       5.44458700
C       8.20850200       6.97294800       5.99786100
C       6.95914400       6.35794200       5.76790400
C       8.27515300       8.42703400       6.34329300
C       5.71433200       4.22745400       5.13688800
C       8.16291200       4.27310300       5.38291000
H       8.13111000       3.21293600       5.13931000
C       3.74000400       3.03942400       5.86151500
H       3.10362600       2.68015200       6.66822300
C       9.40135700       6.25094900       5.92964800
H      10.34419300       6.76055300       6.11920000
C       3.40288500       2.73449500       4.54176200
C       4.25061900       3.17479400       3.52605700
H       4.00297200       2.95077900       2.49000000
C       8.37558800       9.40112600       5.33008200
C       8.30764800       8.81563900       7.69548300
C       0.78234900       6.48247400       6.02382000
H       1.18843600       5.58099200       5.55507700
H       0.93546100       7.31902000       5.33498600
H      -0.29820400       6.33317600       6.14584800
C       4.88766300       3.77184300       6.17644800
C       8.42939600      10.74810700       5.68713500
H       8.48776300      11.49999200       4.90226200
C       8.37422700      10.17273600       8.01286300
H       8.40027600      10.47119100       9.05945100
C       8.41485200      11.15294400       7.02180200
C       9.38080100       4.89507200       5.62547000
H      10.30628900       4.32779100       5.57474400
C       6.26414100       4.41127600       2.67492500
H       6.50652600       5.47163900       2.80856800
H       5.76020800       4.28366500       1.71308900
H       7.21901000       3.87431300       2.63624300
C       8.32194600       7.77245600       8.77898100
H       7.47187600       7.08943000       8.67744000
H       8.28952900       8.22884100       9.77082300
H       9.22580400       7.15616100       8.71179700
C       0.21247500       7.81380100       8.64614400
H       0.50911400       8.09703500       9.65924300
H      -0.70280000       7.21093000       8.71504700
H      -0.03176000       8.72760100       8.09338600
C       5.50025600       9.20433100      10.44551100
H       6.24551900       9.36470300       9.66240100
H       6.02527400       9.02447400      11.39226900
H       4.92583400      10.13170000      10.55033200
C       5.23053200       4.06883900       7.60825700
H       6.27827900       3.83283300       7.82268600
H       4.59518900       3.50122200       8.29318700
H       5.09737600       5.13673700       7.82310400
C       2.12771800      10.98899000       8.95211700
H       1.20454400      10.45234300       9.18798900
H       1.86336300      12.02084900       8.68637800
H       2.73800900      11.02734500       9.86096100
C       8.45285800       8.99668500       3.88386600
H       9.39728300       8.47942500       3.67495000
H       8.38417000       9.86989200       3.22982900
H       7.64616400       8.30198100       3.62151400
C       5.16546200       6.13292700      10.52679400
H       4.48526700       5.28793700      10.37309100
H       5.42449100       6.16669200      11.59307600
H       6.08068100       5.92924500       9.96388100
C       2.87191100       7.94863500      11.23798400
H       2.32140900       8.87829600      11.06301300
H       3.25733500       7.97318400      12.26609700
H       2.16626800       7.11475600      11.16607500
C       2.05952200      10.40282900       5.93963300
H       2.60559700      10.00907600       5.07357700
H       1.83880600      11.46034700       5.74672500
H       1.10814400       9.86322600       6.01070500
C       4.64753600      11.26890600       7.31514800
H       5.29404900      11.20639500       8.19739300
H       4.35916600      12.31968800       7.17978900
H       5.24544100      10.96937300       6.44656000
C       1.65250400       5.12006300       8.59972500
H       2.36391800       4.46986200       8.08117700
H       0.67319400       4.62444800       8.59536900
C       2.14197800       1.97785800       4.22292400
H       1.85978500       1.31172900       5.04355700
H       2.25633200       1.38103500       3.31325000
H       1.30572200       2.66913700       4.05828700
C       8.41505300      12.61225200       7.38260700
H       8.95550600      13.20721000       6.64032500
H       8.86803000      12.78080200       8.36402300
H       7.38652200      12.99312600       7.42459700
C       3.59656500       7.16086400       3.40568100
C       3.50033900       6.27375600       4.23125100
H       1.98109400       5.21499000       9.64003800
H       3.34806100       5.44233600       4.88317900
C       3.66783500       8.18142500       2.42101200
C       2.56203900       9.01807600       2.20026100
C       4.83573400       8.35799800       1.66127800
C       2.62998400      10.01401400       1.23523800
H       1.66226900       8.87770200       2.79106100
C       4.89222400       9.35762000       0.70131900
H       5.68845900       7.71058000       1.84002500
C       3.79209300      10.18723700       0.48646900
```

H	1.77274300	10.65948500	1.06858500
H	5.79812400	9.49204100	0.11771100
H	3.84133700	10.96955300	-0.26525800

T2:

104
Generated by Multiwfn

Sn	5.18760000	8.18280000	6.15830000
Si	2.65340000	7.30000000	6.85700000
Si	1.34193700	9.01411100	5.87084300
Si	1.32074500	5.32772600	6.81218100
Si	2.91651300	7.83487900	9.15769600
C	6.53810800	4.98095600	3.50921000
C	7.52202400	5.86568000	5.64354300
C	8.13752400	7.75257100	7.01787400
C	7.14460100	7.05181600	6.29998100
C	7.77355200	8.97278600	7.80140400
C	6.54137700	5.01252800	4.91417800
C	8.84944700	5.42223400	5.69065200
H	9.11434300	4.49804900	5.18119000
C	4.77028100	3.37285200	4.94382000
H	4.10295500	2.72307600	5.50811100
C	9.45851300	7.30062700	7.05360200
H	10.19987900	7.86470600	7.61574300
C	4.72132100	3.35884600	3.55077900
C	5.61783200	4.16689600	2.85062900
H	5.60492400	4.16065400	1.76152200
C	7.63690100	10.22507100	7.17878500
C	7.55537900	8.85424800	9.18866100
C	2.37508800	10.52900500	5.41660900
H	2.88268300	10.94841800	6.29198300
H	1.72132600	11.30249300	4.99294000
H	3.13790000	10.27935400	4.67109400
C	5.66424100	4.19107400	5.63797500
C	7.20345500	11.31939400	7.93324800
H	7.08127700	12.28282200	7.44088100
C	7.12735900	9.96839300	9.90716400
H	6.93842800	9.86679700	10.97470400
C	6.92530100	11.20565300	9.29226600
C	9.81804700	6.13764000	6.38337100
H	10.84553000	5.78447200	6.40919600
C	7.51889000	5.79916700	2.71195000
H	7.66673400	6.78914400	3.15250600
H	7.17175200	5.92715300	1.68296100
H	8.50153600	5.31216000	2.68202700
C	7.81719200	7.55567900	9.90498300
H	7.46945100	6.69359100	9.32836300
H	7.33149000	7.54589300	10.88417800
H	8.89401500	7.41441300	10.06008500
C	-0.01839200	9.59081100	7.05448900
H	-0.66923700	8.76855800	7.36803000
H	-0.64086400	10.34350000	6.55372200
H	0.40230900	10.05033000	7.95529400
C	1.92311000	4.01540100	8.03188600
H	2.91778900	3.64511100	7.76647000
H	1.23145400	3.16330400	8.02814300
H	1.97121400	4.40495200	9.05442800
C	5.67867600	4.17328000	7.14311200
H	6.69513300	4.26245100	7.53776700
H	5.23361200	3.24983800	7.52432900
H	5.10414900	5.01491700	7.55208400
C	1.33570500	7.55760400	10.16283000
H	0.49503200	8.13582100	9.76531300
H	1.50576100	7.87782200	11.19895500
H	1.04553000	6.50191500	10.18270900
C	8.00681900	10.42890900	5.73228800
H	9.03824300	10.79737900	5.65872800
H	7.35802600	11.17373400	5.26062900
H	7.96085800	9.49921600	5.15989000
C	1.16760900	4.50526800	5.11457600
H	0.81710700	5.20592400	4.34866800
H	0.43254800	3.69302300	5.18439900
H	2.11334700	4.06743500	4.77880400
C	-0.44058000	5.80447100	7.32226800
H	-0.47109400	6.33063500	8.28089100
H	-1.04404300	4.89236600	7.41796400
H	-0.91647500	6.44256900	6.57013500
C	3.40392000	9.65057200	9.35234500
H	4.27156300	9.91162800	8.73589000
H	3.67136700	9.84846000	10.39848300
H	2.57783300	10.31705500	9.08139900
C	4.27965800	6.76708700	9.90853400
H	4.01591200	5.70378700	9.88645500
H	4.46670300	7.05334700	10.95126500
H	5.21532900	6.89255500	9.35454000
C	0.51639400	8.39055000	4.28791200
H	1.26798500	8.08484300	3.55191300
H	-0.08899100	9.19142900	3.84468500
C	3.72442400	2.50358000	2.81531400
H	3.27004600	1.76191700	3.47852700
H	4.19422500	1.97649000	1.97829000
H	2.91478000	3.11625600	2.39845400
C	6.39075300	12.37238200	10.07611600
H	6.65705500	13.32424000	9.60783400
H	6.77028500	12.36977900	11.10251000
H	5.29547100	12.32520100	10.13250600
C	4.49310000	7.70710000	4.07810000
C	3.65790000	6.85400000	4.55280000
H	-0.14203000	7.53614900	4.47689000
H	3.08990800	5.97663600	4.28391600
C	4.98140500	8.08823000	2.76182400
C	5.90673100	9.12580500	2.60731900
C	4.52246300	7.41900300	1.61412100
C	6.38362500	9.47410800	1.34826700
H	6.24629400	9.65950700	3.49127400
C	4.99478500	7.77031900	0.35794000
H	3.80431800	6.61111800	1.72603900
C	5.93199200	8.79555500	0.21952500
H	7.10645500	10.27901400	1.24803800
H	4.63555600	7.24125100	-0.52077900
H	6.30228700	9.06607000	-0.76524300

P:

104
Generated by Multiwfn

Sn	5.18760000	8.18280000	6.15830000
Si	2.65340000	7.30000000	6.85700000
Si	1.34193700	9.01411100	5.87084300
Si	1.32074500	5.32772600	6.81218100
Si	2.91651300	7.83487900	9.15769600
C	6.53810800	4.98095600	3.50921000
C	7.52202400	5.86568000	5.64354300
C	8.13752400	7.75257100	7.01787400
C	7.14460100	7.05181600	6.29998100
C	7.77355200	8.97278600	7.80140400
C	6.54137700	5.01252800	4.91417800
C	8.84944700	5.42223400	5.69065200
H	9.11434300	4.49804900	5.18119000
C	4.77028100	3.37285200	4.94382000
H	4.10295500	2.72307600	5.50811100
C	9.45851300	7.30062700	7.05360200
H	10.19987900	7.86470600	7.61574300
C	4.72132100	3.35884600	3.55077900
C	5.61783200	4.16689600	2.85062900
H	5.60492400	4.16065400	1.76152200
C	7.63690100	10.22507100	7.17878500
C	7.55537900	8.85424800	9.18866100
C	2.37508800	10.52900500	5.41660900
H	2.88268300	10.94841800	6.29198300
H	1.72132600	11.30249300	4.99294000
H	3.13790000	10.27935400	4.67109400
C	5.66424100	4.19107400	5.63797500
C	7.20345500	11.31939400	7.93324800
H	7.08127700	12.28282200	7.44088100
C	7.12735900	9.96839300	9.90716400
H	6.93842800	9.86679700	10.97470400
C	6.92530100	11.20565300	9.29226600
C	9.81804700	6.13764000	6.38337100
H	10.84553000	5.78447200	6.40919600
C	7.51889000	5.79916700	2.71195000
H	7.66673400	6.78914400	3.15250600
H	7.17175200	5.92715300	1.68296100
H	8.50153600	5.31216000	2.68202700
C	7.81719200	7.55567900	9.90498300
H	7.46945100	6.69359100	9.32836300
H	7.33149000	7.54589300	10.88417800
H	8.89401500	7.41441300	10.06008500
C	-0.01839200	9.59081100	7.05448900
H	-0.66923700	8.76855800	7.36803000
H	-0.64086400	10.34350000	6.55372200
H	0.40230900	10.05033000	7.95529400
C	1.92311000	4.01540100	8.03188600
H	2.91778900	3.64511100	7.76647000
H	1.23145400	3.16330400	8.02814300
H	1.97121400	4.40495200	9.05442800
C	5.67867600	4.17328000	7.14311200
H	6.69513300	4.26245100	7.53776700
H	5.23361200	3.24983800	7.52432900
H	5.10414900	5.01491700	7.55208400
C	1.33570500	7.55760400	10.16283000
H	0.49503200	8.13582100	9.76531300
H	1.50576100	7.87782200	11.19895500
H	1.04553000	6.50191500	10.18270900
C	8.00681900	10.42890900	5.73228800
H	9.03824300	10.79737900	5.65872800
H	7.35802600	11.17373400	5.26062900
H	7.96085800	9.49921600	5.15989000
C	1.16760900	4.50526800	5.11457600
H	0.81710700	5.20592400	4.34866800
H	0.43254800	3.69302300	5.18439900
H	2.11334700	4.06743500	4.77880400
C	-0.44058000	5.80447100	7.32226800
H	-0.47109400	6.33063500	8.28089100
H	-1.04404300	4.89236600	7.41796400
H	-0.91647500	6.44256900	6.57013500
C	3.40392000	9.65057200	9.35234500
H	4.27156300	9.91162800	8.73589000
H	3.67136700	9.84846000	10.39848300
H	2.57783300	10.31705500	9.08139900
C	4.27965800	6.76708700	9.90853400
H	4.01591200	5.70378700	9.88645500
H	4.46670300	7.05334700	10.95126500
H	5.21532900	6.89255500	9.35454000
C	0.51639400	8.39055000	4.28791200
H	1.26798500	8.08484300	3.55191300
H	-0.08899100	9.19142900	3.84468500
C	3.72442400	2.50358000	2.81531400
H	3.27004600	1.76191700	3.47852700
H	4.19422500	1.97649000	1.97829000
H	2.91478000	3.11625600	2.39845400
C	6.39075300	12.37238200	10.07611600
H	6.65705500	13.32424000	9.60783400

```
H       6.77028500     12.36977900     11.10251000
H       5.29547100     12.32520100     10.13250600
C       4.49310000      7.70710000      4.07810000
C       3.65790000      6.85400000      4.55280000
H      -0.14203000      7.53614900      4.47689000
H       3.08990800      5.97663600      4.28391600
C       4.98140500      8.08823000      2.76182400
C       5.90673100      9.12580500      2.60731900
C       4.52246300      7.41900300      1.61412100
C       6.38362500      9.47410800      1.34826700
H       6.24629400      9.65950700      3.49127400
C       4.99478500      7.77031900      0.35794000
H       3.80431800      6.61111800      1.72603900
C       5.93199200      8.79555500      0.21952500
H       7.10645500     10.27901400      1.24803800
H       4.63555600      7.24125100     -0.52077900
H       6.30228700      9.06607000     -0.76524300
```

Reaction of **1** with phenylacetylene to form **2'**:

T1_1:

```
   104
 Generated by Multiwfn
Sn      5.09644100      7.85758600      5.86049300
Si      3.37135600      7.93838600      8.01313700
Si      1.10129000      7.30137700      7.60586200
Si      3.71703400      7.36091300     10.28958700
Si      3.26258700     10.31196300      8.01625500
C       6.01327300      5.78202400      2.67719000
C       7.15797800      5.41805500      4.87754900
C       7.99593300      6.91269900      6.57145800
C       6.90829200      6.47719900      5.78564000
C       7.98275900      8.15474300      7.40424700
C       6.21375200      4.97064000      3.81008800
C       8.39875700      4.76755300      4.86904500
H       8.55290300      3.95595900      4.16143200
C       4.76769800      3.29742900      2.83311700
H       4.29162200      2.31992900      2.89717400
C       9.22890100      6.24873800      6.54335100
H      10.03888500      6.61984200      7.16788700
C       4.51028100      4.11086800      1.73387300
C       5.16339300      5.34160600      1.66281300
H       5.01429800      5.97434500      0.78894500
C       8.24853100      9.39850800      6.79453800
C       7.96824300      8.05432300      8.80686300
C       0.52783900      7.35535200      5.80062700
H       0.82540500      6.46101100      5.24800100
H       0.92524100      8.22645700      5.26861100
H      -0.56775900      7.42004700      5.78821300
C       5.62339200      3.69740200      3.86205900
C       8.44775000     10.51955700      7.60033200
H       8.64787900     11.47912700      7.12693600
C       8.19060300      9.19569100      9.57731000
H       8.19453200      9.10996300     10.66256700
C       8.42135800     10.43929500      8.99128400
C       9.42858900      5.15829200      5.71161200
H      10.38628100      4.64533900      5.68977800
C       6.70886000      7.10870700      2.52367700
H       6.20681300      7.89721100      3.10188000
H       6.71441300      7.42020100      1.47528500
H       7.74093100      7.06414400      2.88462000
C       7.77933300      6.71589100      9.46766700
H       6.99430400      6.14016900      8.96719500
H       7.51994400      6.82702200     10.52391000
H       8.69591800      6.11689100      9.40602700
C      -0.06651900      8.50145900      8.49861200
H       0.14342600      8.59467500      9.56712600
H      -1.09028400      8.12017100      8.38685200
H      -0.03457200      9.50176500      8.05479700
C       5.01619400      8.47688600     11.08718700
H       5.91629600      8.56824900     10.47689200
H       5.30114200      8.08306800     12.07133700
H       4.61473000      9.48578700     11.23315200
C       5.90876400      2.73100800      4.98109200
H       6.75792300      2.08508900      4.72306500
H       5.04425800      2.08488700      5.16275800
H       6.16302800      3.24292100      5.91094800
C       2.35831400     11.05672900      9.50542400
H       1.32787700     10.70266400      9.59430100
H       2.33360000     12.14878500      9.39509100
H       2.87563600     10.82975800     10.44334700
C       8.39085600      9.52589500      5.30046500
H       9.06639500      8.76131400      4.90184800
H       8.78090100     10.51186800      5.03481800
H       7.42946700      9.39627200      4.78652500
C       4.20385300      5.54959900     10.53377100
H       3.39014300      4.88938100     10.21405400
H       4.39549300      5.36051700     11.59780900
H       5.10184700      5.27358600      9.97266700
C       2.15099100      7.61939300     11.32824000
H       1.81767500      8.66190100     11.30401400
H       2.38823700      7.36865500     12.37094700
H       1.31575200      6.98671000     11.01440500
C       2.40155900     10.93031700      6.44596400
H       2.91677800     10.56447100      5.54991800
H       2.41061700     12.02743700      6.41707300
H       1.35880100     10.59955600      6.39232900
C       5.00400000     11.04491300      8.03666100
H       5.58942000     10.66064200      8.87910000
H       4.95477700     12.13813300      8.12502300
H       5.55980900     10.80883400      7.12213300
C       0.69419300      5.58092200      8.28294800
H       1.22666900      4.78998500      7.74871000
H      -0.38315600      5.40057500      8.17219400
C       3.54854700      3.68215500      0.65866000
H       3.49186600      2.59198300      0.58632700
H       3.83944600      4.08024200     -0.31829800
H       2.53697700      4.05024800      0.87300800
C       8.60651600     11.66797900      9.83770900
H       9.25839000     12.39629200      9.34607700
H       9.03308800     11.41953000     10.81406800
H       7.64126100     12.15967800     10.01558800
C       3.44268100      6.60954400      4.29262000
C       3.41487700      5.71445300      5.12796000
H       0.94105400      5.49155900      9.34570900
H       3.32897300      7.22557700      3.42883500
C       3.21202100      4.54922300      5.91577100
C       3.89540600      4.30455600      7.11383800
C       2.28107000      3.60404200      5.44474100
C       3.65100100      3.13692400      7.82544300
H       4.61390200      5.03522700      7.47174300
C       2.04151700      2.44543900      6.16762900
H       1.75906500      3.79254500      4.51187000
C       2.72589400      2.20776000      7.35985400
H       4.18910900      2.95365400      8.75044000
H       1.31737100      1.72406600      5.80039500
H       2.53680700      1.29858800      7.92299300
```

I1_1:

```
   104
 Generated by Multiwfn
Sn      5.05294100      7.78489100      5.87074300
Si      3.35643500      7.93905400      8.01257300
Si      1.09339600      7.28763000      7.59062300
Si      3.73325900      7.35900100     10.28370800
Si      3.26436400     10.31261100      8.00263800
C       6.00479300      5.78709000      2.66466700
C       7.16401000      5.43769100      4.86034300
C       7.99941600      6.92777100      6.56177800
C       6.91304100      6.48874200      5.77665200
C       7.98878200      8.16084400      7.40721700
C       6.21342200      4.98382200      3.80107600
C       8.40928000      4.79682200      4.83933400
H       8.56375500      3.99060300      4.12564500
C       4.75864100      3.30442200      2.85067000
H       4.28091200      2.32863200      2.92690800
C       9.23626900      6.27073800      6.52120800
H      10.04657600      6.64193900      7.14511700
C       4.49588600      4.10828100      1.74505600
C       5.14834600      5.33846400      1.65922000
H       4.99262800      5.96451600      0.78169400
C       8.23799400      9.41156500      6.80631400
C       7.98900900      8.04753900      8.80792700
C       0.54382700      7.32881300      5.77877500
H       0.87022400      6.44088200      5.23177600
H       0.93009400      8.20851600      5.25280700
H      -0.55264200      7.36857200      5.74983500
C       5.62124800      3.71258300      3.87008600
C       8.44513300     10.52534700      7.61959500
H       8.63358900     11.49040400      7.15242500
C       8.21552800      9.18291700      9.58691400
H       8.22851700      9.08720800     10.67132600
C       8.43665000     10.43215900      9.01019200
C       9.44035600      5.18896600      5.67952100
H      10.40181300      4.68356300      5.64928800
C       6.69189700      7.11748500      2.50403400
H       6.19208500      7.90118700      3.09016100
H       6.68348700      7.43081800      1.45626100
H       7.72830100      7.07794400      2.85299400
C       7.80383200      6.70399100      9.45961400
H       7.01785000      6.13108000      8.95714000
H       7.54636300      6.80817800     10.51716000
H       8.72039100      6.10557600      9.39206900
C      -0.06928600      8.50318800      8.46814400
H       0.13475100      8.59720800      9.53790900
H      -1.09625800      8.13260400      8.35091500
H      -0.02327900      9.50175600      8.02196000
C       5.03203400      8.48483600     11.06619700
H       5.91900700      8.59393500     10.43928500
H       5.34164500      8.08792100     12.04149700
H       4.61910400      9.48679100     11.22758700
C       5.91065400      2.76157100      5.00127600
H       6.77179800      2.12569700      4.75870000
H       5.05378000      2.10558000      5.18343900
H       6.14909600      3.28795800      5.92747000
C       2.35532500     11.06476600      9.48488800
H       1.32378500     10.71323200      9.57142000
H       2.33323700     12.15637400      9.37044000
H       2.86921800     10.84035200     10.42537400
C       8.34533300      9.55446300      5.31129300
H       9.01929400      8.80092200      4.88948000
H       8.71852100     10.54686900      5.04521500
H       7.37349300      9.41786000      4.81891500
C       4.22093800      5.55127300     10.54680700
H       3.41939400      4.88442400     10.21114900
H       4.38432900      5.37264300     11.61733200
H       5.13637100      5.27551900     10.01462900
C       2.16114500      7.62628300     11.30984000
H       1.82506000      8.66739600     11.27117300
H       2.39305000      7.38853100     12.35671600
H       1.32986600      6.98721700     10.99822200
C       2.41192800     10.91856800      6.42378700
H       2.92767300     10.53933400      5.53367200
```

H	2.42826800	12.01515500	6.38289100
H	1.36727300	10.59392900	6.37067600
C	5.00871200	11.03415400	8.02580300
H	5.57778200	10.67849000	8.89163300
H	4.96377000	12.13006500	8.07345300
H	5.57761300	10.76042200	7.13045400
C	0.67272100	5.57526300	8.27928400
H	1.16101300	4.77417000	7.71870300
H	-0.41270400	5.42433800	8.21326100
C	3.52846900	3.66932500	0.67887400
H	3.47276500	2.57848900	0.61643200
H	3.81361200	4.05904200	-0.30312500
H	2.51756600	4.03801000	0.89520500
C	8.62822700	11.65420100	9.86516300
H	9.28709700	12.38074600	9.38007500
H	9.05041600	11.39657900	10.84102900
H	7.66648100	12.15261100	10.04342200
C	3.55608900	6.65125300	4.41404800
C	3.50661500	5.76293500	5.27858900
H	0.96187200	5.47274600	9.33008600
H	3.37301200	7.08746700	3.45172900
C	3.23835600	4.56574800	6.00047000
C	3.86404800	4.26111000	7.21523000
C	2.31642600	3.65460000	5.45054600
C	3.57689100	3.06894400	7.86748900
H	4.57141800	4.97187600	7.63106500
C	2.03053900	2.47115200	6.11447700
H	1.84059100	3.88836800	4.50327500
C	2.66054700	2.17380000	7.32350800
H	4.07092300	2.83911100	8.80687500
H	1.31353500	1.77574000	5.68746200
H	2.43491600	1.24473200	7.83881500

T2_1:

104
Generated by Multiwfn

Sn	5.22020500	8.19646800	6.26567600
Si	2.73010800	7.25649600	6.96108600
Si	1.41422200	9.13477500	6.33760100
Si	1.39336000	5.26866500	6.96667900
Si	3.22439900	7.69320100	9.27698900
C	6.88546400	4.75685400	4.07553900
C	7.73056600	5.95396400	6.10118000
C	8.18302100	8.21973700	6.81300500
C	7.26402900	7.25603800	6.34378700
C	7.73457400	9.63532500	6.99820600
C	6.93091700	4.85403300	5.47851500
C	9.06887200	5.63341200	6.37551600
H	9.41366500	4.61987300	6.18065800
C	5.86318500	2.69123700	5.64216300
H	5.45895400	1.89079100	6.26005500
C	9.51401000	7.88756900	7.06870400
H	10.19996400	8.65499500	7.42109800
C	5.84955600	2.55942900	4.25507200
C	6.34774000	3.61212600	3.48810900
H	6.31851300	3.54124300	2.40232300
C	7.63190500	10.48364200	5.87670000
C	7.41407000	10.12471200	8.27860600
C	2.43639000	10.71956800	6.19060100
H	2.99435800	10.94388900	7.10438500
H	1.76160900	11.56087200	5.98355200
H	3.15420800	10.64899400	5.36614500
C	6.40726500	3.81667300	6.26615700
C	7.15409500	11.78276000	6.04905600
H	7.06554900	12.43221200	5.18019600
C	6.93958200	11.42957400	8.40849500
H	6.67122400	11.79640300	9.39770500
C	6.78756900	12.26769100	7.30425800
C	9.95420000	6.58527800	6.86403400
H	10.98745200	6.31655600	7.06683000
C	7.44399700	5.85412100	3.21107700
H	6.95143600	6.80876800	3.42446000
H	7.30783900	5.62326700	2.15138300
H	8.51362400	6.00297800	3.40127000
C	7.57008000	9.25404200	9.49485400
H	7.15214100	8.25677900	9.32668900
H	7.07419800	9.69981400	10.36080300
H	8.62891000	9.11111400	9.74141900
C	0.08453900	9.42421400	7.65407600
H	-0.55664400	8.54581800	7.78507400
H	-0.55293100	10.26367900	7.34824700
H	0.52272600	9.67154300	8.62647800
C	2.40031600	3.68242300	7.19175800
H	3.00429100	3.41029400	6.32389700
H	1.71079800	2.85433400	7.40172800
H	3.07305800	3.78268100	8.05181200
C	6.47836000	3.87952000	7.76733900
H	7.48773700	3.63559400	8.12202900
H	5.78141400	3.16988400	8.22222400
H	6.24930600	4.88230300	8.13605200
C	1.82982800	7.49362500	10.54921400
H	0.87287200	7.89978300	10.20990400
H	2.12649000	8.04078300	11.45348200
H	1.67483100	6.44869400	10.82975300
C	8.04989200	10.00860900	4.51035500
H	9.11363400	9.74338400	4.50052700
H	7.87757300	10.78401800	3.75984900
H	7.50560300	9.10700600	4.20965700
C	0.17874100	5.12733100	5.52544600
H	-0.61301500	5.87629900	5.64503300
H	-0.29555300	4.13775100	5.56201500
H	0.62824200	5.25549300	4.53992800
C	0.26695500	5.31858800	8.49107500
H	0.80868800	5.07964300	9.40951600
H	-0.50799400	4.55345300	8.34954800
H	-0.23413500	6.28087900	8.63084700
C	3.79510300	9.48754200	9.49487500
H	4.55577700	9.80453100	8.77410500
H	4.22393200	9.59463400	10.49993500
H	2.94612700	10.17626900	9.41908300
C	4.62544800	6.56185500	9.85958300
H	4.32649600	5.50819100	9.81713000
H	4.89807100	6.79728000	10.89637000
H	5.52289100	6.67877600	9.24282800
C	0.53480400	8.92192300	4.68004400
H	1.25090200	9.00658100	3.85741500
H	-0.20895000	9.72053200	4.56318600
C	5.33536100	1.30379700	3.60362300
H	4.53973000	0.84310700	4.19770300
H	6.13893700	0.56317000	3.50509100
H	4.94959300	1.50467100	2.59964900
C	6.21721500	13.65037000	7.46208400
H	5.12052700	13.61749600	7.44321900
H	6.54089100	14.30989300	6.65187400
H	6.51437000	14.09658700	8.41589300
C	4.55483500	8.11548500	4.18218100
C	3.71343500	7.16447900	4.43037200
H	0.02270400	7.95967700	4.58634500
H	4.99423400	8.47448800	3.25579000
C	3.03451200	6.12671500	3.69052700
C	2.17008000	6.45060600	2.62992500
C	3.27194800	4.77897300	3.98237800
C	1.55368900	5.44350200	1.89764500
H	1.99221000	7.49184100	2.38259200
C	2.65707100	3.77870300	3.24181000
H	3.96192600	4.53200100	4.78167000
C	1.78749800	4.10322000	2.20283700
H	0.88515000	5.70829900	1.08286300
H	2.85323200	2.73818400	3.48349200
H	1.29604500	3.31914700	1.63420000

P_1:

104
Generated by Multiwfn

Sn	4.01630100	7.12775100	3.89766600
C	6.23191000	7.24671000	1.03569300
C	6.42520200	5.51413900	2.86090100
C	6.40492900	5.43226500	5.27831200
C	5.83781800	5.87255900	4.07959500
C	5.77032500	5.86051300	6.55990500
C	5.76587200	6.06155500	1.63415500
C	7.56688800	4.71248000	2.83257800
H	8.02133100	4.44113300	1.88169700
C	3.92173800	6.01199200	0.06248000
H	3.02713500	5.52432200	-0.32094500
C	7.54970900	4.62911800	5.25028100
H	7.99251200	4.28642200	6.18352900
C	4.35474200	7.20507300	-0.52102900
C	5.51176900	7.80068400	-0.02751500
H	5.86975700	8.72546800	-0.47697900
C	6.28438800	6.96403400	7.26680600
C	4.63324500	5.18701400	7.04183800
C	4.60535400	5.42870600	1.12727400
C	5.64095100	7.38613600	8.43009600
H	6.03621300	8.24518000	8.97003900
C	4.02221100	5.63478200	8.21535100
H	3.14401100	5.10930600	8.58620500
C	4.50571600	6.73702400	8.91690600
C	8.12266600	4.26821900	4.03184800
H	9.01017700	3.64111200	4.01737600
C	7.50240100	7.91368900	1.48907800
H	7.75514800	7.65312000	2.51889900
H	7.42140000	9.00241000	1.40993300
H	8.34192200	7.59561900	0.85760300
C	4.08836500	3.98243100	6.32038600
H	3.67987400	4.25457600	5.33931300
H	3.29092200	3.51012800	6.89990300
H	4.87490800	3.24260000	6.13633400
C	4.13756500	4.11656800	1.69814600
H	4.91196000	3.34886300	1.58626600
H	3.23156100	3.77441700	1.19191100
H	3.92684400	4.19207200	2.77092600
C	7.52150800	7.67557900	6.78870200
H	8.40933300	7.04280800	6.90516300
H	7.68417200	8.59676900	7.35479300
H	7.45115500	7.92565000	5.72529600
C	3.57900000	7.82094800	-1.65275500
H	2.55906000	8.06794900	-1.33550200
H	3.49664600	7.12532800	-2.49554500
H	4.05415900	8.73859400	-2.00921800
C	3.80354500	7.24324800	10.14705200
H	4.51889400	7.57289200	10.90718900
H	3.16070200	6.47427500	10.58469900
H	3.17027500	8.10564000	9.90163500
C	5.23223500	8.95371300	3.94666400
C	4.87144400	10.25384100	3.86955000
Si	3.07320400	10.93021800	3.95896500
Si	1.81443500	10.42353600	2.01030100
Si	2.07890800	10.26791300	6.01756900
Si	3.05893500	13.32017600	4.08611700
C	1.25530300	8.63189900	1.80094300
C	2.86458200	10.85439100	0.49848600

```
C       0.24866600     11.48623500      2.01049700
C       2.37379800      8.47590700      6.53478900
C       0.20561300     10.50582600      5.92573800
C       2.80684800     11.30622000      7.42251800
C       3.20978500     14.20072200      2.41543300
C       1.39845000     13.88447100      4.80663500
C       4.42382400     14.00484600      5.20113700
H       0.56304200      8.58162100      0.94966500
H       2.09595300      7.96175200      1.58961700
H       0.73801100      8.24792600      2.68652000
H       2.29238700     10.67438300     -0.42045000
H       3.76045800     10.22279900      0.46453300
H       3.18228600     11.90205700      0.50156400
H      -0.34969400     11.25354200      1.12064900
H      -0.36946400     11.28598900      2.89269200
H       0.47396200     12.55689300      1.99316800
H       1.88253700      7.75859000      5.86916600
H       3.44219700      8.23004400      6.60235600
H       1.95723000      8.33263200      7.54115000
H      -0.22874400      9.82167100      5.18749200
H      -0.24773400     10.28110700      6.89933500
H      -0.07614300     11.52534600      5.64702800
H       2.38116100     10.97758500      8.37923400
H       3.89349000     11.16774200      7.47434600
H       2.60948100     12.37651400      7.31414500
H       2.40844700     13.91989100      1.72441100
H       4.16851600     14.00484900      1.92680700
H       3.13694400     15.28250200      2.58753900
H       0.54769500     13.51931800      4.22219900
H       1.26018700     13.55916900      5.84229300
H       1.36624300     14.98149600      4.79464100
H       5.40619500     13.91248900      4.72752500
H       4.46757000     13.49319600      6.16764100
H       4.23106300     15.06921000      5.38813900
H       6.31156600      8.77102900      3.91594900
C       5.96286700     11.25855200      3.75240400
C       6.07533000     12.05266100      2.60547200
C       6.91961200     11.40163200      4.76453800
C       7.11750900     12.96403200      2.47152000
H       5.34609100     11.93509700      1.80774500
C       7.95207300     12.32619200      4.63811400
H       6.83648000     10.78945200      5.65915200
C       8.05523400     13.11168300      3.49209200
H       7.19730400     13.56303600      1.56806200
H       8.67848500     12.43513500      5.43912900
H       8.86177700     13.83262100      3.39352900
```

Reaction of **1** with acetylene:

T1:

```
    94
 Generated by Multiwfn
Sn      5.65730300      8.34340800      6.47171000
Si      3.01047400      7.92025400      7.08147500
Si      1.30262100      7.14270400      5.61342600
Si      2.41433600      6.98892500      9.17888700
Si      2.61697000     10.24741200      7.37444300
C       6.63685300      5.32813100      3.30949900
C       7.15305500      5.41207500      5.76411800
C       7.92048900      6.69610300      7.65601800
C       6.98259300      6.51958900      6.61565900
C       7.78086700      7.84767900      8.60094700
C       6.22865500      5.10154100      4.63667700
C       8.21979900      4.52552400      5.96145600
H       8.32342000      3.67285600      5.29327900
C       4.14425200      4.19162100      3.82279300
H       3.17908900      3.73063400      4.02426900
C       8.97570900      5.80158000      7.84548500
H       9.67525000      5.96678200      8.66281000
C       4.52249500      4.42700300      2.50064600
C       5.77847500      4.98713800      2.26384300
H       6.09303500      5.17630200      1.23889800
C       8.42469700      9.07284000      8.33469200
C       7.04620000      7.68520800      9.78934900
C       1.62672300      7.30762300      3.75488100
H       2.26725800      6.50168600      3.38222800
H       2.07759700      8.26652200      3.48038500
H       0.66359700      7.22491900      3.23540100
C       4.98186800      4.51039400      4.89543600
C       8.25921400     10.13292300      9.22331600
H       8.74207200     11.08403800      9.00651900
C       6.91256500      8.76786900     10.66102900
H       6.34569500      8.63900100     11.58108900
C       7.49411900     10.00371700     10.38356300
C       9.12881700      4.71314200      6.99445600
H       9.94712300      4.01273900      7.13835500
C       7.96546700      5.96743200      3.01068900
H       8.11524900      6.85802900      3.63126600
H       8.02726700      6.25863900      1.95870900
H       8.79791100      5.28815200      3.22680300
C       6.46239100      6.34328800     10.13896700
H       5.93346800      5.90836200      9.28509000
H       5.77366500      6.41532700     10.98404200
H       7.25715400      5.63559700     10.40510700
C      -0.28724200      8.12694900      5.92022700
H      -0.60205700      8.10798200      6.96734200
H      -1.09241300      7.69062300      5.31436100
H      -0.17204300      9.17364600      5.61823900
C       3.19593300      7.99247600     10.57834900
H       4.25631100      8.18947200     10.39921700
H       3.09809200      7.46176000     11.53390800
H       2.69169500      8.96052400     10.67729900
C       4.55175300      4.21477500      6.30392000
H       5.37740300      3.80701500      6.89547600
H       3.72151300      3.50366800      6.32165800
H       4.22547200      5.13403700      6.80818900
C       1.00035600     10.70157100      8.25188000
H       0.11612000     10.27826500      7.76778200
H       0.89594600     11.79449800      8.25415700
H       1.00963100     10.36850800      9.29545900
C       9.27514800      9.23505800      7.10574900
H       9.69852700     10.24118900      7.05325000
H       8.68629500      9.06015300      6.19564200
H      10.09507500      8.50813900      7.09419900
C       2.86935200      5.16304400      9.38358400
H       2.34098200      4.54204000      8.65199200
H       2.57177700      4.82912600     10.38607600
H       3.94006700      4.97387000      9.26864800
C       0.53970600      7.07685000      9.45162300
H       0.16279200      8.10079500      9.36976700
H       0.31460200      6.71352400     10.46317800
H      -0.01421700      6.45340500      8.74286500
C       2.61935300     11.08586600      5.67554800
H       3.56207300     10.89384800      5.14945400
H       2.50505000     12.17208700      5.78126300
H       1.80400000     10.71877800      5.04223100
C       3.98954400     11.05785500      8.40434400
H       4.13019500     10.55578800      9.36815100
H       3.72086700     12.10348600      8.60432800
H       4.95249100     11.05060900      7.88199700
C       0.89703200      5.32014500      5.91937800
H       1.76421400      4.68177700      5.72775200
H       0.09192500      5.01031100      5.24031500
C       3.59075500      4.11171500      1.36145700
H       2.85757300      3.35179100      1.64643600
H       4.14054200      3.75387100      0.48555600
H       3.03547600      5.00728000      1.05431900
C       7.28084900     11.17881700     11.29673400
H       8.16083600     11.82876500     11.32294900
H       7.05473500     10.85520000     12.31675900
H       6.43539300     11.78543700     10.94736300
C       5.53247900      8.83543500      3.78186200
C       4.91493500      7.79436000      3.70290000
H       0.56260300      5.13425900      6.94472700
H       6.05189900      9.76039000      3.66966600
H       4.38357200      6.87210200      3.63972500
```

I1:

```
    94
 Generated by Multiwfn
Sn      5.56793500      8.26415500      6.56661400
Si      2.94254300      7.76102800      7.02697000
Si      1.27639700      6.83963600      5.59490100
Si      2.50771800      6.82864200      9.15944700
Si      2.38855700     10.06412700      7.27402900
C       6.63792200      5.56180600      3.24090500
C       7.19927500      5.46940100      5.68480300
C       7.95409100      6.68534800      7.62672200
C       7.00315800      6.52629300      6.59415400
C       7.82741600      7.79882400      8.61804400
C       6.27457800      5.19154200      4.54861600
C       8.29645700      4.60893900      5.82168600
H       8.41926500      3.79775600      5.10671600
C       4.23760900      4.21809800      3.69136900
H       3.31064600      3.67502500      3.86638400
C       9.03721500      5.81298900      7.75685500
H       9.74455300      5.96188900      8.57024600
C       4.56749100      4.59910700      2.39076000
C       5.77801800      5.26297200      2.18417400
H       6.05500400      5.56516100      1.17557800
C       8.41529200      9.05144700      8.35202600
C       7.16723300      7.58046900      9.83873000
C       1.56899600      7.05407400      3.73542800
H       1.91441200      8.05999500      3.47466500
H       0.61797400      6.87842900      3.21622300
H       2.28927000      6.32521200      3.34798700
C       5.07680700      4.49682700      4.77395100
C       8.26940900     10.08059400      9.27980500
H       8.71057500     11.05227100      9.06484100
C       7.04208800      8.63627700     10.74484100
H       6.52066400      8.46604300     11.68524000
C       7.57027600      9.89688900     10.47448000
C       9.21076000      4.77121500      6.85403600
H      10.05279300      4.09136000      6.95303200
C       7.91843200      6.30716400      2.98340200
H       7.99659600      7.18114800      3.64096200
H       7.97077700      6.64497200      1.94498300
H       8.79517700      5.68201100      3.18660200
C       6.63972500      6.21562200     10.18743800
H       6.17232800      5.73819700      9.32183600
H       5.91201100      6.26657000     11.00162500
H       7.45790700      5.55658000     10.50421000
C      -0.38070600      7.68797200      5.94730600
H      -0.66185200      7.62947500      7.00299900
H      -1.16208500      7.18856800      5.35928100
H      -0.36554100      8.74355600      5.65625000
C       3.32575400      7.88114800     10.50126200
H       4.37396600      8.09853000     10.27234600
H       3.28507800      7.36811400     11.47062800
H       2.80311200      8.83894100     10.60493800
C       4.69472500      4.06494900      6.16111500
```

```
H       5.55631100      3.67038100      6.70857500
H       3.91372400      3.30019100      6.13701600
H       4.31732700      4.92034800      6.73663800
C       0.77680700     10.38259300      8.21770400
H      -0.08009600      9.84633100      7.80135600
H       0.55435400     11.45715800      8.18567900
H       0.87855100     10.09689500      9.27054200
C       9.18039700      9.28066500      7.07831500
H       9.59623600     10.29102800      7.04831400
H       8.53047200      9.14963800      6.20260300
H       9.99883600      8.55994200      6.97208700
C       3.07507600      5.03221000      9.33437600
H       2.54768900      4.38591700      8.62397400
H       2.84551300      4.67707900     10.34745800
H       4.14788500      4.90574100      9.16719000
C       0.64941600      6.80745400      9.53166800
H       0.19876100      7.80101400      9.44970000
H       0.49710800      6.44994300     10.55866600
H       0.10752100      6.13247400      8.86110000
C       2.24079300     10.85638700      5.56006300
H       3.17074900     10.73137600      4.99315900
H       2.03846900     11.93121500      5.64941900
H       1.43120700     10.40669900      4.97441500
C       3.72153800     11.01745900      8.22717800
H       3.98021800     10.52465700      9.17112200
H       3.34596700     12.02236200      8.46068800
H       4.64344200     11.12160600      7.64621500
C       1.01365100      4.99275300      5.90477800
H       1.93053200      4.42023900      5.74210000
H       0.24837000      4.61278800      5.21551400
C       3.63512000      4.32413400      1.24195200
H       2.90596500      3.55041800      1.49840900
H       4.18558700      4.00179600      0.35237500
H       3.07693900      5.22863700      0.96860000
C       7.37571200     11.03888500     11.43421300
H       8.24132000     11.70839200     11.43711900
H       7.20969000     10.67888300     12.45364600
H       6.50094600     11.63662600     11.14828500
C       5.51530000      8.87040000      4.17384300
C       4.83727900      7.86635100      3.98318000
H       0.67210100      4.79823800      6.92675800
H       6.06984200      9.75785000      3.94896600
H       4.25785600      6.99061200      3.79560000
```

T2:

```
    94
 Generated by Multiwfn
Sn      5.18760100      8.18283000      6.15832700
Si      2.65336500      7.30001600      6.85698600
Si      1.20778300      8.81460300      5.74486400
Si      1.48508300      5.23952200      7.01607100
Si      2.88130800      8.09363800      9.08779100
C       6.31854100      4.58381600      3.95127700
C       7.51268200      5.80189700      5.79431900
C       8.18562300      7.91154500      6.75266600
C       7.15656100      7.07372800      6.27337600
C       7.82765400      9.24492300      7.32540200
C       6.51125700      4.80055800      5.32557600
C       8.85667700      5.40580300      5.77780000
H       9.10753800      4.41459300      5.40522100
C       4.97253000      3.00513400      5.81130600
H       4.45628400      2.38191200      6.53957500
C       9.52090900      7.50563700      6.73304900
H      10.28964400      8.17616600      7.11172700
C       4.76419000      2.77798000      4.45210800
C       5.43870600      3.58556500      3.53543500
H       5.28347200      3.42746300      2.46907700
C       7.67117000     10.36442500      6.48858700
C       7.60477700      9.36437800      8.70970500
C       2.14005300     10.35255100      5.16665000
H       2.59240300     10.88453200      6.01067500
H       1.44797300     11.03759600      4.66011300
H       2.93964500     10.09139700      4.46552700
C       5.83413200      4.00719800      6.26413800
C       7.22680200     11.56804800      7.03971300
H       7.09100500     12.42898500      6.38789900
C       7.15697300     10.58137500      9.22186800
H       6.96492200     10.66620200     10.29027800
C       6.94360600     11.68838900      8.39939700
C       9.85882500      6.25182600      6.23668000
H      10.89687300      5.93069100      6.21694600
C       7.05919200      5.40875300      2.93363200
H       7.08846400      6.46377600      3.22387900
H       6.59191500      5.32549200      1.94830400
H       8.10149900      5.07926800      2.84174000
C       7.87807000      8.20884900      9.63491200
H       7.53101900      7.26066000      9.21304400
H       7.39995000      8.36177000     10.60593300
H       8.95708600      8.09942100      9.80138400
C      -0.17987900      9.39530900      6.89483200
H      -0.77166000      8.55971500      7.28194400
H      -0.85526300     10.06135600      6.34257800
H       0.21518000      9.95384700      7.75001900
C       2.20551100      4.13665600      8.37100100
H       3.26207200      3.91976700      8.18899000
H       1.66290100      3.18354100      8.40929200
H       2.12386800      4.61112100      9.35507700
C       6.04396400      4.21606100      7.74021000
H       7.10944400      4.28053600      7.98565600
H       5.60254200      3.39972700      8.31852900
H       5.58788300      5.15676800      8.07374500
C       1.31199700      7.82530000     10.11312300
H       0.43493600      8.28727800      9.64888100
H       1.44586300      8.27382800     11.10601200
H       1.10136100      6.75990500     10.25385600
C       7.98955300     10.27531000      5.01906600
H       9.05057100     10.04626700      4.86514400
H       7.76063000     11.21644600      4.51299000
H       7.42658100      9.47371900      4.52539500
C       1.44758300      4.25102400      5.40603000
H       0.99307400      4.81974900      4.58684200
H       0.84663100      3.34464100      5.55520400
H       2.45157600      3.93918000      5.10087700
C      -0.31686500      5.60006400      7.47462500
H      -0.40249100      6.20144200      8.38481600
H      -0.84279600      4.65166600      7.64542700
H      -0.83548000      6.13059600      6.66907000
C       3.26824000      9.94451300      9.08869900
H       4.15122700     10.17689400      8.48179200
H       3.47226100     10.27714400     10.11476800
H       2.42675100     10.53037000      8.70323400
C       4.29723700      7.19892900      9.95968100
H       4.10378400      6.12352700     10.03962100
H       4.44021200      7.59847100     10.97179300
H       5.23745000      7.33187900      9.41457300
C       0.39830200      8.02600100      4.22423500
H       1.14990500      7.71987100      3.48908100
H      -0.25665500      8.76566300      3.74560000
C       3.84393200      1.68493800      3.97783800
H       3.31773200      1.21861100      4.81537200
H       4.40341300      0.90271300      3.45131900
H       3.09469800      2.07322300      3.27859700
C       6.39459300     12.96870200      8.96627300
H       6.63197500     13.82311200      8.32620500
H       6.79011800     13.16254000      9.96810600
H       5.30185500     12.91156100      9.05233600
C       4.54764300      7.64807100      4.08308000
C       3.65794700      6.85399500      4.55279800
H      -0.21402600      7.15027000      4.46936100
H       3.05084900      6.00368700      4.27738200
H       4.98787700      7.81837000      3.10785300
```

P:

```
    94
 Generated by Multiwfn
Sn      4.05154400      7.15135900      3.62925700
C       6.75415100      7.16359500      0.99945400
C       6.59512400      5.55941400      2.93577200
C       6.27792200      5.51580300      5.33235800
C       5.85929600      5.93519300      4.06768000
C       5.49835200      5.92557000      6.53796300
C       6.08993900      6.09238900      1.63508500
C       7.73568700      4.76641200      3.05867800
H       8.30041800      4.47845600      2.17440000
C       4.31975300      6.24680300     -0.01834400
H       3.37661400      5.87743400     -0.41705300
C       7.42431600      4.72216300      5.45505000
H       7.75057000      4.39806100      6.44142800
C       4.94872700      7.32234800     -0.63356000
C       6.17216800      7.75798500     -0.11639500
H       6.67718300      8.59961400     -0.58750700
C       5.85726500      7.08276400      7.25219200
C       4.40651200      5.14738600      6.96401400
C       4.87332500      5.61061200      1.10167800
C       5.12270100      7.43739200      8.38503900
H       5.40396100      8.33282300      8.93656900
C       3.69693400      5.53440600      8.10119800
H       2.85416100      4.92856600      8.42966300
C       4.04066500      6.67628100      8.82436800
C       8.14481300      4.34642700      4.32348500
H       9.03011900      3.72490700      4.42808000
C       8.07552600      7.67007700      1.51186800
H       8.10726500      7.68311700      2.60505600
H       8.27480800      8.67772000      1.13745800
H       8.89480000      7.01994700      1.18127200
C       4.01383200      3.90478200      6.21058000
H       3.72617200      4.14333600      5.17930500
H       3.17126000      3.40370000      6.69396300
H       4.84996200      3.19945900      6.14661300
C       4.22572500      4.34602900      1.61114300
H       4.60690600      3.49048300      1.03855100
H       3.14102800      4.37962700      1.47617300
H       4.44642100      4.15485000      2.66312500
C       7.02383300      7.92589100      6.81280800
H       7.96404400      7.36558300      6.87461200
H       7.11357300      8.81915200      7.43671000
H       6.90962700      8.24101400      5.76961600
C       4.34602500      7.99222900     -1.83741300
H       3.29159700      7.72719800     -1.95197800
H       4.87098200      7.69092400     -2.75217500
H       4.42147100      9.08159500     -1.76096600
C       3.23739300      7.10075800     10.02310400
H       3.84428200      7.68180000     10.72380900
H       2.82387700      6.23717300     10.55268600
H       2.39310700      7.73291700      9.71861600
C       5.24290400      8.99643500      3.57640700
C       4.79176300     10.24903400      3.77656200
Si      3.02987200     10.90289800      4.08446500
Si      1.50648600     10.27901300      2.37133000
Si      2.22055100     10.28148700      6.21329100
Si      3.36025000     13.24353200      3.98183100
C       0.40366000      8.80850700      2.81066000
```

```
C       2.47457700     9.88211200     0.79951000
C       0.33344800    11.72257200     2.02138000
C       2.06487800     8.40894700     6.38160600
C       0.50467600    11.03455100     6.46769700
C       3.38150600    10.88419200     7.57714000
C       3.89211100    13.70440600     2.22401100
C       1.84412800    14.26321400     4.46137100
C       4.76887400    13.74380600     5.14383700
H      -0.28526200     8.60836400     1.97987700
H       0.98732100     7.90137700     2.99773100
H      -0.19864500     9.01698100     3.70213100
H       1.78834000     9.64546300    -0.02345900
H       3.13712900     9.02090300     0.94514700
H       3.09729900    10.73046200     0.49328300
H      -0.37134000    11.43586500     1.23055200
H      -0.25110700    11.98110800     2.91169600
H       0.86339600    12.62188100     1.69183700
H       1.44079300     7.97244600     5.59509600
H       3.04720800     7.91931800     6.35930600
H       1.61593800     8.16013900     7.35193100
H      -0.19931400    10.68203500     5.70535800
H       0.10739900    10.74800500     7.44941300
H       0.53075100    12.12810100     6.41959800
H       3.08305800    10.45683400     8.54321300
H       4.41294800    10.57217700     7.37689700
H       3.36743800    11.97536500     7.66892000
H       3.10172500    13.50602700     1.49241900
H       4.77701600    13.13208400     1.92200700
H       4.14546300    14.77060700     2.16837400
H       0.96075400    14.00184100     3.87070200
H       1.59665500    14.12542200     5.51956200
H       2.05285300    15.32896200     4.30435400
H       5.69496500    13.20153300     4.92499600
H       4.51009300    13.55523600     6.19100500
H       4.97502400    14.81624900     5.03472500
H       5.54857700    11.04528000     3.77002700
H       6.31987000     8.87741600     3.42114400
```

Reaction of 1 with trimethylsilylacetylene

T1:

```
   106
 Generated by Multiwfn
Sn      5.22216500     7.70238500     5.67553300
Si      3.70860900     7.81143800     7.92968100
Si      1.79746700     6.40736800     7.91511500
Si      4.38349000     7.91350200    10.19232900
Si      2.89330000     9.97235500     7.40141600
C       5.07559000     4.04308700     4.15624900
C       6.88231700     4.87297000     5.68282600
C       8.13698800     6.89713400     6.07326200
C       6.89744800     6.25206100     5.92843800
C       8.13378000     8.37659200     6.28860200
C       5.59690700     4.15266700     5.45577000
C       8.08389800     4.15768200     5.62648800
H       8.05631200     3.08551100     5.44123800
C       3.76945700     2.81596900     6.29652500
H       3.26564700     2.33104700     7.13087100
C       9.33217400     6.18077700     6.01331600
H      10.28050200     6.70076500     6.13503200
C       3.22695900     2.70646700     5.01509300
C       3.89358500     3.32431200     3.95818500
H       3.49324800     3.23669200     2.94933000
C       8.13028500     9.25649700     5.18576700
C       8.16285000     8.89361100     7.59829500
C       1.38556300     5.79629800     6.17859500
H       2.21133600     5.19316500     5.78420200
H       1.20456800     6.63216900     5.49426600
H       0.48504300     5.16901600     6.20114700
C       4.94208000     3.53222000     6.53425800
C       8.08795300    10.63256800     5.41559000
H       8.07285000    11.30977300     4.56334800
C       8.13506600    10.27549000     7.78582200
H       8.16524900    10.67353800     8.79841900
C       8.07755900    11.15855100     6.70709800
C       9.30384200     4.80560500     5.79505500
H      10.23124800     4.24131400     5.74634400
C       5.80032400     4.64887100     2.98357600
H       6.02047100     5.70930700     3.15085400
H       5.20459100     4.56585400     2.07095700
H       6.76192700     4.14806500     2.81822700
C       8.27799800     7.95693400     8.76854800
H       7.51061200     7.17788000     8.71793500
H       8.18100700     8.48915800     9.71746000
H       9.24693500     7.44412400     8.76013100
C       0.26799500     7.30253900     8.58233300
H       0.43013400     7.68037800     9.59689600
H      -0.57598700     6.60084000     8.61242400
H      -0.02052600     8.14791700     7.94901900
C       5.42044300     9.46801000    10.49358000
H       6.17453400     9.61782900     9.71681400
H       5.92790400     9.41249100    11.46497400
H       4.77376100    10.35299300    10.50211000
C       5.51189300     3.63739800     7.92241800
H       6.54642100     3.27875900     7.95494700
H       4.92248600     3.05797600     8.63791600
H       5.53116100     4.68373100     8.25262500
C       1.85003000    10.78997400     8.75392200
H       0.99869300    10.17168700     9.05538900
H       1.46062700    11.74849400     8.38700300
H       2.45196700    10.99463500     9.64605700
C       8.23897100     8.72697200     3.78208600
H       9.25584700     8.36312900     3.58884500
H       8.00873700     9.50568100     3.04989600
H       7.56518400     7.87940600     3.61664900
C       5.31530900     6.37801600    10.78426200
H       4.69394000     5.48339200    10.66441300
H       5.55330900     6.48215300    11.85074900
H       6.25109700     6.20756100    10.24453700
C       2.86975400     8.07081400    11.32288400
H       2.24094700     8.92105900    11.04005300
H       3.20885900     8.22894000    12.35539900
H       2.24708000     7.17053000    11.31035400
C       1.84575600     9.87547900     5.82621600
H       2.38593600     9.36170800     5.02237000
H       1.58320800    10.88142100     5.47439100
H       0.91490900     9.32523700     6.00320100
C       4.34679400    11.14574300     7.07505300
H       4.97569000    11.24646300     7.96708200
H       3.97565300    12.14334500     6.80560900
H       4.98970100    10.79652700     6.25746800
C       2.06776400     4.88715200     9.00591600
H       2.95517200     4.33014700     8.69346600
H       1.20119700     4.21771900     8.92731200
C       1.93555300     1.96861500     4.78913400
H       1.83956700     1.11743200     5.47011700
H       1.85995900     1.60236600     3.76116600
H       1.07721200     2.62862100     4.96910300
C       7.97810200    12.64148100     6.93121200
H       8.46544500    13.20063500     6.12696800
H       8.42960600    12.93223800     7.88418600
H       6.92524600    12.95048800     6.95648100
C       3.48637700     7.65380400     2.82958700
C       2.92525400     6.63292800     3.17697200
H       2.19003700     5.15823200    10.05957800
H       2.47208500     5.73429800     3.52905800
Si      4.16129800     9.09586300     1.87925800
C       4.73149700    10.49080100     2.99114400
H       5.09124100    11.31985200     2.36885700
H       3.91416400    10.86590700     3.61642000
H       5.54398400    10.17605700     3.65379500
C       5.57970200     8.44109400     0.83959900
H       5.22974400     7.66070200     0.15514600
H       6.02128100     9.24770000     0.24231300
H       6.36800400     8.01269500     1.46695800
C       2.75649400     9.69027000     0.78510400
H       1.91711500    10.04835500     1.39119200
H       3.09222800    10.51619800     0.14679400
H       2.39161400     8.88424700     0.13993400
```

I1:

```
   106
 Generated by Multiwfn
Sn      5.27415400     8.02463500     5.61676500
Si      3.60025900     8.03077900     7.77662200
Si      1.46239000     7.01317100     7.58591700
Si      4.32491100     7.70194900    10.01728300
Si      3.15186700    10.36231500     7.71112800
C       5.36900400     4.00234900     3.75579600
C       6.93481500     5.10180300     5.37802600
C       8.16628300     7.08375000     5.98127300
C       6.91999000     6.46976800     5.72170500
C       8.25264400     8.52108900     6.38509400
C       5.71300700     4.29459800     5.08604600
C       8.14655600     4.40157600     5.29688700
H       8.12142500     3.34615300     5.03287900
C       3.85259300     2.95053600     5.84125400
H       3.27192800     2.52924800     6.66018000
C       9.36463900     6.37102600     5.89550600
H      10.30145100     6.88360900     6.10520400
C       3.48362700     2.66669800     4.52539400
C       4.25724300     3.19993500     3.49551600
H       3.98528500     2.99334300     2.46174600
C       8.38057200     9.53224400     5.41572100
C       8.30018000     8.85078000     7.75162000
C       0.67516400     7.06120100     5.85919200
H       0.99907900     6.23746500     5.21738800
H       0.89026600     7.99610400     5.33172600
H      -0.41303800     6.98353000     5.98008700
C       4.95545600     3.75487400     6.13733700
C       8.46489200    10.86231800     5.82753200
H       8.54390100    11.64419100     5.07418300
C       8.39919900    10.19095800     8.12649400
H       8.43470800    10.44412200     9.18480700
C       8.46004500    11.21147700     7.17765700
C       9.35950200     5.02632100     5.55030300
H      10.29018500     4.46941900     5.48259100
C       6.16213800     4.58328300     2.61907100
H       6.32943000     5.65473400     2.77661300
H       5.64357400     4.44276700     1.66662200
H       7.15261400     4.11960800     2.54264700
C       8.29194300     7.76146100     8.78918600
H       7.41153000     7.12026500     8.67492500
H       8.29604400     8.17444000     9.80032000
H       9.16709300     7.11139600     8.67736500
C       0.19910400     7.90127600     8.68420900
H       0.52604100     8.02007500     9.71970800
H      -0.73510500     7.32426800     8.68347000
H      -0.02436700     8.89523900     8.28080100
C       5.51267500     9.09564500    10.49852100
H       6.25342000     9.29638000     9.72066300
H       6.04447200     8.84119600    11.42410000
```

H	4.95753300	10.02356500	10.67348200
C	5.29483300	4.07658800	7.56297700
H	6.35310500	3.89321600	7.77806800
H	4.68608500	3.49084700	8.25676900
H	5.11232300	5.14014600	7.76173200
C	2.26667300	11.05146100	9.23912000
H	1.32700000	10.53063100	9.44736600
H	2.03445700	12.11048700	9.06661900
H	2.89203900	10.99064500	10.13558900
C	8.47342600	9.18186000	3.95669900
H	9.45697300	8.75222300	3.72770200
H	8.33354100	10.06617000	3.32895800
H	7.72853900	8.42968400	3.67777500
C	5.12893400	6.03895600	10.42561600
H	4.43600900	5.21109400	10.24086600
H	5.38046600	6.03424500	11.49430200
H	6.04528700	5.83967200	9.86396400
C	2.85931700	7.82483900	11.21202800
H	2.30352500	8.75871800	11.08660100
H	3.24431700	7.79506500	12.24005500
H	2.16185100	6.98897100	11.09229300
C	2.05144500	10.73290500	6.21663700
H	2.47415400	10.29955300	5.30424000
H	1.95434500	11.81554900	6.06634900
H	1.04623500	10.31705300	6.34968200
C	4.74863200	11.36025100	7.52030000
H	5.41922800	11.21196200	8.37370000
H	4.51085100	12.43011000	7.45597500
H	5.30238000	11.08029300	6.61664000
C	1.56061200	5.20607200	8.13642500
H	2.26365300	4.64231600	7.51290200
H	0.57750700	4.72434600	8.05993700
C	2.26470000	1.83629400	4.22777700
H	2.08675500	1.09503900	5.01262600
H	2.36350500	1.31387300	3.27176200
H	1.37001000	2.46862100	4.16573700
C	8.49475000	12.65375300	7.60070600
H	9.02760900	13.27131400	6.87156800
H	8.97511800	12.77174500	8.57659300
H	7.47473400	13.05013100	7.68589000
C	3.79435400	7.13484000	3.78992600
C	3.53655400	6.24438100	4.60247000
H	1.89979200	5.12597500	9.17558700
H	3.26738500	5.42358000	5.23262100
Si	3.71366700	8.07930100	2.18519600
C	2.73312900	9.65095900	2.45524800
H	2.56232200	10.14817900	1.49283900
H	1.75913900	9.44837400	2.91278100
H	3.28058600	10.34566400	3.10089700
C	5.42930800	8.49790700	1.56944100
H	6.05397400	7.60779000	1.44444200
H	5.35174400	8.99707100	0.59590700
H	5.93298500	9.17946600	2.26194600
C	2.83334800	6.91669700	1.00312600
H	1.83071600	6.67210300	1.36983100
H	2.73474500	7.37638800	0.01281100
H	3.39337200	5.98161100	0.89372800

T2:

106

Generated by Multiwfn

Sn	5.18760000	8.18280000	6.15830000
Si	2.65340000	7.30000000	6.85700000
Si	1.33043600	8.98433500	5.83159300
Si	1.29873300	5.34382500	6.86453800
Si	2.79960400	7.94146900	9.14478600
C	6.48199800	4.44592100	3.82636300
C	7.47175900	5.74932600	5.74296500
C	8.14089100	7.76360100	6.89607800
C	7.11990600	7.00015600	6.28752700
C	7.79157700	9.02447300	7.62278800
C	6.46986600	4.79121000	5.19167200
C	8.80460900	5.31649700	5.78047200
H	9.05076200	4.33667800	5.37722600
C	4.68653400	3.19915000	5.54944800
H	4.01124000	2.68664800	6.23227400
C	9.46632200	7.32564300	6.91108500
H	10.22673700	7.94001000	7.38886700
C	4.64985900	2.88303200	4.19427300
C	5.55998000	3.51723400	3.34750300
H	5.56400200	3.26791200	2.28718900
C	7.70606000	10.25821400	6.95612800
C	7.52948700	8.95584500	9.00528000
C	2.34698200	10.52170600	5.41807600
H	2.75293600	10.98160700	6.32578800
H	1.71112100	11.25990700	4.91191900
H	3.18644800	10.28228200	4.75883300
C	5.57793700	4.14566700	6.06274100
C	7.28368600	11.38747800	7.66216500
H	7.19903800	12.33666900	7.13596100
C	7.10983200	10.10339800	9.67536200
H	6.88710500	10.04161100	10.73940600
C	6.96112200	11.32452100	9.01559000
C	9.80171400	6.10248200	6.34284000
H	10.83022400	5.75184400	6.35985800
C	7.48914200	5.04013200	2.87805100
H	7.61519400	6.11271900	3.04526900
H	7.19182400	4.87696400	1.83865500
H	8.47698800	4.58483300	3.01348700
C	7.73826300	7.67324300	9.76556900
H	7.34352400	6.80912800	9.22243000
H	7.26562300	7.71971800	10.75011700
H	8.80925100	7.48529700	9.91154100
C	-0.08244900	9.56138800	6.95279100
H	-0.74149800	8.74162700	7.25406300
H	-0.68730300	10.30009000	6.41078200
H	0.29594200	10.04334200	7.86039900
C	1.82741600	4.10849000	8.19527400
H	2.85607600	3.76588000	8.05529600
H	1.16892300	3.23086000	8.16907000
H	1.75601400	4.55144300	9.19470300
C	5.58486500	4.42313500	7.54202300
H	6.59246800	4.63992300	7.90751300
H	5.18673900	3.56876000	8.09717100
H	4.96281500	5.29475700	7.78238100
C	1.17261300	7.72180100	10.09125500
H	0.34978600	8.27850700	9.63191100
H	1.30101500	8.09270400	11.11649900
H	0.87878200	6.66871300	10.15244200
C	8.09359800	10.38116000	5.50768700
H	9.18259600	10.47893000	5.41016200
H	7.63528100	11.26267900	5.05055800
H	7.80702300	9.49698700	4.93251500
C	1.22709500	4.43404900	5.20655100
H	0.85224400	5.08010600	4.40427800
H	0.53679200	3.58545100	5.29853000
H	2.20253300	4.03655400	4.90877700
C	-0.48933900	5.82839000	7.26443900
H	-0.56998500	6.39720200	8.19540500
H	-1.08634400	4.91355600	7.37541100
H	-0.93751700	6.42389000	6.46231700
C	3.29197900	9.76381200	9.27361200
H	4.19373200	9.98393100	8.69059400
H	3.50473600	10.01440400	10.32103000
H	2.49076100	10.42328000	8.92280200
C	4.11174600	6.93093400	10.05362500
H	3.86901200	5.86258400	10.06321200
H	4.19732400	7.27163900	11.09351600
H	5.09157100	7.05045600	9.58145600
C	0.55102100	8.34334700	4.22959600
H	1.31635800	8.01910800	3.51709700
H	-0.03925900	9.13954700	3.75816800
C	3.66787600	1.88013400	3.65095900
H	3.11315900	1.39084200	4.45648000
H	4.17580300	1.10703000	3.06398800
H	2.93975500	2.36327400	2.98787400
C	6.43611300	12.53001900	9.74567600
H	6.72169600	13.45792500	9.24216900
H	6.80512200	12.56361700	10.77544700
H	5.33982900	12.50107600	9.79287300
C	4.49310000	7.70710000	4.07810000
C	3.65790000	6.85400000	4.55280000
H	-0.11677100	7.49544900	4.41638300
H	3.06438500	5.98844500	4.30072000
Si	4.95205900	8.26377900	2.35641100
C	6.73047500	8.86407700	2.28188900
H	7.05292800	8.94535600	1.23699700
H	6.81268900	9.85849800	2.73364900
H	7.43081100	8.20259400	2.80001800
C	4.62716700	6.85060700	1.15953900
H	4.91511200	7.12901800	0.13886600
H	5.17796500	5.94822300	1.44078900
H	3.55939200	6.60211700	1.15465600
C	3.83633000	9.71094600	1.90685400
H	4.03212400	10.01535900	0.87110100
H	2.77674600	9.44613900	1.98865700
H	4.02250200	10.57612200	2.55176600

P:

106

Generated by Multiwfn

Sn	4.45172500	7.56451800	3.70492900
C	6.83044900	6.41065500	0.58293900
C	6.47530700	5.32547100	2.85168600
C	6.28106800	5.45850900	5.26423900
C	5.94516800	5.94619100	3.99693500
C	5.55334900	5.94980000	6.47813700
C	5.97438100	5.80702400	1.52914400
C	7.40022500	4.28808300	2.98054000
H	7.80594400	3.80601400	2.09393500
C	4.08367100	6.33191700	0.09016900
H	3.01597300	6.26312200	-0.11199100
C	7.22392100	4.43032500	5.38478600
H	7.49322500	4.06235000	6.37299100
C	4.90830600	7.00518100	-0.80286400
C	6.28244000	6.99678600	-0.55692700
H	6.95048500	7.48219200	-1.26697900
C	6.00841900	7.04848200	7.22640400
C	4.39637100	5.25643300	6.89476900
C	4.59169000	5.70472000	1.23483100
C	5.30173500	7.44135200	8.36743300
H	5.66283900	8.29255200	8.94142800
C	3.73002400	5.66849000	8.04609600
H	2.83822300	5.12877000	8.36066400
C	4.16405300	6.76381500	8.79413000
C	7.79204700	3.86154600	4.24836200
H	8.51948800	3.06059900	4.34942700
C	8.32620400	6.41779500	0.74943700
H	8.62489100	6.43433700	1.79762100
H	8.76907500	7.27895800	0.24109900
H	8.75985300	5.51329200	0.30456000

```
C       3.85460500      4.09920100      6.09908600
H       3.39450100      4.45664400      5.16895400
H       3.08895500      3.56258600      6.66549300
H       4.64096800      3.39413500      5.81298100
C       3.66854900      4.78218900      1.99707500
H       3.51759000      3.87149600      1.40314700
H       2.68596700      5.23788300      2.15500500
H       4.07907900      4.48371000      2.96160100
C       7.24038300      7.80345400      6.81797500
H       7.58788900      8.45259800      7.62632200
H       7.03358500      8.43293400      5.94414700
H       8.04969300      7.12432800      6.53400800
C       4.34741400      7.72394700     -1.99780100
H       3.31008000      7.43399900     -2.18570600
H       4.93612400      7.51826300     -2.89770900
H       4.36723700      8.80875100     -1.83563000
C       3.40422300      7.20150900     10.01591400
H       3.90636500      8.02970000     10.52333800
H       3.29330600      6.37685100     10.72879200
H       2.39422800      7.53321600      9.74639600
C       5.73407800      9.36789700      3.75589600
C       5.01696200     10.43620600      4.18852600
Si      3.14145900     10.82964000      4.35093200
Si      1.88203100     10.24689900      2.41602600
Si      2.03027700     10.09273900      6.30391200
Si      3.31608000     13.19746000      4.39612500
C       0.79677300      8.71077700      2.60971600
C       3.03822200     10.02190600      0.93685500
C       0.68418400     11.65527600      2.00366900
C       1.80954300      8.22160100      6.37541100
C       0.30474700     10.86848900      6.30393400
C       2.96041300     10.62702800      7.85876800
C       4.08170500     13.74791000      2.75281700
C       1.69684700     14.13182000      4.67153800
C       4.50279800     13.73988500      5.77001300
H       0.28445000      8.50945000      1.65999800
H       1.37928000      7.82587000      2.88342500
H       0.02943700      8.86674400      3.37663700
H       2.44821200      9.84593500      0.02804100
H       3.71655300      9.17016100      1.06387600
H       3.65156500     10.91650800      0.77915500
H       0.09482000     11.38375200      1.11854200
H      -0.01551900     11.83442500      2.82773300
H       1.20090200     12.59601100      1.78939500
H       1.29752700      7.83895600      5.48646400
H       2.76738200      7.69780800      6.46578900
H       1.20946400      7.96224600      7.25809400
H      -0.26244300     10.56866700      5.41525400
H      -0.25604500     10.53677200      7.18683800
H       0.34809400     11.96190200      6.32107000
H       2.46033300     10.23269500      8.75259800
H       3.98540000     10.23893500      7.84833800
H       3.00902700     11.71703700      7.95009000
H       3.41300500     13.55956200      1.90614100
H       5.01941200     13.21294600      2.56049900
H       4.30516400     14.82197200      2.77564600
H       0.89834800     13.79780200      4.00280400
H       1.34787600     14.01154300      5.70285900
H       1.85857600     15.20325400      4.49864000
H       5.48612900     13.26437600      5.68578600
H       4.10082100     13.50672400      6.76161500
H       4.65301100     14.82584600      5.71551500
H       5.59259900     11.34472500      4.41921800
Si      7.53060600      9.68944200      3.28818000
C       8.15947700     11.29084900      4.07321400
H       9.22114700     11.41745000      3.82713700
H       7.62878600     12.17772100      3.70945800
H       8.07329900     11.26475500      5.16620100
C       7.52714800      9.93999900      1.41826500
H       6.94317100     10.83432400      1.16957300
H       8.54478200     10.07606800      1.03256100
H       7.07168700      9.09129500      0.89848900
C       8.74743400      8.33133600      3.77622600
H       9.18212700      8.54799300      4.75766100
H       8.27596700      7.34512400      3.83108200
H       9.56978000      8.28591700      3.05293800
```

Reaction of 1 with 3-hexyne:

T1:

```
   106
 Generated by Multiwfn
Sn      5.24762500      7.84471900      5.69912100
Si      2.88160800      7.92969200      7.00008600
Si      0.91199500      8.17573800      5.65680700
Si      2.53553600      6.47167000      8.82884900
Si      3.00971100     10.19375100      7.77066900
C       7.15856100      5.83990300      3.50060100
C       7.24516300      5.43571500      5.97856800
C       7.50080200      6.82792500      7.92850800
C       6.79443700      6.53748500      6.73973600
C       7.32754500      8.05242500      8.76820900
C       6.71551500      5.10382400      4.62058900
C       8.28887400      4.61892200      6.42583600
H       8.60626900      3.78384300      5.80517100
C       5.59095200      3.56163500      3.13512000
H       4.99908300      2.65919500      2.99188300
C       8.54654400      5.99665400      8.35540800
H       9.07912200      6.25812300      9.26703300
C       5.95511400      4.32090400      2.02371900
C       6.75692700      5.44112600      2.22447300
H       7.10590300      6.00885300      1.36387500
C       7.87677900      9.27221800      8.32788800
C       6.85732000      7.94084300     10.09076100
C       1.29381100      9.06609700      4.03572900
H       2.01600700      8.48270000      3.45712600
H       1.70523300     10.06721700      4.18518000
H       0.37499800      9.15839900      3.44209900
C       5.97059500      3.92272700      4.42506100
C       7.91399300     10.35944500      9.20080700
H       8.34532800     11.29721700      8.85480200
C       6.88444400      9.06028900     10.92386400
H       6.50131900      8.97086000     11.93919800
C       7.40978900     10.27889200     10.49685700
C       8.93385500      4.88517500      7.62363800
H       9.75147900      4.25644000      7.96549600
C       8.12339000      6.98659200      3.64366300
H       7.62620600      7.89175100      4.01692600
H       8.57443800      7.22865800      2.67775900
H       8.91946700      6.74516600      4.35542200
C       6.39094000      6.62107200     10.64611500
H       5.77394100      6.07332600      9.92984400
H       5.81550300      6.76885500     11.56332800
H       7.24428000      5.97613800     10.88704700
C      -0.35958100      9.18902500      6.63510500
H      -0.66611700      8.65553100      7.54195800
H      -1.25152800      9.31802100      6.00808800
H      -0.00978800     10.18084900      6.93053700
C       3.01089500      7.30586600     10.45872100
H       3.91823100      7.91188300     10.36984700
H       3.17571500      6.55317700     11.23944100
H       2.19806100      7.96207400     10.78679900
C       5.63524600      3.01352000      5.57927500
H       6.48490600      2.36098200      5.81491400
H       4.78548500      2.36996600      5.33441700
H       5.40760600      3.57178800      6.49190000
C       1.83892600     10.52518300      9.22453700
H       0.88062600     10.00499300      9.14593400
H       1.63771600     11.60258700      9.28550700
H       2.31427000     10.21924300     10.16205000
C       8.47118100      9.41353200      6.95141200
H       9.04045000     10.34325300      6.87039100
H       7.69766900      9.43225500      6.17057300
H       9.13109800      8.57279200      6.71262100
C       3.58259300      4.91794000      8.56102500
H       3.14117400      4.29553100      7.77395300
H       3.63601400      4.31589900      9.47691200
H       4.60230500      5.16816800      8.24807700
C       0.73886300      5.90500200      9.01234200
H       0.05251000      6.75332900      9.10729400
H       0.63912900      5.28922600      9.91566200
H       0.41605700      5.30020500      8.15863500
C       2.65138500     11.44798800      6.39709800
H       3.31683400     11.29077200      5.54047300
H       2.85513000     12.44981400      6.79765600
H       1.62061800     11.43583900      6.03431400
C       4.74055600     10.63312400      8.35690300
H       5.11655000      9.93992600      9.11396700
H       4.74336000     11.64193500      8.79109000
H       5.45723900     10.63537900      7.52695000
C      -0.05186200      6.61276200      5.18941800
H       1.03609400      3.47263400      4.39389200
H       0.41002000      6.07601000      4.35689400
H      -1.04819800      6.94080500      4.86509600
C       5.47752300      3.94268000      0.65027200
H       5.49676400      2.85785000      0.50529900
H       6.08503000      4.41067000     -0.12958400
H       4.43965200      4.27046300      0.50932400
C       7.39299900     11.48530700     11.39376000
H       8.22401100     12.16029700     11.16853000
H       7.44912300     11.20091900     12.44871600
H       6.46294000     12.05147300     11.25427000
C       2.76470900      6.08482700      2.55968900
C       2.77513800      5.49769400      3.61737200
C       2.75537400      6.83058600      1.30858200
H       2.72389600      6.12659200      0.46659100
H       1.82886500      7.41805200      1.24920500
C       3.96821100      7.76060700      1.15691000
H       4.89565200      7.18191000      1.16236200
H       3.90406200      8.31602200      0.21604400
H       4.01325600      8.47736400      1.98281000
C       2.73113700      4.75117100      4.86537100
H       3.74900400      4.64361600      5.25218800
H       2.18866700      5.33538200      5.61909900
C       2.07765800      3.37233500      4.71416600
H       2.09764300      2.83850200      5.66980500
H      -0.19038200      5.92051700      6.02541700
H       2.60687800      2.77238000      3.96762100
```

I1:

```
   106
 Generated by Multiwfn
Sn      5.12474100      7.88475000      5.85138800
Si      2.74610400      7.97600400      7.23430200
Si      0.70041100      7.69452900      6.02867100
Si      2.33453800      6.85297800      9.27128000
Si      2.73833700     10.30624000      7.66739000
C       7.08918700      5.57281700      3.24245600
C       7.12355600      5.17779200      5.70798400
C       7.42042600      6.62659200      7.60771500
C       6.66931400      6.29457000      6.45553800
```

C	7.24009700	7.87123700	8.42041400
C	6.66115800	4.79642700	4.33869700
C	8.18772200	4.38968500	6.16896200
H	8.50160100	3.54053100	5.56577000
C	5.71673100	3.19412000	2.79165100
H	5.20696500	2.24664900	2.62101900
C	8.49322200	5.83281200	8.03516700
H	9.05017400	6.13956200	8.91785100
C	6.07042300	3.98293800	1.70213700
C	6.77968000	5.15954200	1.94895500
H	7.12679800	5.75626600	1.10688600
C	7.79977800	9.08705900	7.97674700
C	6.76505700	7.76967700	9.74379500
C	0.73375100	8.18604300	4.20148100
H	1.15516900	9.18572100	4.05299600
H	-0.29587300	8.19556400	3.82086000
H	1.31321700	7.47803400	3.60261400
C	6.01811900	3.57083000	4.10430300
C	7.80427400	10.18789000	8.83552700
H	8.23786200	11.12358500	8.48640600
C	6.78359200	8.89405500	10.56769300
H	6.40857900	8.80870500	11.58643200
C	7.29024200	10.11663100	10.12701400
C	8.86605800	4.69685700	7.33693400
H	9.69846500	4.08493900	7.67364200
C	7.95168600	6.78860400	3.44894000
H	7.44022300	7.56198700	4.03275200
H	8.24611600	7.22372100	2.49026200
H	8.85851500	6.52665900	4.00709600
C	6.31530500	6.44442500	10.29856700
H	5.62997700	5.93576800	9.61473900
H	5.81962200	6.57232000	11.26314700
H	7.16945700	5.77355400	10.44376600
C	-0.62923900	8.80046500	6.80720400
H	-0.76652200	8.60785200	7.87492100
H	-1.58150600	8.59544400	6.30056400
H	-0.40459000	9.86353200	6.67820200
C	3.20397100	7.71398400	10.71442000
H	4.19503500	8.08985600	10.45054800
H	3.30626300	7.03474900	11.56986200
H	2.59943600	8.56864000	11.03773700
C	5.68776700	2.60980300	5.21918700
H	6.45295500	1.82592000	5.28550600
H	4.73103000	2.11015400	5.03261800
H	5.65020800	3.09866100	6.19575100
C	1.47909600	10.85709400	8.97120100
H	0.45742300	10.54248300	8.74394900
H	1.48978400	11.95253600	9.04271200
H	1.74432100	10.45824600	9.95605500
C	8.45351500	9.21529900	6.62584700
H	9.16554300	10.04520600	6.62397000
H	7.71124600	9.41140400	5.84000900
H	8.97809200	8.29709300	6.34641500
C	2.88374900	5.04612600	9.11011600
H	2.18041900	4.49796200	8.47147100
H	2.90532600	4.54989700	10.08837200
H	3.87541700	4.95729100	8.65403200
C	0.50547600	6.83324300	9.77315200
H	0.14538300	7.84928600	9.96879700
H	0.40307700	6.25964600	10.70408500
H	-0.14977400	6.37820700	9.02476800
C	2.40260600	11.26052400	6.06413100
H	3.11800300	10.97024200	5.28513900
H	2.51526400	12.33807200	6.23915600
H	1.39367900	11.08563100	5.67671700
C	4.42348100	10.87027800	8.29891200
H	4.70412900	10.34029100	9.21515600
H	4.40536600	11.94559500	8.51990700
H	5.21756300	10.68968000	7.56601300
C	-0.00463600	5.92695200	6.09545800
H	1.68885500	4.49243600	3.59070900
H	0.30179700	5.33768300	5.22611100
H	-1.10025800	5.98379900	6.08198700
C	5.70327200	3.58347000	0.29888500
H	5.55951800	2.50215200	0.21636200
H	6.47496700	3.88665400	-0.41554600
H	4.76671200	4.06529000	-0.00920100
C	7.25792100	11.33015000	11.01345100
H	8.06852300	12.02308700	10.76938300
H	7.33716700	11.05632600	12.06982900
H	6.31205900	11.87180500	10.88331900
C	3.99364000	7.10661300	3.65954800
C	3.71276000	6.14554100	4.36682800
C	3.96576400	8.15499400	2.63240700
H	3.33109900	7.76976300	1.82207400
H	3.43991900	9.02136000	3.05396700
C	5.31592200	8.59725800	2.07014200
H	5.84924600	7.75469200	1.62544800
H	5.15745700	9.35961800	1.30195800
H	5.93785700	9.02486300	2.86177000
C	3.21717100	4.97162100	5.06901800
H	4.04280400	4.47527200	5.58585900
H	2.53502600	5.31376400	5.85701600
C	2.50355300	3.99453300	4.12470000
H	2.08434200	3.16434300	4.70175800
H	0.28649500	5.37474800	6.99420100
H	3.20348000	3.59565000	3.38621000

T2:

106
Generated by Multiwfn

Sn	5.18760000	8.18283000	6.15832700
Si	2.65336500	7.30001600	6.85698600
Si	1.21704400	8.81171000	5.71453100
Si	1.48107500	5.29702100	7.46627200
Si	2.87235200	8.32917100	9.01555500
C	6.32323800	4.36212800	4.02530900
C	7.48887900	5.79814900	5.73941300
C	8.19363100	7.86555700	6.77120700
C	7.14802800	7.05460600	6.27416600
C	7.86884800	9.11969300	7.52245200
C	6.48181900	4.76187300	5.36376600
C	8.83438600	5.41636300	5.64016500
H	9.07489500	4.43638700	5.23290500
C	4.96484200	3.00483200	6.04288100
H	4.45305600	2.46455400	6.83760700
C	9.52883700	7.47208200	6.66350100
H	10.30944300	8.11977200	7.05748600
C	4.78910700	2.60151200	4.72041800
C	5.47867200	3.29389800	3.72528900
H	5.35490000	2.99454600	2.68542400
C	7.80373900	10.37262100	6.89230500
C	7.62428000	9.02202300	8.90756600
C	2.18333700	10.31548100	5.09749400
H	2.66635000	10.85293500	5.91988400
H	1.49536300	11.00640200	4.59284000
H	2.96094300	10.02015600	4.38580700
C	5.79952200	4.07265900	6.38160100
C	7.39372100	11.48727700	7.62915200
H	7.32257000	12.45156300	7.12831900
C	7.21682100	10.15491200	9.60894900
H	7.00734800	10.06865100	10.67394300
C	7.07114000	11.39300500	8.98011100
C	9.85250500	6.25418300	6.07681600
H	10.89068200	5.94496300	5.98903000
C	7.06251800	5.06007300	2.91505500
H	7.10364200	6.13953200	3.08421200
H	6.58291100	4.87147100	1.94990400
H	8.09984400	4.71114400	2.84515300
C	7.85429500	7.72503700	9.63729600
H	7.39579100	6.87758200	9.11889000
H	7.45804800	7.77260500	10.65473500
H	8.92836300	7.50913400	9.69704100
C	-0.16010500	9.46899300	6.83729500
H	-0.80407600	8.67058200	7.21838000
H	-0.78622700	10.16086700	6.25902500
H	0.23920400	10.01955000	7.69488300
C	2.23797000	4.54491100	9.02931000
H	3.26535100	4.21102500	8.85100200
H	1.64634900	3.66988000	9.32729600
H	2.25191300	5.24367000	9.87008200
C	5.99271600	4.43204600	7.83077600
H	7.05374200	4.56223300	8.06861400
H	5.58325600	3.65328900	8.47997700
H	5.49548400	5.37813400	8.07859500
C	1.35496200	8.22203700	10.15180800
H	0.46739800	8.67898200	9.70276200
H	1.58079000	8.76783300	11.07726500
H	1.10462300	7.19269200	10.42604700
C	8.21183900	10.54610100	5.45467600
H	9.25797200	10.87384200	5.39829600
H	7.60200200	11.30690900	4.95824200
H	8.13714900	9.61154400	4.89659400
C	1.25893000	3.82666500	6.29118300
H	0.68666700	4.07689900	5.39247600
H	0.69259400	3.06643100	6.84582800
H	2.20800400	3.37875000	5.98488200
C	-0.28954300	5.84349300	7.86416600
H	-0.33294900	6.66887400	8.57883900
H	-0.83738800	4.99321900	8.29109300
H	-0.81210900	6.15467200	6.95256700
C	3.25881600	10.17662200	8.84558900
H	4.13008800	10.35944100	8.20636100
H	3.48742200	10.57849600	9.84163700
H	2.41117800	10.74000900	8.44150800
C	4.32437700	7.56789500	9.95496800
H	4.17295300	6.50806800	10.18221000
H	4.48511500	8.10373300	10.89941400
H	5.24237400	7.65905400	9.36722700
C	0.36395200	8.04424400	4.20961800
H	2.51096300	6.29899800	2.08143100
H	1.08809000	7.77183200	3.43533500
H	-0.32871400	8.77898300	3.77952300
C	3.85667100	1.47553700	4.36494800
H	3.57113500	0.89992000	5.24993000
H	4.31408800	0.79375700	3.64029100
H	2.93621200	1.86307100	3.90919400
C	6.55983000	12.58588800	9.74009400
H	6.91780400	13.52195800	9.30163600
H	6.86797800	12.55156900	10.78946600
H	5.46268300	12.61050000	9.72106000
C	4.49313600	7.70710800	4.07814100
C	3.65794700	6.85399500	4.55279800
C	4.97640600	8.16697600	2.73445800
H	5.12654500	7.29425200	2.08550300
H	4.16311800	8.74963800	2.27918400
C	6.24032600	9.01611400	2.77356700
H	7.07782800	8.45219400	3.19844500
H	6.52421300	9.34281000	1.76783300
H	6.08603600	9.90375100	3.39580000
C	2.88950800	5.65868200	4.13554000
H	3.31807700	4.78900300	4.64556700
H	1.85596200	5.73866900	4.48594500

```
C       2.91821800      5.43690100      2.61972900
H       2.31606100      4.55762100      2.36429800
H      -0.21402600      7.15027000      4.46936100
H       3.93915800      5.26171000      2.27086600
```

P:

```
   106
 Generated by Multiwfn
Sn      4.31849900      7.51799700      3.88423500
C       6.61986800      6.64226500      0.64903100
C       6.34571500      5.36499300      2.80824100
C       6.37037500      5.44721400      5.22517200
C       5.90616000      5.95363400      4.00498000
C       5.83732400      6.01083200      6.50272000
C       5.79442300      5.91626100      1.53475200
C       7.25898200      4.30884800      2.83197900
H       7.58836100      3.85425400      1.90018300
C       3.88731800      6.42816600      0.12311800
H       2.82607800      6.31538200     -0.09232200
C       7.29482300      4.39766700      5.24344700
H       7.65588200      4.01575800      6.19638200
C       4.67806900      7.19761800     -0.72083600
C       6.04709000      7.27384100     -0.45200500
H       6.68600100      7.85481500     -1.11542900
C       6.52185700      7.03382000      7.18104700
C       4.63787100      5.49840200      7.03648400
C       4.42159500      5.76734800      1.23754200
C       5.97249100      7.56409200      8.35084700
H       6.49749900      8.37019800      8.86096200
C       4.14036400      6.02963400      8.22525500
H       3.21180100      5.63260100      8.63180600
C       4.78149600      7.07784400      8.88535500
C       7.73807700      3.83306000      4.05019700
H       8.44895700      3.01127000      4.07009200
C       8.10904300      6.72772400      0.85423100
H       8.37244600      6.86850900      1.90563900
H       8.53236900      7.54929800      0.27101900
H       8.59560100      5.80066700      0.52811900
C       3.88616500      4.39837700      6.33592700
H       3.41549400      4.77203100      5.41679300
H       3.09704100      3.99877600      6.97823900
H       4.55060100      3.57985700      6.04092100
C       3.53587000      4.79464100      1.98007200
H       3.43804900      3.87740500      1.38496800
H       2.53004300      5.20229900      2.12240600
H       3.94322100      4.51431000      2.95246800
C       7.86101800      7.52141500      6.69927800
H       8.66003700      6.86712200      7.07130100
H       8.06439700      8.53308600      7.06222500
H       7.92615000      7.51444100      5.60975700
C       4.09262500      7.90338700     -1.91273700
H       3.00112800      7.93708200     -1.85687200
H       4.36709000      7.39122700     -2.84295500
H       4.46647300      8.93004000     -1.98403200
C       4.17277100      7.69677100     10.11333800
H       4.92957900      8.19013200     10.72995200
H       3.65769300      6.94866900     10.72369700
H       3.43057400      8.45513800      9.83087600
C       5.63084200      9.27453800      3.81677700
C       5.09952200     10.42887500      4.30212100
Si      3.21038600     10.87388400      4.34820200
Si      2.19676600     10.36446900      2.23976600
Si      1.83827900     10.14324400      6.14793000
Si      3.01979500     13.26428600      4.45236700
C       1.35779900      8.67409200      2.06265100
C       3.52338100     10.48867700      0.89947300
C       0.81711700     11.59517500      1.82192200
C       2.06458000      8.34271300      6.65252300
C       0.03010900     10.35984500      5.63083400
C       2.12712500     11.17286800      7.71012200
C       3.68137400     14.05499200      2.86047500
C       1.20859800     13.77862700      4.66753800
C       3.90093200     14.14548200      5.88527400
H       0.68850000      8.71299700      1.19287200
H       2.08115300      7.87043300      1.89828700
H       0.76038500      8.40800000      2.94090000
H       3.06481000     10.37533000     -0.09104400
H       4.27640500      9.70133000      1.01800000
H       4.04046200     11.45414600      0.93055200
H       0.44261700     11.35434600      0.81871200
H      -0.02303500     11.50127700      2.51903400
H       1.14175300     12.63874300      1.81663000
H       1.75356800      7.65160800      5.86254200
H       3.10631400      8.11345900      6.90268900
H       1.45945400      8.14149500      7.54704600
H      -0.22118400      9.70439500      4.78995200
H      -0.62402000     10.09339900      6.47077200
H      -0.19917500     11.38828200      5.33741800
H       1.45058100     10.82505000      8.50133400
H       3.15344200     11.06712500      8.07945000
H       1.93312400     12.23763200      7.54685000
H       3.15147300     13.69977100      1.97123500
H       4.74639600     13.84119900      2.71614100
H       3.56292200     15.14479200      2.91220400
H       0.52580400     13.30631500      3.95826400
H       0.85929600     13.54912400      5.68032700
H       1.13844600     14.86576500      4.53366600
H       4.99071100     14.14021500      5.79705900
H       3.64012500     13.72285400      6.86048600
H       3.57050700     15.19252200      5.87730900
C       6.00596400     11.54040900      4.80865600
H       5.64551000     12.51757000      4.47136000
H       7.02255200     11.43984800      4.41839900
C       6.05384400     11.51468800      6.34171800
H       6.46524900     10.55990400      6.68771100
H       6.67370800     12.32783400      6.73527100
H       5.04937300     11.61748100      6.76521200
C       7.11054600      9.14243600      3.51100500
H       7.73576000      9.56650000      4.30887500
H       7.37617400      8.08198100      3.44879300
C       7.45558000      9.82200100      2.17820300
H       7.22806100     10.89277800      2.21208400
H       8.51793900      9.70106900      1.93807600
H       6.86784600      9.38420800      1.36461000
```

8. References

[S1] G. Rouquet, F. Robert, R. Méreau, F. Castet, Y. Landais, *Chem. Eur. J.* **2011**, *17*, 13904–13911.

[S2] M. Fischer, M. M. D. Roy, L. L. Wales, M. A. Ellwanger, A. Heilmann, S. Aldridge, *J. Am. Chem. Soc*. **2022**, *144*, 8908–8913.

[S3] M. Fischer, M. M. D. Roy, L. L. Wales, M. A. Ellwanger, C. McManus, A. F. Roper, A. Heilmann, S. Aldridge, *Angew. Chem. Int. Ed.* **2022**, *61*, e202211616.

[S4] C.-J. Frank Du, H. Hart, K.-K. Daniel Ng, *J. Org. Chem.* **1986**, *51*, 3162–3165.

[S5] A. Schulz, M. Thomas, A. Villinger, *Dalton Trans.* **2019**, *48*, 125–132.

[S6] C. Marschner, *Eur. J. Inorg. Chem.* **1998**, 221–226.

[S7] B. J. Cosier, A. M. Glazer, *J. Appl. Cryst.* **1986**, *19*, 105–107.

[S8] G. M. Sheldrick, *Acta Crystallogr. A* **2015**, *71*, 3–8.

[S9] G. M. Sheldrick, *Acta Crystallogr. C* **2015**, *71*, 3–8.

[S10] O. V. Dolomanov, L. J. Bourhis, R. J. Gildea, J. A. K. Howard, H. Puschmann, *J. Appl. Cryst.* **2009**, *42*, 339–341.

[S11] F. Neese, *Wiley Interdiscip. Rev.: Comput. Mol. Sci.* **2022**, *12*, e1606.

[S12] F. Neese, *Wiley Interdiscip. Rev.: Comput. Mol. Sci.* **2012**, *2*, 73–78.

[S13] F. Neese, *J. Comput. Chem.* **2023**, *44*, 381–396.

[S14] S. Grimme, A. Hansen, S. Ehlert, J. M. Mewes, *J. Chem. Phys.* **2021**, *154*, 064103.

[S15] J. W. Furness, A. D. Kaplan, J. Ning, J. P. Perdew, J. Sun, *J. Phys. Chem. Lett.* **2020**, *11*, 8208–8215.

[S16] E. Caldeweyher, S. Ehlert, A. Hansen, H. Neugebauer, S. Spicher, C. Bannwarth, S. Grimme, *J. Chem. Phys.* **2019**, *150*, 154122.

[S17] H. Kruse, S. Grimme, *J. Chem. Phys.* **2012**, *136*, 154101.

[S18] V. Barone, M. Cossi, *J. Phys. Chem. A*, **1998**, *102*, 1995–2001.

[S19] F. Cortés-Guzmán, R. F. W. Bader, *Coord. Chem. Rev.* **2005**, *249*, 633–662.

[S20] N. Mardirossian, M. Head-Gordon, *J. Chem. Phys.* **2016**, *144*, 214110.

[S21] M. Friede, S. Ehlert, S. Grimme, J. M. Mewes, *J. Chem. Theory Comput.* **2023**, *19*, 8097–8107.

[S22] F. Weigend, R. Ahlrichs, *Phys. Chem. Chem. Phys.* **2005**, *7*, 3297–3305.

[S23] F. Weigend, *Phys. Chem. Chem. Phys.* **2006**, *8*, 1057–1065.
